# The intrusive nature of the Châtelperronian in the Iberian Peninsula

**DOI:** 10.1371/journal.pone.0265219

**Published:** 2022-03-30

**Authors:** Joseba Rios-Garaizar, Eneko Iriarte, Lee J. Arnold, Laura Sánchez-Romero, Ana B. Marín-Arroyo, Aixa San Emeterio, Asier Gómez-Olivencia, Carflos Pérez-Garrido, Martina Demuro, Isidoro Campaña, Laurence Bourguignon, Alfonso Benito-Calvo, María J. Iriarte, Arantza Aranburu, Amaia Arranz-Otaegi, Diego Garate, María Silva-Gago, Christelle Lahaye, Illuminada Ortega

**Affiliations:** 1 Arkeologi Museoa, Bilbao, Spain; 2 Centro Nacional de Investigación sobre la Evolución Humana (CENIEH), Burgos, Spain; 3 Laboratorio de Evolución Humana, Dpto. Historia, Geografía y Comunicación, Universidad de Burgos, Burgos, Spain; 4 School of Physical Sciences, Environment Institute, and Institute for Photonics and Advanced Sensing (IPAS), University of Adelaide, Adelaide, SA, Australia; 5 Human Evolution Research Center, University of California, Berkeley, CA, United States of America; 6 Grupo de I+D+i EVOADAPTA (Evolución Humana y Adaptaciones Económicas y Ecológicas durante la Prehistoria), Dpto. Ciencias Históricas, Universidad de Cantabria, Santander, Spain; 7 Department of Archaeology, University of Cambridge, Cambridge, United Kingdom; 8 Dept. Geología, Facultad de Ciencia y Tecnología, Universidad del País Vasco-Euskal Herriko Unibertsitatea (UPV/EHU), Leioa, Spain; 9 Centro UCM-ISCIII de Investigación Sobre Evolución y Comportamiento Humanos, Madrid, Spain; 10 Sociedad de Ciencias Aranzadi, Donostia-San Sebastián, Spain; 11 Departamento de Mineralogía y Petrología, Facultad de Ciencias Geológicas, Universidad Complutense de Madrid, Madrid, Spain; 12 Departamento de Ecología y Geología, Facultad de Ciencias, Universidad de Málaga, Málaga, Spain; 13 Inrap/AnTet Arscan UMR7041, Lotissement Actipolis Impasse sur rue Dionysos, Villeneuve-les-Béziers France; 14 Geography, Prehistory and Archaeology Department, University of the Basque Country Vitoria-Gasteiz, Spain; 15 Basque Foundation for Science (IKERBASQUE), Bilbao, Spain; 16 Múseum National d’Histoire Naturelle, UMR 7209, Archéozoologie, Archéobotanique: Societés, Pratiques et Environments (AASPE), Paris, France; 17 Dept. of Cross Cultural and Regional Studies, University of Copenhagen, Copenhagen, Denmark; 18 Instituto Internacional de Investigaciones Prehistóricas de Cantabria (IIIPC, Gobierno de Cantabria, Universidad de Cantabria, Santander), Santander, Spain; 19 Professeure–Géochronologie, Université Bordeaux Montaigne, IRAMAT-CRP2A UMR 5060, Pessac, France; 20 Institut National de Recherches Archeologiques Preventives (INRAP), Paris, France; Max Planck Institute for the Science of Human History, GERMANY

## Abstract

Multiple factors have been proposed to explain the disappearance of Neandertals between ca. 50 and 40 kyr BP. Central to these discussions has been the identification of new techno-cultural complexes that overlap with the period of Neandertal demise in Europe. One such complex is the Châtelperronian, which extends from the Paris Basin to the Northern Iberian Peninsula between 43,760–39,220 BP. In this study we present the first open-air Châtelperronian site in the Northern Iberian Peninsula, Aranbaltza II. The technological features of its stone tool assemblage show no links with previous Middle Paleolithic technology in the region, and chronological modeling reveals a gap between the latest Middle Paleolithic and the Châtelperronian in this area. We interpret this as evidence of local Neandertal extinction and replacement by other Neandertal groups coming from southern France, illustrating how local extinction episodes could have played a role in the process of disappearance of Neandertals.

## 1. Introduction

The disappearance of Neandertal populations remains a complex, ongoing debate, with multiple factors purported to have affected survivorship between ca. 50 and 40 kyr BP, including endogenous causes, climate change and the arrival of Homo sapiens in Eastern Europe [1–5]. In the midst of this extinction process, several new techno-cultural complexes appeared in Europe, some of them introduced by Homo sapiens, while others were developed by Neandertal populations. One of these complexes is the Châtelperronian, which is found from the Paris Basin to the Northern Iberian Peninsula. The origins of the Châtelperronian remain widely debated. Some argue that it originated in Western Europe as an evolution of Middle Paleolithic technology [6, 7], possibly under the influence of the growing population of *Homo sapiens* in Eastern and Central Europe [5, 8, 9], while others continue to doubt the links between the Châtelperronian and Neandertals, and thus propose that this technocomplex could have been made by *Homo sapiens* populations [10, 11]. However, recent technical developments have significantly advanced these discussions by presenting paleoproteomic evidence for Neandertal authorship of the Châtelperronian at Arcy-sur-Cure [12]. The fact that the only human remains directly dated within the Châtelperronian age range in Western Europe have Neandertal affinities [13–15] further strengthens this link. In this study we present a new and significant Châtelperronian site, Aranbaltza II, located in the southernmost area of distribution for this complex. A detailed technological analysis of the lithic assemblage shows that there is no technological link with previous Middle Paleolithic technology in the Northern Iberian Peninsula. This, coupled with evidence of a chronological gap between the latest Middle Paleolithic (MP) and the Châtelperronian (CP) locally [16], suggests that Neandertal groups with MP technology had abandoned the region before the Neandertal groups with CP technology, likely coming from Aquitaine, crossed the Pyrenees and expanded through the Cantabrian Region. This illustrates how episodes of local extinction and population replacement could have played a role in the process of Neandertal disappearance.

## 2. Results

### 2.1 The site of Aranbaltza II

**Fig 1:**
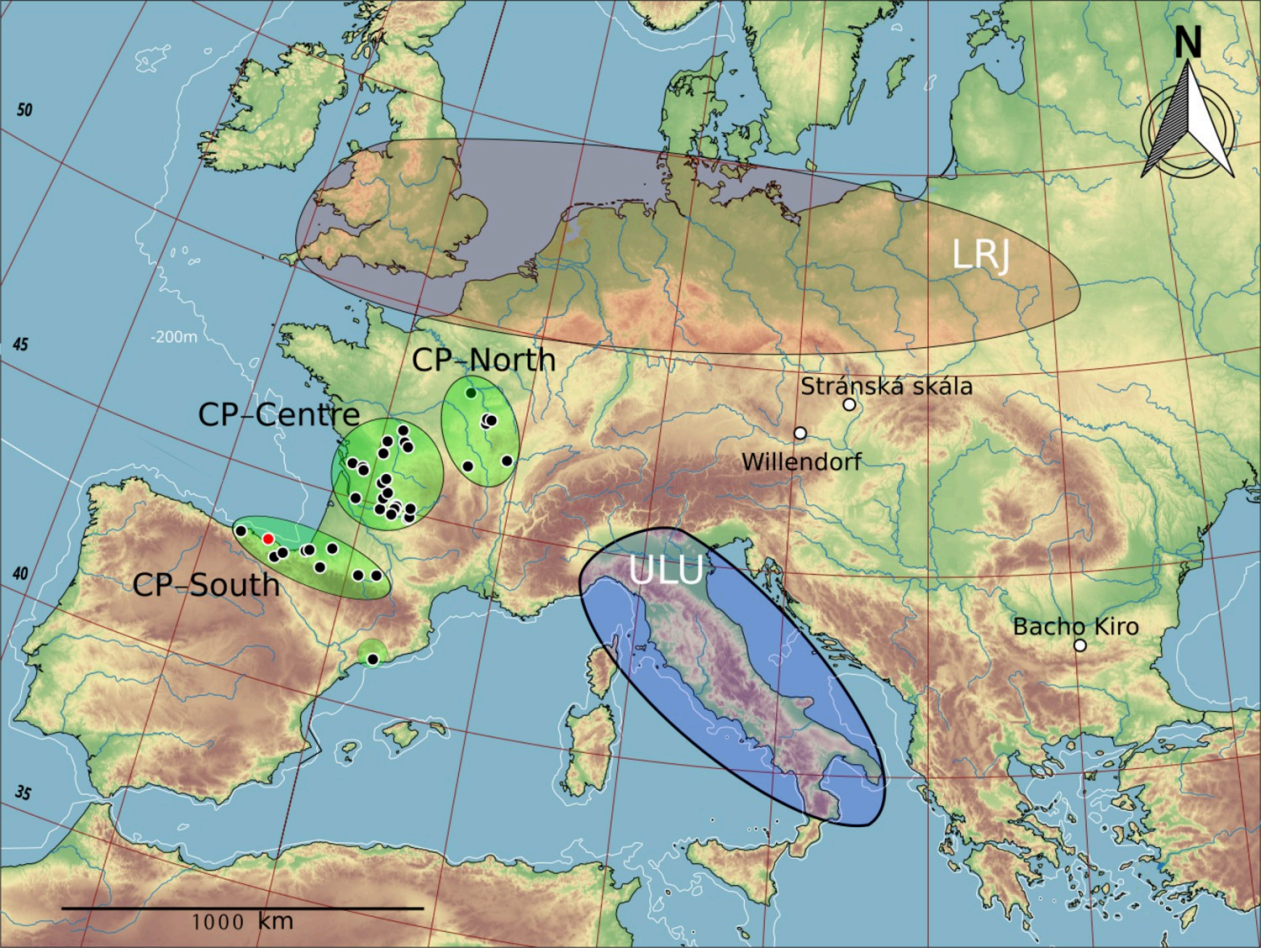
Distribution map of the so-called transitional technocomplexes. CP: Châtelperronian; LRJ: Lincombian-Ranisian-Jerzmanowician; ULU: Uluzzian. Base cartographic data obtained from the European Environment Agency (free of use, downloadable at https://www.eea.europa.eu/ds_resolveuid/070F2DAD-1AED-4B9B-950F-0047E5ADDF35. Rivers and bathimetry obtained from Natural Earth (free for use). DEM built up with QGIS 2.18. Infographics made with Inkscape.

**Fig 2:**
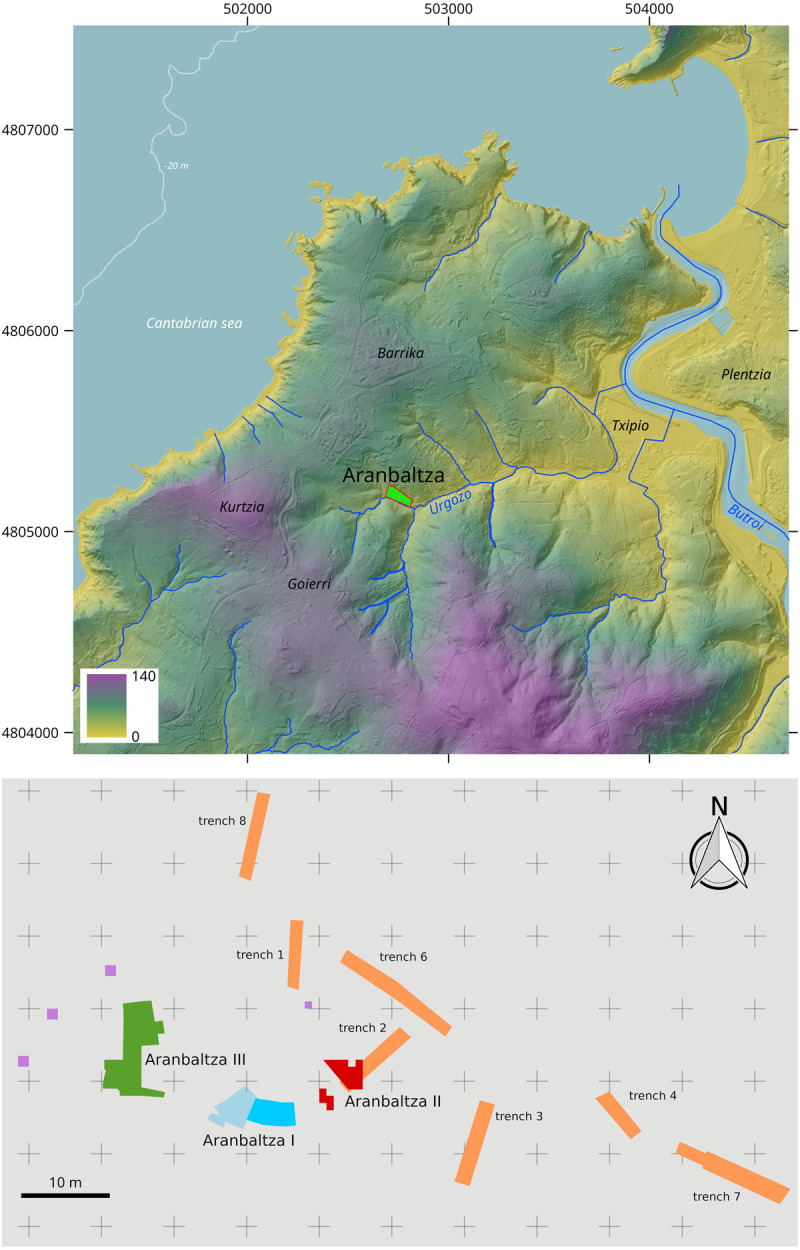
Aranbaltza location maps. **A)** Digital Elevation Model showing the position of Aranbaltza site; **B)** Excavation plan, with the area of the excavated Châtelperronian deposits in red. Base cartographic data obtained from National Geographic Institute of Spain (IGN) under the Creative Commons License CC-BY 4.0. DEM built up with ArcGis 10.3. Infographics made with Inkscape.

**Fig 3:**
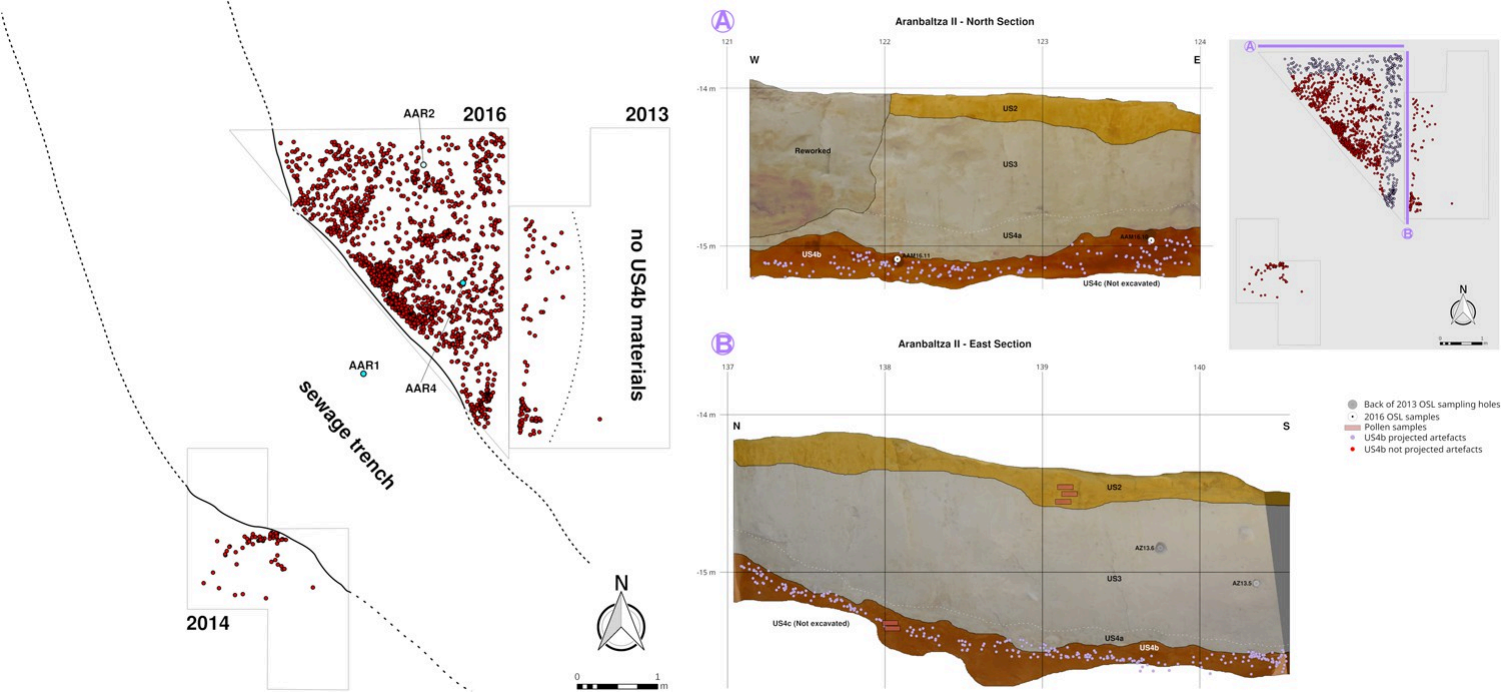
Excavation map and sections. A) Detailed map of the excavated Châtelperronian deposits with the position of the sedimentological cores and the coordinated archaeological remains; B) North and east section of Area 3 excavation with the projected position of the archaeological materials and the positions of the pollen samples and the dated OSL samples.

The site of Aranbaltza (Northern Iberian Peninsula) is located in the Basque coastal region, in a short valley close to the current coastline (800 m NW) (Figs 1 and 2). During Marine Isotope Stage (MIS) 3, sea level was 60–70 m lower than today, with the coastline ca. 4 km further offshore according to recently obtained bathymetric surveys [17]. The site is located in an old sand quarry complex (Ollagorta-Aranbaltza) that exploited Pleistocene fluvial sand deposits overlaying Upper Cretaceous bedrock (marine marls and limestone alternating with intercalated volcanic layers) (Section SI-3 in S1 File). It was discovered in 1957 by A. Aguirre, and in 1959 J.M. Barandiaran opened several test pits, finding a sandy level (C) containing Châtelperronian material [18] (Section SI-1 in S1 File). In 2012 a large Châtelperronian collection from the Aranbaltza II disturbed sediments was studied [19] (Section SI-5 in S1 File). The latest excavation project, which started in 2013, has since revealed a complex of archaeological sites (Aranbaltza I, II and III) with occupations ranging from Middle Paleolithic to recent prehistoric times (Fig 2) [20]. Between 2013 and 2016, the Aranbaltza II site was excavated in three different areas (ca. 18 m^2^) (Area 1, 2 and 3) (Section SI-2 in S1 File) (Fig 3). All necessary permits were obtained for the excavation and study of materials. The archaeological collection of Aranbaltza II US4b is currently held at the Archaeologi Museoa in Bilbao (Basque Country, Spain).

### 2.2 Stratigraphy

**Fig 4:**
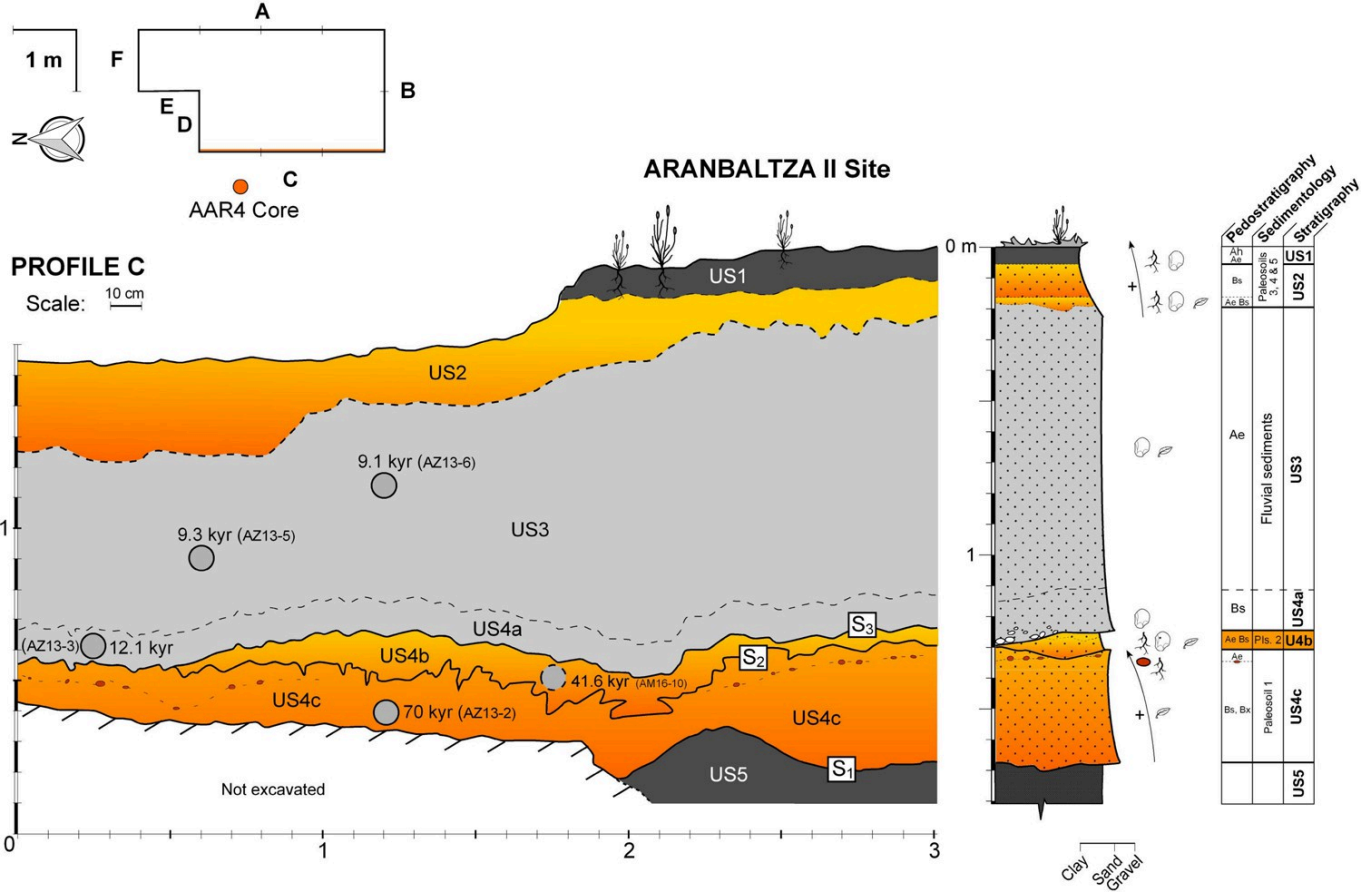
Stratigraphic section between Area 1 and 3 (section C) and synthetic stratigraphic section with the position of the dated OSL samples Stratigraphy. A detail photograph of the sequence in corner C/D is presented in S6 Fig in S1 File.

The 2 m sedimentary sequence excavated at Aranbaltza II contains a succession of aggradational, erosive and paleosoil formation events. The Châtelperronian occupation is included in a sedimentary unit (US4) formed from three different sand accumulation intervals (US4a, b and c subunits) related to enhanced flood activity in the nearby river channel (Fig 4). The sands from US4b have an intense orange hue and mineralized bioturbations typical of Fe and Al illuviation in podzols.

### 2.3 Chronology

Single-grain optically stimulated luminescence (OSL) dating undertaken on Unit 4c indicates that a series of aggradation events and paleosol development phases took place between late marine isotope stage (MIS) 5 and early MIS 3 (78.1 ± 5.8 ka to 58.2 ± 4.6 ka; Section SI-9 in S1 File) prior to the Châtelperronian occupation of Aranbaltza II. Several OSL ages obtained for US4b and US4c provide bracketing (indirect) age constraint for the US4b layer, with sample AAM13-10 providing the closest and most secure estimate for the timing of the Châtelperronian occupation 43.5 ± 2.9 ka (1σ) (Section SI-9 in S1 File).

### 2.4 Lithic assemblage

**Fig 5:**
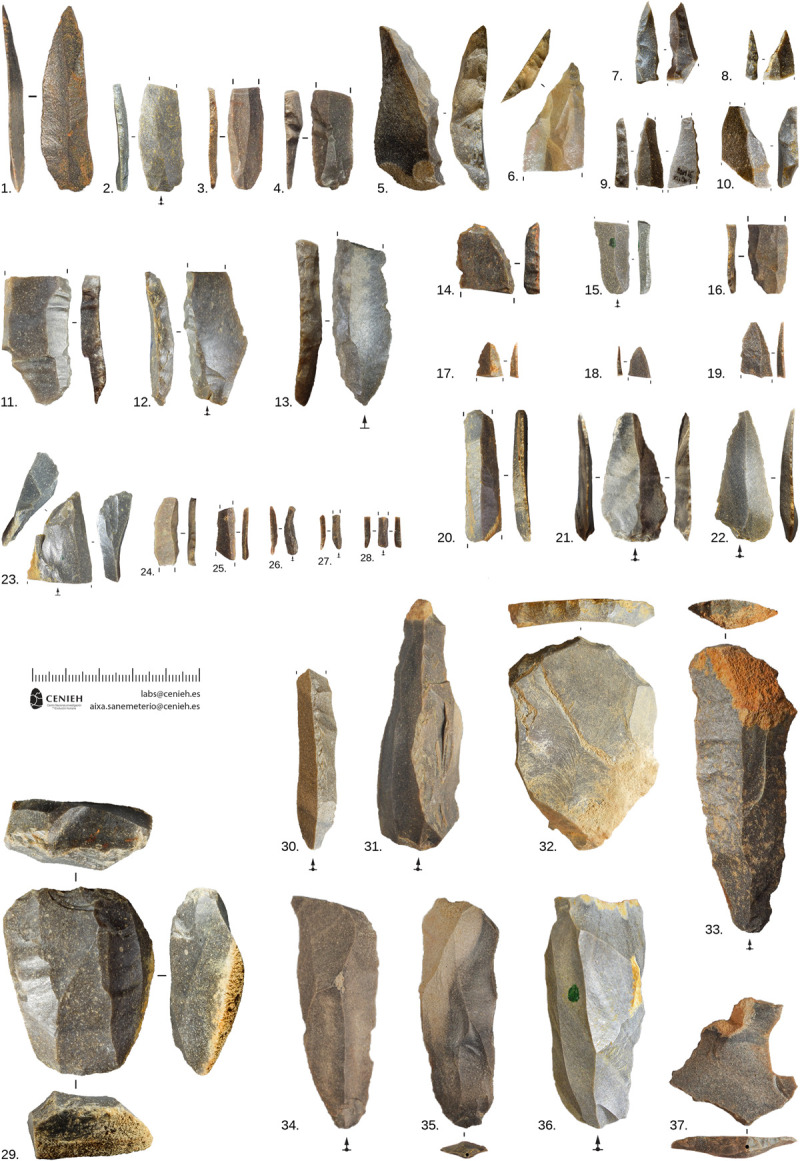
Châtelperronian lithic assemblage. Aranbaltza II, US4b (Châtelperronian) archaeological materials. 1) Complete thin Châtelperronian point; 2–4) Proximal fragments of thin Châtelperronian points; 5) Thick Châtelperronian point, with the point made in the proximal part; 6) Distal fragment of wide Châtelperronian point; 7–10) Distal fragments of Châtelperronian points: #9 exhibits axial diagnostic impact fracture; 11–13) proximal fragments of backed blades, possible Châtelperronian points; 14) Distal fragment of backed blade; 15,16,20) Fragments of marginally backed blades; 17–19) Distal fragments of marginally backed points; 21–22) Marginally backed points; 23) Nucleiform burin; 24–28) Backed bladelets; 29) Bidirectional blade core for the production of pointed blades; 30–31) Unidirectional crested blades; 32) Endscraper on natural fragment; 33) Endscraper on blade; 34–35) Blades; 36) Overshot blade dragging an opposed platform; 37) Platform rejuvenation flake. Figure made by Aixa San Emeterio and Joseba Rios-Garaizar.

The archaeological assemblage is composed exclusively of lithic artifacts, which are found in a minimally disturbed accumulation (Section SI-4 in S1 File). We have analyzed a well-preserved assemblage of 5686 lithic remains, including all the lithic remains bigger than 1 mm (Fig 5), primarily made from local flint (Flysch flint), but also small quantities made from non-local flint, including the Salies-de-Béarn flint variety (SW France, >150 km away) (Section SI-6 and S25 Fig in S1 File). Other materials appear in very low proportions, including 30 small lumps of ochre (S34 Fig in S1 File). A large part of the assemblage (60.4%) is composed of natural blocks, chunks, thermal flakes and chips under 10 mm size, reflecting intensive block testing, core flaking and tool production. The cortical flakes and blades generated in the initial stages of core configuration are particularly abundant (S5 Table in S1 File). Cores appear in relatively low numbers, which is similar to other open-air Châtelperronian sites (Grigoletto et al., 2008). Most of the cores have been exploited to produce bladelets (n = 10) and blades (n = 7), but there are also some non-standardized “expedient” flake cores (n = 4) and some core rough-outs (n = 6), tested blocks (n = 3) and exhausted cores (n = 1). Among the blade cores, 4 are typical [21–24] bidirectional ones with two, opposed flaking surfaces that are not strictly parallel (bipolaire décalé), which were used for the production of pointed asymmetrical blades (Fig 5: 29; S26 Fig in S1 File: 1). The unidirectional cores have typical [25, 26] quadrangular cross-sections for the production of asymmetrical blades (S26 Fig in S1 File: 2). All the bladelet cores are unidirectional: five of them are nucleiform burins (Fig 5: 23; S28 Fig in S1 File: a; S29 Fig in S1 File: 1–2), and the other five have been made on blocks (S28 Fig in S1 File: b, c; S29 Fig in S1 File: 3, 4 and 6). These cores have produced narrow (ca. 4 mm) bladelets and are similar to the bladelet production described in Quinçay (Fig 2: 24–28; S28 Fig in S1 File: 1–19) [26]. Blanks are dominated by narrow (6.4–13.5 mm width) and wide (>13.6 mm) blades and bladelets (<6.4 mm width) (S30 Fig and S6 Table in S1 File). Bladelets show great morphological variability, which is also reflected in the variability in terms of productivity and morphology of bladelet cores. Flakes are abundant: they have been mostly obtained from unidirectional elongated flake cores, from non-standardized “expedient” flake cores, or as part of blade core preparation and maintenance. Typical Levallois, Discoid or Quina flakes are all absent from the assemblage.

**Fig 6:**
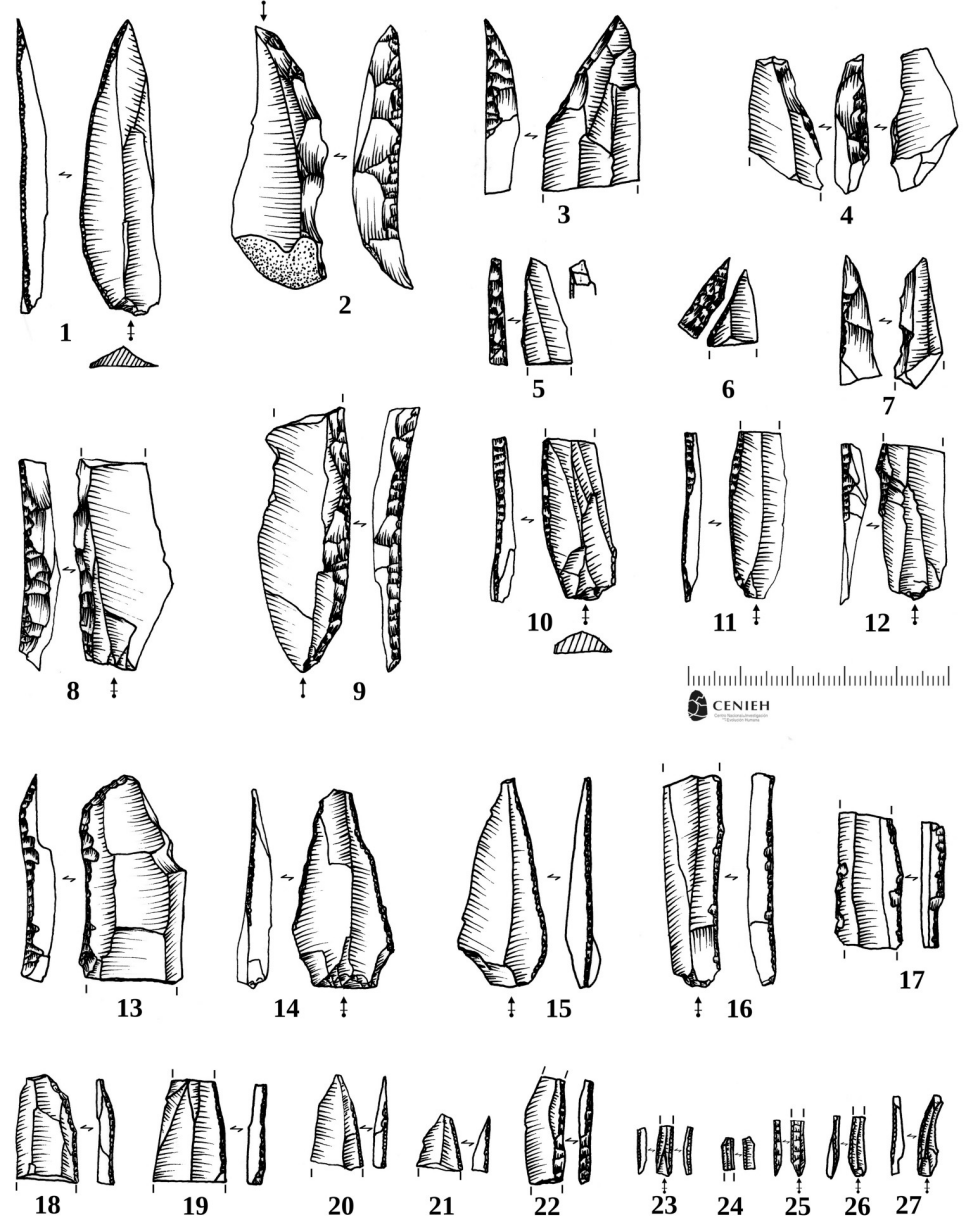
Châtelperronian retouched tools. Selected retouched tools from US4b. 1) Complete thin Châtelperronian point; 2) Thick Châtelperronian point, with the point made in the proximal part; 3) Distal fragment of wide Châtelperronian point; 4–7) Distal fragments of Châtelperronian points: #5 exhibits axial diagnostic impact fracture; 8–12) Proximal fragments of backed blades, possible Châtelperronian points; 10–12) Proximal fragments of Châtelperronian points; 13) Distal fragment of backed blade; 14–15) Marginally backed points; 16–18) Fragments of marginally backed blades; 19–21) Distal fragments of marginally backed points; 22–26) Backed bladelets: #24 presents typical Dufour ventral semi-abrupt retouch. Drawings made by Joseba Rios-Garaizar.

There are 117 retouched tools, all but one made on Flysch flint (S7 Table in S1 File). Among them, we found typical marginally backed blades (n = 26) [22, 24, 25, 27] including total, partial, pointed and inversely retouched blades (Fig 2: 15–20; Fig 6: 14–21); typical Châtelperronian points (n = 10) (Fig 5: 1–10; Fig 6: 1–7, 10–12), some of them broken during the fabrication process [28]; blade fragments with curved backs (n = 8) which are very likely proximal fragments of Châtelperronian points (Fig 5: 11–13; Fig 6: 8–9); and typical wide endscrapers (n = 2) (Fig 5: 32). Among the retouched bladelets, as identified at other Châtelperronian sites like Quinçay [26], there is a single typical Dufour (Fig 6: 24), five backed, five marginally backed, two partially retouched and one truncated bladelet (Fig 5: 24–28; Fig 6: 22–27). Other tools such as retouched blades, burins, borers, truncations, notches, denticulates, sidescrapers, splintered pieces, marginally backed flakes and retouched flakes represent almost half of the retouched toolkit at the site (Fig 5: 33; S32 Fig in S1 File), but these are not very standardized and probably represent opportunistic and non-specific tool making and use at the site.

## 3. Discussion

The technological and typological features of the Aranbaltza II US4b archaeological assemblage show direct parallels to other Châtelperronian open-air [22–24, 29] and cave/rock-shelter sites [23, 25, 30, 31]. Although knapping was the main activity at the site, other tasks were probably also performed alongside this, as happens at other Châtelperronian open-air sites [24, 29, 32]. The units below US4b are archaeologically sterile, precluding any possible admixture of the Châtelperronian assemblage with Middle Paleolithic materials, which are otherwise very abundant at Aranbaltza I and III [20, 33]. Moreover, the lithic assemblage from Aranbaltza II US4b does not preserve any technological traits that would link it with the local or regional Middle Paleolithic. In particular, typical Middle Paleolithic tools (denticulates or sidescrapers on flakes) are rare, and the flakes at Aranbaltza II were obtained as a by-product of blade core configuration and maintenance, or as the result of non-systematic and rather opportunistic flake production. No typical Middle Paleolithic Levallois, Discoid or Quina products have been identified at Aranbaltza II US4b. Furthermore, blank production at Aranbaltza II is basically oriented towards the production of blades and bladelets, following the same production schemas seen at classic sites such as Roc de Combe, La Côte and Quinçay [21, 26], Morin level 10 [34], and also evidenced in assemblages such as Labeko Koba [27], Ekain [35], and Cova Foradada [36]. Blade and bladelet production in the regional Middle Paleolithic are very rare, and never formed part of the structural productions for the Mousterian Neandertals [37–40]. Additionally, there are no MTA-B assemblages in the Cantabrian Region [41], while and backed tools associated with Mousterian assemblages, like the Abri Audi knifes, are very rare in the Northern Iberian Peninsula [33, 42, 43].

Elsewhere in the Northern Iberian Peninsula there are several Châtelperronian cave sites with small lithic assemblages [35] that have no technological links with the previous Late Middle Paleolithic [41, 44], suggesting that the Châtelperronian was not derived from previous Iberian Peninsula Middle Paleolithic tradition as has been suggested for SW France [6]. There is, however, one exception to this trend, namely level 10 of Cueva Morín, which is characterized by the use of different raw materials, including flint and coarse-grained quartzite, ophite and sandstone, and by the combination of typical Middle Paleolithic (Discoid) and Châtelperronian production schemas [34, 45]. Cueva Morín level 10 is situated directly between the Late Middle Paleolithic (level 11) and Protoaurignacian (level 9). This assemblage has been interpreted as an admixture of Middle Paleolithic, Châtelperronian and Protoaurignacian materials [46, 47], though other explanations have also been invoked, including the adaptation to raw material availability and techno-functional demands [45]. Whatever be the case, a part of this collection, which is made basically of flint, represents a clear Châtelperronian assemblage with some features that are very similar to the ones observed at Aranbaltza II [34]. Additionally, a few sites have provided Châtelperronian points in Middle Paleolithic contexts, such as Ermitons, Reclau Viver or L’Arbreda [48], or in disturbed or unclear contexts, such as A Valiña, La Güelga, El Pendo, Cueva Oscura, Santimamiñe, Mugarduia and Abauntz [49–55]. However, in terms of Châtelperronian occupation dynamics, these sites must be considered with caution.

The settlement system during the Châtelperronian around the Bay of Biscay is characterized by (i) a combination of different site types, including open-air encampments situated near flint outcrops, which are basically oriented to the production of Châtelperronian points (like Aranbaltza II, Le Basté and Bidart); (ii) hunting camps with small assemblages and relatively abundant Châtelperronian points (like Labeko Koba, Ekain or Brassempouy); and (iii) less abundant, encampments in caves like Cueva Morín. The identification of a Châtelperronian occupation at Aranbaltza II that basically functioned as a flint workshop changes the perception that the Châtelperronian presence in the Northern Iberian Peninsula was marginal [35]. The poor development of open-air Paleolithic archaeology in the Cantabrian Region, the geomorphological limitations for the preservation of such sites [56] and the fact that the Châtelperronian probably lasted only a few hundreds of years here, could explain the limited record and hence the low visibility of the Châtelperronian in this region. This settlement pattern has not been identified in the regional Late Middle Paleolithic: none of the Late Middle Paleolithic sites in the Northern Iberian Peninsula has been interpreted as hunting camps, and much of the open-air occupations near flint outcrops seem less focused on the production of specific lithic tools in comparison to Aranbaltza II, Le Basté or Bidart (see Le Prisse or Aranbaltza I and III as examples) [33, 57].

**Fig 7:**
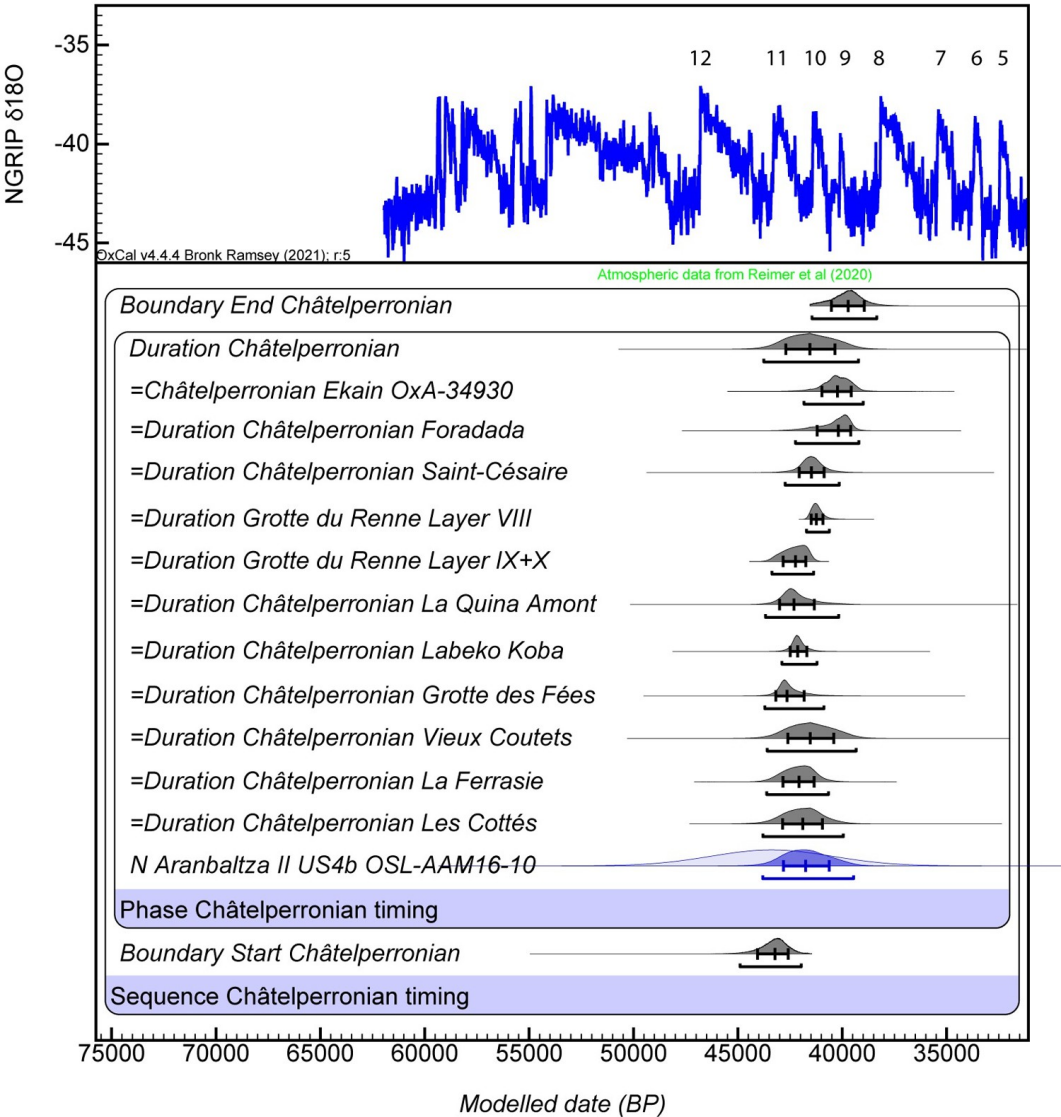
Bayesian modelling results for the timing of the Châtelperronian culture in southwest Europe. The modelled durations of the Châtelperronian culture (shown in grey) for sites that contain multiple dating results have been derived from site-specific Phase models using the Date command (see S39 Fig in S1 File for details). The site-specific modelled durations have been combined with the new OSL age for the Aranbaltza Châtelperronian culture (unmodelled and modelled OSL age distributions shown as light blue and dark blue, respectively) as part of the regional Châtelperronian Phase model shown here. The combined age range for the Châtelperronian culture of southwest Europe (“Duration Châtelperronian”) has been calculated from the modelled posterior probabilities of the start and end boundaries of this Phase model (also shown in grey) using the Date function. Bayesian modelling has been undertaken in OxCal v.4.4.4 [70], assuming each likelihood has a 5% prior probability of being an outlier within a general t-type outlier model [71]. The median ages and 1σ uncertainty ranges are shown for the modelled probability distributions. The 95.4% ranges of the highest posterior probabilities are also indicated by the broader horizontal bars underneath the probability density functions. The modelled durations are compared against the NGRIP GICC05 δ18O record, with interstadials (milder climatic periods) numbered accordingly.

The chronology of Châtelperronian occupations in the Northern Iberian Peninsula and the Western Pyrenees is primarily based on ^14^C ages from Labeko Koba IXinf (S12 Table in S1 File), which place this occupation around 43,850–40,950 cal BP [58]. Unfortunately, all efforts to date Cueva Morín level 10 have been fruitless, and the age obtained from Ekain level Xa, 34,350±550 BP (OxA-34930, 40,700–37,700 cal BP), is relatively young compared with Labeko Koba and the earliest Aurignacian for the region [16, 58, 59]. Three 14C ages obtained from charcoal samples from Cova Foradada level IV are statistically indistinguishable from each other and indicate a similarly young age range of 41,450–36,900 cal BP (S12 Table in S1 File) [36]. A significantly younger fourth age of 31,900±200 BP (36,750–35,750 cal BP) has also been obtained for Cova Foradada (Mediterranean margin of the Iberian Peninsula) level IV, though this is probably an artifact of the charcoal pre-treatment procedure [36]. The OSL age of Aranbaltza II US4b (~43.5 ± 2.9 ka; 1σ), which is probably slightly older than the occupation itself, is systematically younger than the latest Middle Paleolithic in the region, dated to 48–45 kyr cal BP [16]. Comparison of the Aranbaltza II US4b OSL age with all reliable and stratigraphically well-constrained Châtelperronian AMS 14C, TL and OSL ages from France and Iberia shows a perfect fit with the time range of the Chatelperronian culture for southwest Europe, which our Bayesian model reveals as occurring between ca. 43,760–39,220 kyr (Fig 7; S14 Table in S1 File; Section SI-9 in S1 File).

The original nature of the Châtelperronian assemblages from Aranbaltza II and Labeko Koba, together with the chronological gap between the latest Middle Paleolithic and the Châtelperronian in the region, and the absence of any link in lithic management and settlement strategies between the Châtelperronian and the regional Late Middle Paleolithic reveal that there is a discontinuity between the Middle Paleolithic and the Châtelperronian, and that the latter is intrusive in the Northern Iberian Peninsula (i.e., it originated elsewhere and was then introduced to the region).

One possible scenario explaining this discontinuity could be the local extinction of Neandertals in the Cantabrian Region or the abandonment of this territory by Neandertal groups ca. 45 kyr BP [16]. The Cantabrian Region is a rather isolated coastal area, and due to its abrupt terrain, the western and central parts are not very well-connected with their neighboring areas (the Atlantic facade and the Northern Iberian Plateau (Meseta)). In comparison, the eastern part of the Cantabrian Region has easier access to the Ebro Valley, the Northern Iberian Plateau and the rest of Europe, but even this would have been conditioned by climate deterioration between ca. 50–40 kyr BP [60–62]. The pollen data from sediment core MD04-2845, obtained in the Bay of Biscay [63], and glacial and limnological proxies from the Cantabrian mountains [64], show a succession of temperate and intense cold events between ca. 50 kyr and 39 kyr which dramatically affected tree cover during the coldest periods, likely affecting fauna and human groups. Various proxies (e.g. lithic technology management, subsistence strategies and landscape use) show that Neandertal groups increased their residential mobility and enlarged their exploitation areas during harsh climatic episodes of MIS4-3, which has been linked to a decrease in ecosystem productivity [38, 42, 65], and would have led to the same territory only being able to sustain smaller populations. Lower population and larger territories likely meant less contact between groups. The studies that suggest the presence of consanguinity in El Sidrón Late Middle Paleolithic populations [66] would be consistent with this pattern: limited gene flow, sporadic contacts with other Neandertal communities, and the prevalence of genetically related diseases. Under these circumstances, the presence of Neandertal groups in the region after ca. 48 kyr BP was probably very sporadic, as has been corroborated by the number of available ages [16], and the central and eastern part of the Northern Iberian Peninsula became an available expansion area for the groups that developed the Châtelperronian cultural complex in southern France. Very likely, these groups were Neandertals as it is suggested by the accumulated evidence of Nendertal authorship of the Châtelperronian [12–15]. The earliest Protoaurignacian, traditionally associated to *Homo sapiens*, appeared in the Northern Iberian Peninsula shortly after the first regional Châtelperronian recognized at Aranbaltza II. The Protoaurignacian has been identified in El Castillo and Labeko Koba by ca. 43–42 kyr cal BP [59], roughly overlapping with the ages of Labeko Koba level IX Châtelperronian (ca. 43–41.4 kyr cal BP) [16, 58], and the Aurignacian is, shortly thereafter, present in the Western Iberian Peninsula [67]. This probably reflects a relatively short duration for the Châtelperronian in the Northern Iberian Peninsula and a quick replacement of the last Neandertals by the first *Homo sapiens* arriving in Western Europe. This scenario would be consistent with the complex evolutionary (historical) trajectories of late Neandertal groups just before their extinction [68, 69].

## 4. Materials and methods

### 4.1 Sedimentology and coring

Cores of 2 m depth were collected near the excavation profiles using a Van Walt/ Eijkelkamp window corer, which permits recuperation of the sedimentary record by successive 1 m depth operations. Each core was replicated to ensure they were representative. Once collected, the samples were sealed and stored at 3–4°C. The AAR4 core, obtained near the southern profile of the excavation area (1 m to the south), was selected as representative of the complete stratigraphy observed in the profiles of the excavation pit. The sedimentological study consisted of the stratigraphical characterization and the description of the sedimentary facies observed in both the excavation profiles and in the AAR4 core.

### 4.2 Granulometry and grain morphology analysis

The cores were split into two halves and imaged with a high-resolution digital camera in an XRF core-scanner. One half of the core was sampled every 10 cm for XRD and particle size and texture analysis.

The texture of the samples was analyzed using particle size sieving and laser diffraction techniques. For the sieving techniques, a φ size sieve, range –3 φ to 4 φ, was used. A Beckman Coulter LS13 320 laser diffraction particle size analyzer was used to measure the particle size of the silt and clay fraction. Particle size was classified following the scheme of Blott and Pye [70]. The morphological analysis of sand particles was performed using a Malvern Morphologi G3 particle characterization system (Malvern Instruments, Malvern, UK). For this purpose, the grains in the sample were separated and dispersed over a glass plate by air injection using a Sample Dispersion Unit (SDU). Next, the instrument took high-resolution gray-scale images of the complete glass plate using a motorized slide. The particles were identified using a gray-scale threshold and then the shape parameters were calculated. The analyses were conducted following the procedures described by Campaña et al. [71]. After each analysis, post-processing of the data was performed. This post-processing consisted of the elimination of joined particles, non-minerals (i.e. organic matter, ambient dust) and poorly identified particles. Several size and shape parameters were measured for each grain. The shape parameters used were aspect ratio, high sensitivity circularity and convexity. The aspect ratio is the ratio of particle width to length, and ranges from 0 to 1. High sensitivity circularity (HSC) indicates the similarity of a particle to a circle and the values range from 0 (extremely narrow rod) to near 1 (perfect circle). Finally, convexity is calculated from the ratio between the particle perimeter and the perimeter of its convex hull. This shape parameter indicates the roughness of the particle [71, 72] and its value ranges from 0 (extremely rough) to 1 (extremely polished).

### 4.3 Mineralogical analysis: X-Ray Diffraction (XRD)

Bulk sample mineralogy was determined by powder X-ray diffraction (XRD) using a Bruker D8 Discover DAVINCI diffractometer at the Science and Technology Park, Burgos University. Air-dried samples were sieved at 2 mm, finely ground in an agate mortar, and processed using a continuous scan range of 2°-80° 2θ, a 0.05° step size and 1 second time per step. The sample was irradiated with Cu Kα radiation (ceramic X-ray tube KFL-Cu, 40 kV, 40 mA) with a programmable divergence slit, and a LynxEye detector was employed. Semi-quantitative estimates were calculated from peak areas on XRD patterns using DIFFRACplus basic EVA software with the ICDD database.

### 4.4 Organic carbon analysis

The organic carbon present in the sediments was measured through thermal oxidation method or Loss On Ignition (LOI). 0.7–0.8 g of milled and homogenized sample was heated to 110°C for 14 hours inside the muffle (Heron HD-230 PAD) and then to 550° for 5 hours. The weight of the sample was measured after each heating using an analytical balance and the lost organic C calculated in accordance with Santisteban et al. [73].

### 4.5 OSL dating and bayesian modelling

Eight single-grain OSL dating samples were collected from Units 3 to 4c to provide estimates of when sedimentary quartz grains were last exposed to light prior to burial. Quartz grains were processed under safe light (dim red LED) conditions at the CENIEH Luminescence Dating Laboratory using standard preparation procedures [74], including a 48% hydrofluoric acid etch (40 minutes) to remove the alpha-irradiated outer layers of the quartz extracts. Single-grain OSL measurements were made using the experimental apparatus and procedures described by Arnold et al. [75, 76] and further detailed in the (Section SI-9 in S1 File). Between 2800 and 3400 single-grain equivalent dose (D_e_) measurements were made for each sample, with individual D_e_ values being included in the final age calculation if they satisfied a series of standard quality assurance criteria (Section SI-9 in S1 File). The environmental dose rates for the Aranbaltza II OSL samples were estimated using a combination of in situ field gamma spectrometry and low-level beta counting, taking into account cosmic ray contributions, an assumed minor internal alpha dose rate, beta-dose attenuation and long-term water content (Section SI-9 in S1 File).

Bayesian modeling was used to constrain the timing of Châtelperronian layers at individual sites across France and Iberia, and to derive a combined age estimate for the Châtelperronian culture across southwest Europe (Section SI-10 in S1 File). The modeling was undertaken using OxCal v4.4.3 [77], and Phase models were run using the general Outlier function [78]. To calculate the combined age range of the Châtelperronian layer(s) at each site, we have pooled all published radiometric age estimates that are methodologically reliable, are unaffected by post-depositional complications and have direct or indirect stratigraphic association with Châtelperronian layers, as a single, unordered Phase model with delimiting start and end boundaries. To constrain the timing of the Châtelperronian culture in southwest Europe as a whole, we have pooled the modeled durations of the Châtelperronian layers at individual sites (derived from the site-specific Phase models using the Date command) with the new OSL age obtained for the Aranbaltza Châtelperronian, as part of a single, unordered regional Châtelperronian Phase model.

### 4.6 Lithic analysis

Raw materials were classified according to the macroscopic petrographic features of the materials. The technological analysis was carried out through a techno-economic approach that integrates raw material procurement, production, management and use of lithic artifacts, considering also the available contextual information [42]. For the technological analysis we followed the chaîne opératoire concept [21, 79–81], identifying various stages in lithic tool making and investigating the basic conceptual processes which underlie the sequence of manufacturing steps in stone tool production. Retouched pieces were classified following the Sonneville-Bordes system [82–85].

## Supporting information

S1 File Supplementary information for the intrusive nature of the Châtelperronian in the Iberian Peninsula. SI-1. Discovery of Aranbaltza and earliest work; SI-2. 2013–2016 excavation project at Aranbaltza II; SI-3. Stratigraphy and site formation processes; SI-4. Spatial analysis; SI-5. Out.of-context Châtelperronian materials; SI.6 Lithic assemblage from Aranbaltza II US4b; SI-7. AMS 14Cdating; SI-8 Single-grain OSL dating experimental procedures and results; SI-9 Bayesian modelling of available ages for the Châtelperronian culture and associated hominin remains across France and the Iberian Peninsula; Supplementary S1-S39 Figs; Supplementary S1-S15 Tables; Supplementary References.
(PDF)
