# Supplementary material for: The intrusive nature of the Châtelperronian in the Iberian Peninsula
Source: PLoS One. 2022 Mar 30;17(3):e0265219. doi: 10.1371/journal.pone.0265219 (PMC8967055; doi:10.1371/journal.pone.0265219)
Supplement: S1 File — SI-1. Discovery of Aranbaltza and earliest work; SI-2. 2013–2016 excavation project at Aranbaltza II; SI-3. Stratigraphy and site formation processes; SI-4. Spatial analysis; SI-5. Out.of-context Châtelperronian materials; SI.6 Lithic assemblage from Aranbaltza II US4b; SI-7. AMS 14Cdating; SI-8 Single-grain OSL dating experimental procedures and results; SI-9 Bayesian modelling of available ages for the Châtelperronian culture and associated hominin remains across France and the Iberian Peninsula; Supplementary S1-S39 Figs; Supplementary S1-S15 Tables; Supplementary References. (PDF) [file pone.0265219.s001.pdf]

Supplementary Information for:

The intrusive nature of the Châtelperronian in the Iberian Peninsula

Joseba Rios-Garaizar, Eneko Iriarte, Lee J. Arnold, Laura Sánchez-Romero, Ana B. Marín-Arroyo, Aixa San Emeterio, Asier Gómez-Olivencia, Carlos Pérez-Garrido, Martina Demuro, Isidoro Campaña, Laurence Bourguignon, Alfonso Benito-Calvo, María J. Iriarte, Arantza Aranburu, Amaia Arranz-Otaegi, Diego Garate, María Silva, Christelle Lahaye, Illuminada Ortega

Corresponding author: Joseba Rios Garaizar
Email: jorios76@gmail.com

This PDF file includes:

Supplementary Text (SI-1 to SI-9)
Figs. S1 to S39
Tables S1 to S15

Supplementary Text

SI-1. Discovery of Aranbaltza and earliest work

Joseba Rios-Garaizar
Diego Garate
Illuminada Ortega

In the 1950s, several archaeologists and amateurs (A. Aguirre, M. Grande and S. Muñiz) recovered archaeological materials in the region known as Uribe Kosta (Biscay, northern Spain). Most of the materials were recovered by A. Aguirre in 1957 in two localities, Sopelana and Barrika. Some of them are currently held at the Arkeologi Museoa in Bilbao.

In 1959, J.M. Barandiarán visited this region to explore it, and the results were published in 1960 [18]. In this publication there are descriptions of the main stratigraphic sections identified in the Ollagorta E, Ollagorta W, Aspiribil and Iturralde sand deposits. At Ollagorta, two stratigraphic tests were carried out in the front of a sand quarry that was opened between 1946 and 1956, according to aerial photos obtained by the U.S. Army. We have personal communications recounting how A. Aguirre frequently passed by this quarry to collect the "artifacts" that appeared in the sieves. When Barandiarán conducted the stratigraphic tests, the quarry had already expanded down the Urgozo stream towards the place known as Aranbaltza, as we have inferred from the photograph published by Barandiarán et al. [18: Photo 3] and the aerial photo taken by the Diputación Foral de Bizkaia in 1965 (Figure S1). Barandiarán performed two tests: one on the east side and another on the west side.

> "*En el arenal de Ollagorta, así llamado por el nombre del caserío más próximo al lugar, nos proporcionó los primeros materiales arqueológicos, así como los datos que nos permiten vislumbrar su perspectiva cronológica.*
> *En la figura 2 presentamos el corte del lado E de dicho arenal en el que, bajo una capa de tierra vegetal reciente (D), existen otras dos de arena (C y B), siendo de un espesor de tres metros la más profunda, la cual se asienta sobre una base de arcilla compacta (A).*
> *En el lado W. del mismo arenal la serie de niveles es semejante a la del E., si bien existe un punto en el que el estrato C presenta un hoyo que ha sido colmado de residuos de hogar, indudablemente de fecha más reciente que la del estrato que lo engloba (fig. 3).*"[18: 12-13].

Initially we thought that both tests had been performed at Ollagorta. In 2019, we obtained a copy of the map drawn by hand by J.M. Barandiarán, that is currently held at the Barandiarán Foundation. In this map, which was cleaned up and simplified for the publication [18: Fig. 1], three excavation points were positioned, two clustered close to the Ollagorta farmhouse, and the other away to the east (Figure S3). In view of the scale and the positions of two farmhouses (Ollagorta and Goyeneuri), this eastern excavation

test was very likely at Aranbaltza, which was probably considered by Barandiarán to be the eastern side of the Ollagorta quarry.

Interestingly, level C from the eastern side (the one at Aranbaltza), is described as a reddish sandy deposit (US4?), over a blackish sandy deposit (US5?) with a clay deposit in the bottom (US6?) (Figure S4). This sequence corresponds roughly with the sequence identified by us at Aranbaltza II. In level C, he found several lithic tools including a backed blade that he considered Aurignacian [18]. After analysis of this collection, we identified a typical Châtelperronian point, an endscraper on cortical blade, a marginally backed blade, a crested blade and an overshot blade with previous bidirectional removals and an opposed platform, that could be interpreted as Châtelperronian [19]. Also, the assemblage of materials collected in 1957 by A. Aguirre at Barrika (most probably in the Ollagorta-Aranbaltza quarry), also includes some typical Châtelperronian points and marginally backed blades (Figure S5).

Considering all this, it is very likely that the eastern side of Ollagaorta was in fact Aranbaltza, and probably a place very close to Aranbaltza II, given the rough coincidence of both sequences and materials. Therefore, we must credit A. Aguirre and J.M. Barandiarán as the discoverers of the Aranbaltza site.

After the publication by Barandiarán, the materials from Ollagorta and other sites around Kurtzia were the object of scrutiny by several researchers. Francisco Jordá Cerdá considered that the backed points published by J.M. Barandiarán could be Gravettian [86]. Shortly after, M.C.R. McCollough analyzed the materials from the A. Aguirre, M. Grande and J.M. Barandarian collections and described the backed points as "*not "Gravette" types, but rather large convex-backed Châtelperronoid knives and points...the blanks are long and relatively very wide flake-blades with pronounced bulbs of percussion (often preserved, even on the proximal ends of points). The modal morphology is pronounced Large Châtelperronoid, and the blunting retouch on the convex backed edges is modally unidirectional and not very abrupt.*" [87: 220]. He even considers that, if these industries are actually Châtelperronian, it would be "*the result of population movement or stimulus/bow-wave diffusion (Clarke 1968:424) from France similar to the stimuli which had somewhat earlier given rise to short-lived and heterogeneous French "Châtelperronian" industries*" [87: 329].

The quarry of Ollagorta-Aranbaltza was exploited at least until 1965; the site was then used for dumping industrial debris, and then for a eucalyptus plantation (Figure S2). In the 1980s, some lithic materials were collected on the Aranbaltza surface by I. Libano and by J.A. Libano, but the site was never properly surveyed. Also in the 1980s, J.A. Libano recovered some materials from the final small quarry opened at Aranbaltzabarrena, including four Châtelperronian points (see SI-5).

In 2004 housebuilding started at Aterpeta, a new development located above Ollagorta and Aranbaltza. The construction of a sewer system for this block running parallel to the Urgozo and cutting through Ollagorta and Aranbaltza, revealed new archaeological deposits in Aranbaltza and Ollagorta. At this latter site, we recovered a

large Neolithic/Calcolithic lithic assemblage [88-89]. At Aranbaltza, I. Libano recovered, from the reworked sediments, a large lithic assemblage including 18 Châtelperronian points/knives, endscrapers on flakes, marginally backed blades, Châtelperronian blade cores, and many blades and blade production byproducts [19]. Around the same time, J.A. Libano also recovered a large lithic collection from the same reworked sediments. We accessed this collection in 2017 and recognized there a collection similar to that gathered by I. Libano, including 14 typical/atypical Châtelperronian points/knives, alongside other typical products. Both collections are now deposited at the Bizkaia Arkeologi Museoa in Bilbao (SI-5).

SI-2. 2013-2016 excavation project at Aranbaltza II

Joseba Rios-Garaizar
Eneko Iriarte
Aixa San Emeterio
Diego Garate
María José Iriarte
Illuminada Ortega

In spring 2013, we surveyed the area of Aranbaltza and found an *in situ* stratigraphic sequence in a 2 m² test pit. Then, in summer 2013, we undertook the first archaeological excavation at the site. To identify where the archaeological sites were, we dug test trenches with excavation machinery which was stopped once an archeological level was found. Trench 2 gave positive results immediately after the recent humic layer was lifted, and then we started manual excavation in an area of 7 m² (Area 1) until the Châtelperronian deposit (US4b) was reached and excavated (Figure S6, S7). From this moment this site was denominated Aranbaltza II. In total, excavations at the Aranbaltza site complex have identified three archaeological sites (Aranbaltza I, II and III) (Figure 2) with comparable archaeo-sedimentary sequences spanning from the Late Middle Pleistocene all the way to the Holocene [20, 33].

In 2014 we cleared a large surface around Aranbaltza II using machinery, and found the sewage trench to the south of the 2013 excavation block. We then started the manual excavation of 4 m² at the southern side of the sewage trench, finding there the remnants of the Châtelperronian occupation, that have been severely altered by post-depositional processes (Area 2). Also in 2014, we extended the excavation a further 7.14 m² to the west of the 2013 excavation block (Area 3). In 2015 we continued excavating this area until 2016 when we reached and excavated the Châtelperronian unit (US4b). Finally, in 2015 and 2016 we made two manual pits to test the extension of the Châtelperronian occupation, one to the W of Area 2, between Aranbaltza I and Aranbaltza II, and the other to the N between Aranbaltza II and the Trench 1. Both tests were negative.

The area excavated in 2014, situated to the south of the sewage trench, was very altered by the excavation of the trench and by the eucalyptus planting. A 70 cm deep deposit of reworked sediments was excavated, with many mixed lithic tools and some ceramic fragments, mostly concentrated on the base (Figures S8 and S9). Among these reworked materials, 3 Châtelperronian points were found. Once the reworked sediment was excavated in some parts of the 4 m2 excavated surface, in situ hardened sandy orange sediment was reached, and some lithic tools were found in it, including cores and a light Châtelperronian point (Figure S9).

In 2016, US4b was reached in Area 3. A hardened, orange, sandy deposit with abundant lithic materials was revealed and excavated (Figure S10). The contact with the US4a was clearly defined to avoid any possible contamination. The archaeological materials appear on a N-S inclined surface, with a maximum thickness of 10 cm, and

below it US4c is archaeologically sterile. This unit was cut by the sewage trench. The edge of this trench was clearly defined in the S section of Area 3.

The entire extension of US4b was excavated following the open area system. The archaeostratigraphic units, defined by changes in the sediments, were excavated in extension. All the archaeological remains larger than 1 cm were coordinated using a topographic total station, and situated in an artificial coordinate system adapted to the nature of the site. Smaller finds recovered from irregular areas of c. 25 cm diameter were grouped into 'lithic' bags, and a coordinate was assigned at the center of these irregular areas. In addition, the sediment recovered from these irregular areas was kept, and its spatial position was recorded with a single coordinate taken in the center of the excavated area. In 2016, the orientation and inclination of the elongated materials bigger than 2 cm was also recorded. The sediment the from 2013 Area 1 and 2014 Area 2 excavations was dry sieved with a 1 mm mesh. The sediment from the 2016 Area 3 excavation was water sieved with a 1 mm mesh.

Two pollen samples were recovered from the eastern section of the 2016 excavation block. Unfortunately, the preservation of pollen is poor and the combined sample does not reach the threshold of 250 specimens required to consider it statistically meaningful. Despite this, the arboreal taxa identified (*Quercus robur tp.*, *Corylus* and *Fagus*) and the abundance of fern spores, Liliaceae and Juncaceae, points to mild environmental conditions (mild temperatures and greater humidity). Similar records have been obtained from the Châtelperronian level of Labeko Koba (level IX lower: 42.610 – 41.450 cal BP), where arboreal taxa like *Corylus*, *Quercus robur tp.* and *Castanea* are present [137].

SI-3. Stratigraphy and site formation processes

Eneko Iriarte
Arantza Aranburu
Isidoro Campaña

Basic geological description

The Aranbaltza archaeological site complex is located on Upper Cretaceous (Albian to Turonian) carbonate flysch with interspersed diabasic volcanic intrusions (sills and dikes) (Figure S11). The Upper Cretaceous materials are part of the northern limb of the Biscay Synclinorium, and their bedding is, in general, dipping gently (70º) towards the SW, but is very variable due to intense folding and faulting.

From the geomorphological point of view the Upper Cretaceous rocks form a Plio-Quaternary marine terrace elevated at ca. 90 m above the modern main sea level. In this terrace, the Urgozo stream excavated a 2 km long fluvial valley flowing towards the Butrón river valley (nowadays an estuary) to the SSE (Fig. 2). The fluvial incision was helped by the presence of a previous erosive paleorelief with Plio-Quaternary marine/transitional sandy sediment infillings above the marine terrace.

Materials and methods

Sedimentology and coring: A 2 m depth core was collected using a Van Walt/ Eijkelkamp window corer, which permits the recuperation of the sedimentary records by accumulating 1 m-depth operations. Each core was replicated to ensure they were representative. Once collected, the samples were sealed and stored at 3–4 °C. The two-meter depth AAR4 core, obtained near the southern profile of the excavation area (1 m to the south), was selected as representative of the complete stratigraphy observed in the profiles of the excavation pit. The sedimentological study consisted of the stratigraphical characterization and the description of the sedimentary facies observed in both the excavation profiles and in the AAR4 core (Figure S12).

Granulometry and grain morphology analysis: The cores were split into two halves and imaged with a high-resolution digital camera in an XRF core scanner. One half of the core was sampled every 10 cm for XRD and particle size and texture analysis.

The texture of the samples was analyzed using particle size sieving and laser diffraction techniques. For sieving techniques, a φ size sieve, range –3 φ to 4 φ, was used. A Beckman Coulter LS13 320 laser diffraction particle size analyzer was used to measure the particle size in the silt and clay fraction. Particle size was classified following the scheme of Blott and Pye [70]. The morphological analysis of sand particles was performed using a Malvern Morphologi G3 particle characterization system (Malvern Instruments, Malvern, UK). For this purpose, the grains in the sample were separated and dispersed over a glass plate by air injection using a Sample Dispersion Unit (SDU). Next, the instrument took high resolution gray-scale images of the complete glass plate using a motorized slide. The particles were identified using a gray-scale threshold and then the

shape parameters were calculated. The analyses were conducted following the procedures described by Campaña et al. [71]. After each analysis, post-processing of the data was performed. This post-processing consisted of the elimination of joined particles, non-minerals (i.e. organic matter, ambient dust) and poorly identified particles. Several size and shape parameters were measured for each grain. The shape parameters used were aspect ratio, high sensitivity circularity and convexity. The aspect ratio is the ratio of particle width to length, and ranges from 0 to 1. High sensitivity circularity (HSC) indicates the similarity of a particle to a circle and the values range from 0 (extremely narrow rod) to near 1 (perfect circle). Finally, convexity is calculated from the ratio between the particle perimeter and the perimeter of its convex hull. This shape parameter indicates the roughness of the particle [71-72] and its value ranges from 0 (extremely rough) to 1 (extremely polished).

Mineralogical analysis: X-ray Diffraction (XRD): Bulk sample mineralogy was determined by powder X-ray diffraction (XRD) using a Bruker D8 Discover DAVINCI diffractrometer at the Science and Technology Park, Burgos University. Air-dried samples were sieved at 2 mm, finely ground in an agate mortar, and processed using a continuous scan range of 2º-80º 2θ, a 0.05º step size and 1 second time per step. The sample was irradiated with Cu Kα radiation (ceramic X-ray tube KFL-Cu, 40 kV, 40 mA) with a programmable divergence slit, and a LynxEye detector was employed. Semi-quantitative estimations were calculated from peak areas on XRD patterns using DIFFRACplus basic EVA software with ICDD database.

Stratigraphy and sedimentology of Aranbaltza II site

The description of the sedimentary sequence was compiled based on the information provided by the profiles of the archaeological excavation pit and from the two-meter long AAR4 core, which shows a similar stratigraphic succession. Overall, the excavated stratigraphic record is 2 m in depth and different sedimentary facies and stratigraphic units are visible (Figure S12). The lithostratigraphic units were differentiated by their distinct sedimentary facies [90].

Six lithostratigraphic units (US0 to US5) and 3 main sedimentary facies were defined, with the Châtelperronian occupation included Stratigraphic Unit 4 (US4), which includes 3 different subunits (US4a, US4b and US4c).

Unit 0: This surface unit is discontinuously present and fills decimeter- to meter-scale depressions. It is composed of mixed angular heterometric (cm to decimeter-scale) fragments of clay and sandy sediment (Table S1). The sediments identified in the clasts derive from underlying stratigraphic units. This unit is interpreted as an accumulation of reworked sediments due to different anthropic excavation activities related to a former sand quarry.

Unit 1: This is composed of an organic matter-rich 7 cm thick quartz silty sand layer. The mineralogical composition of the sediments indicates a major content in quartz

grains (78.8%), clay minerals (11.8%) and feldspars (8%), and high organic C (Corg) content (5.3%) (Table S1 & Fig. S12). It includes numerous roots and plant remains, centimeter-scale anthropic rubble and sediment lithoclasts from underlying units. The lower contact is sharp.

This unit is interpreted as the surficial soil, Ah horizon, mixed with reworked sediments and anthropic materials related to the former sand quarry workings.

Unit 2: This unit comprises medium to coarse massive to slightly laminated quartz sand, Sh facies [90]. No sedimentary structures are visible and its thickness is laterally highly variable, from 10 cm to 40 cm. The sands are orange-stained and include abundant black mottles, from root bioturbation marks filled with mineralized organic matter. The sands are composed of quartz (83.7%), feldspars (7.9%) and clay minerals (7%), with 1.9% Corg (Table S1 & Figure S12). The basal contact is very irregular and gradual.

This unit is interpreted as corresponding to the distal part of different aggrading tractive sandy sediment sheets. The broad and shallow geometry of the lithosome suggests a crevasse splay origin for these sediments. The post-sedimentation orange and black staining by abundant root marks is noteworthy, suggesting the intermittent presence of vegetated paleosoils and podzolization processes forming the Ae and Bs horizons. Overall, it could be considered as an aggrading fluvial paleosoil (podzol) sequence developed upon superimposed crevasse splay sand sheets in a floodplain near a fluvial sandy channel.

Unit 3: This is composed of medium massive to slightly laminated gray, clayey quartz sand, Sm facies [90]. No sedimentary structures are visible and the grain size is very well-sorted. Its thickness is approximately 1 m. The sand mineralogy shows quartz (81%), feldspar (9.3%) and clay mineral (8.1%) grains, with a low Corg content (1.3%) (Table S1 & Fig. S12). The base of unit 3 displays gradual transition to Unit 4.

This unit is interpreted as an incised sandy channel fill consisting of multiple sandy high-density flowing events. The post-sedimentation soil activity is noteworthy, and the pale gray color suggests intense eluviation.

Unit 4: This unit comprises three subunits: US4a, US4b and US4c:

US4a is the youngest subunit of Unit 4. It is nearly 30 centimeters thick and is composed of the same gray clayey sands as Unit 3, Sm facies. In this case, however, it presents some intervals with black mottling (mineralized root bioturbations) and an irregular subtle yellowish to orange staining of the sediments. The mineralogy of the sands show a major content in quartz grains (83.4%), feldspars (7.4%) and clay minerals (7 %), while the Corg content is (1.3%) (Table S1 & Figure S12). Its top shows gradual transition with Unit 3, and the basal contact is highly erosive, creating a decimeter- to meter-scale erosive surface (S3) dipping towards the south. Sparse centimeter-scale granules are visible in the bottom of the unit.

US4b lithostratigraphic unit encompasses the most significant archaeological content studied in the present article. It is composed of medium very well sorted massive sand without visible sedimentary structures. The sands at the top of the subunit are massive and yellowish, but below they are orange-stained and include abundant black mottles, of root bioturbation marks filled with mineralized organic matter. The sand mineralogy shows the highest content in quartz grains (92.5%), feldspars (5.5%), and clay minerals are nearly absent (1%), with the lowest Corg content (0.3%) (Table S1 & Figure S12). The maximum observed thickness of the unit is approx. 25 cm. due to the upper erosive contact, S3, that locally has even eroded the entire S4b subunit. The bottom of US4b is formed by another erosive contact, S2, which also presents decimeter-scale erosive features that form a southward dipping surface that amalgamates with the S3 erosive surface towards the north.

US4c is very similar to US4b: it is a 30 centimeter-thick orange-stained sandy unit. In the top 10 centimeters, it is massive and yellowish, and towards the bottom the sands are orange-stained and include abundant black mottles and root bioturbation marks filled with mineralized organic matter. The transition between both facies is marked by the presence of an iron oxide nodule-rich layer. The sand mineralogy presents a high quartz content (90.4%), feldspars (4.8%) and very low clay mineral presence (2.9%), while the Corg content is just 0.7% (Table S1 & Figure S12). The bottom corresponds to an erosive surface (S1) that has eroded the underlaying black organic-rich silty sands from Unit 5.

Unit 4 is interpreted as a group of 3 different sand accumulation intervals (US4a, b & c subunits) related to enhanced flooding activity in the nearby river channel. The sand lithosomes corresponding to the subunits are bounded by erosion surfaces (S1, S2 and S3) that comprise long-lasting erosive and/or depositional hiatuses associated to colonization by plants (root bioturbation) and soil formation (podzolization).

US4c shows an intense orange hue and iron oxide nodules typical of Fe and Al illuviation and precipitation (Bs & Bx horizons) in podzols. Its upper eluvial horizon (Bs) is partially eroded by the S2 erosive surface and its lower contact corresponds to the S1 erosive surface that erodes the swampy organic sands from Unit 5. Thus, after deposition a podzol-type vegetated paleosoil developed for a long time upon US4a during the hiatus represented by the S1 surface.

US4b is similar to US4c, and presents the same depositional and pedogenic features. The only significant difference is its abundant archaeological content, characterized by the presence of plentiful knapped flint remains. The sands have an intense orange hue and mineralized bioturbations typical of Fe and Al illuviation in podzols (Bs horizon). Its upper eluvial horizon (Bs) is partially eroded by the S3 erosive surface and the lower contact is the S1 erosive surface. Thus, again a podzol type vegetated paleosoil developed for a long time upon US4b during the hiatus represented by the S2 surface.

US4a is similar to previous subunits, and is formed of aggrading gray clayey sands that were colonized by vegetation (root bioturbations) and slightly pedogenized (yellowish hue). This time, the aggradation was not interrupted by an exposure/erosive surface, the sedimentary aggradation continued and massive sand channel facies from Unit 3 covered the US4a floodplain (crevasse splay) sediments. It is noteworthy that Unit 3 also contains sparse knapped flint remains.

Unit 5: This unit consists of massive black organic silty sands (5a and 5c subunits), SS facies [90]. The mineralogy of Unit 5 shows a high quartz content (88.9%), feldspars (5%) and clay minerals (4.5%), and the Corg content is relatively high (2%) (Table S1 & Fig. S12). A minimum thickness of 30 cm was observed in the excavation trench, since the unit was not fully excavated. The S1 erosive surface constitutes its upper contact and forms a southward dipping surface.

The sediments of Unit 5 are interpreted to have been deposited in a vegetated backswamp area where fine-grained (clay and silt) deposits and sporadically distal crevasse splay sandy sediment accumulated (crevasse channels or lobules) during flood events.

Concerning grain size and texture parameters for the sediments, the similar results obtained from all the stratigraphical units are of note. The grain size is similar in all the units, corresponding to fine sand (0.125-0.250 mm) (89 to 33%) with variable amounts of silt (5 to 39%) and clay (5 to 22%) (Table S1 & Figure S12). Unit 4 is the most sandy unit (90 to 75%). The results of the three shape parameters obtained (HSC, Convexity and Aspect ratio, Table S1) show very similar values without significant variation. This indicates a homogeneous character for the sediments along the sedimentary record studied, without significant changes in sediment transport or in the sediment source occurring. The largest size fraction, sand, has the highest values, and is the best particle size for detecting changes to grain shape caused by different sedimentary processes [71]. The high values of convexity have been related to aeolian transport [71], suggesting that the source of the Aranbaltza site was earlier aeolian sediments. This aeolian transport occurred before the fluvial transport that formed the site, pointing to the source of the sediments as the Plio-Pleistocene aeolian sand unit outcropping elsewhere on the Urgozo valley slopes and nearby coastal areas [91]. Subsequent fluvial modifications to the shape of the sand grains were not observed, due to the high maturity of the sediments and the brief, low-intensity fluvial transport that was undergone.

Sedimentary environments and site formation processes

The archaeologically significant Unit 4 from Aranbaltza II site is interpreted as crevasse splay sediments deposited in floodplain environments. A floodplain is the strip of land that borders a river channel, and it is normally inundated during seasonal flooding. Floodplains contain active and abandoned channels and bars (the channel belt), levees and crevasse channels and splays. Levees are discontinuous, wedge-shaped ridges around active and abandoned channels. Levees commonly have channels cut into their surfaces. The larger ones are called crevasse channels and split downslope into smaller

distributaries surmounting fan- or lobe-shaped mounds of sediment called crevasse splays (Unit 4). Insofar as crevasse channels operate only during floods, they are ephemeral channels. Permanent marshes (Unit 5) may be present in floodplains in wet climates.

Sediment is transported over floodplain as bed load and suspended load during overbank floods. The sources of sediment are the main channel and tributary channels, the valley sides and the floodplain itself. Although the grain size of sand on floodplain surfaces tends to decrease away from the active channel belt, mud usually accumulates as a more or less continuous blanket, mainly in distal swampy areas or marshes. Erosion occurs where flow is accelerated in locally narrow or topographically high floodplain sections and where plant cover is poor, forming erosive surfaces like those observed at Aranbaltza II (S1, S2 and S3 surfaces).

The basic sedimentation units in floodplains from Unit 4 would be millimeter- to decimeter-thick stratasets deposited during overbank flooding events. As observed at Aranbaltza II, basal erosion surfaces are present if erosion preceded deposition. Grain sizes and internal structures depend on local flow conditions and sediment availability, in this case comprising very mature aeolian fine sand and fine sediments from fossil Plio-Pleistocene sandstones. The upper parts of these stratasets are commonly bioturbated with root casts. Layers of drifted vegetation are common in the overbank deposits of humid climates.

Flood-generated stratasets of crevasse splays are similar to those of levees, but channel-bar and channel-fill deposits as inferred for Unit 4 are common in crevasse splays, and these may be difficult to distinguish from the main channel deposits. For Aranbaltza II, our interpretation is that Unit 4 was formed as a crevasse splay related to a crevasse channel that episodically eroded previous floodplain sand and marshy facies from Unit 4 itself and Unit 5.

In essence, Unit 4 comprises 3 subunits corresponding to floodplain sandy sediments deposited in crevasse splay environments due to overbank floods during enhanced flooding intervals of a nearby river channel. The unit comprises an aggrading sequence of fluvial paleosoils (podzols) developed after flood sediment deposition. The sandy sediments formed a subaerially exposed and vegetated floodplain area near a fluvial channel and likely swampy areas (e.g. Unit 5) that were frequented by humans that left lithic remains during the formation of US4b.

The information derived from spatial analysis of the archaeological lithic remains from the US4b subunit is characterized by the occurrence of a dense blanket of non-size selected lithic remains with some clustered accumulations but without preferential orientations. This fact may point to minor flooding events that slightly reworked the lithic accumulation area. During low-energy flood events, water would erode and transport sand, sweeping sandy and fine sediment and forming sand-poor local accumulations of lithic remains nearly in situ in the areas of highest water flow.

SI-4. Spatial analysis

Laura Sánchez-Romero
Alfonso Benito-Calvo

The spatial study of Aranbaltza II was carried out considering all the materials excavated during the fieldwork undertaken between 2013 and 2016, but we have concentrated on Areas 1 and 3 because they were better preserved, and they encompassed the bulk of the archaeological evidence for the Châtelperronian occupations. The database compiled comprises all the coordinated and non-coordinated materials. A random position on the 30 cm diameter area around the sediment bag of provenance (see above) was assigned to every uncoordinated element. Thus, for the spatial analysis, we have considered all the excavated lithic artifacts, not only those with coordinates, thus increasing the sample size to 5414 remains.

The kernel density analyses [92] were performed with a search radius of 0.30 cm, due the size of the site and the fact that the analyzed materials are represented by points [93-94]. The result obtained shows a clear accumulation of material at the west of the main excavated area (Area 3) (Figure S13), which coincides with the presence of the sedimentary structures previously described in the geology section. Despite the fact that the main concentration of remains is in this zone of Area 3, it is remarkable that the rest of the area and the western part of Area 1 also contain an important distribution of materials, although the concentration is less significant. The remains clustered in the western zone of Area 3 comprise 33% of the whole assemblage. These concentrations do not show any size or shape selection and the preservation of the lithic materials is good, with only a bright patina originated by the contact with sand being apparent.

The refitting distribution pattern has also been analyzed, with the aim of identifying the existence of specific spatial characteristics that could point to some specific pattern. Seven refitting sets have been identified so far, and they are dispersed along the whole excavated area, encompassing and connecting the three areas of Aranbaltza II, although their density rates differ greatly (SI-6). The refitting lines are, in general terms (Fig. S12), long, the longest reaching 4.91 m, for refitting set number 6. These distances point to an important displacement of the pieces, especially considering the size of the site. However, and due to the fact that this is a small sample, analysis of the orientation patterns of the refitting lines could not be performed, since the results would not be significant. This interpretation of the spatial distribution of the remains is limited by the destruction caused by the sewage trench excavation. In fact, considering that a huge assemblage was recovered from the reworked sediments in this area (SI-5), and observing that the main concentration of artifacts is situated just next to this sewage trench, we can infer that the area under discussion was part of a bigger concentration. The striking absence of materials in the eastern part of Area 1 and the absence of materials in the two test pits excavated at the northwestern part of Area 3 and at the west of Areas 2 and 3, suggest that the Châtelperronian occupations of Aranbaltza II were preserved in a discrete subcircular concentration of materials (<4 m diameter), similar to those described for other open-air Châtelperronian sites, such as Canaule II, Vieux Coutets or Ommersson [22, 24, 29]. Unfortunately, the preservation problems suffered at Aranbaltza

II prevent saying any more about the spatial structure of these Châtelperronian occupations.

SI-5. Out.of-context Châtelperronian materials

Joseba Rios-Garaizar
Aixa San Emeterio
Illuminada Ortega

Materials recovered from the sewage trench.

The materials presented here were recovered from the dumped sediments excavated to build a sewage trench in 2004 (Figure S15). Two sets of artifacts were collected independently by I. Libano and J.A. Libano in the vicinity of Aranbaltza II. The I. Libano collection was studied in 2011 and presented by some of us in 2012 [19]. The collection of J.A. Libano was studied between 2017 and 2018 and is going to be presented here for the first time. This collection includes also some materials from the Aranbaltzabarrena site, some of them, including 5 Châtelperronian points and 2 backed blades, having been separated as appertaining to that site, but the rest of the material, not very numerous, was kept together with Aranbaltza materials and was impossible to distinguish the sites. Both collections are currently held by the Arkeologi Museoa (Bilbao).

These materials have no direct contextual information and are surely mixed with Mesolithic and Calcolithic materials, as was already noted in 2012 [19]. Interestingly, and considering that close to Aranbaltza II there are two important Middle Paleolithic sites, Aranbaltza I and Aranbaltza III [20, 33], there are not many Middle Paleolithic materials in either of the collections. This matches what has been observed from in situ recovered materials from US4b. We must also clearly say that both collections are heavily biased, with small artifacts, byproducts and chunks poorly-represented, probably because they were not so visible or interesting for non-professional archaeologists. We present here these two collections merged (for particular details on the I. Libano collection see [19]) once the clearly post-Paleolithic artifacts (i.e., polished axes, pressure blade cores, etc.) have been purged.

The assemblage is large, even considering that a part of it could correspond to Mesolithic or post-Paleolithic occupations. It is clearly dominated by blades, blade cores, and blade core configuration and maintenance products (Table S2). Also, probably a great number of the cortical flakes were produced in the initial stages of blade core configuration. Regular flakes, and also all the tool resharpening products, are poorly represented, and there are also only a few flake cores, including Levallois and Discoid cores.

Among the cores there is the same variety as observed in the in situ archaeological assemblage (Table S3). Most of the cores are unidirectional, some of them with quadrangular cross-sections for the production of asymmetrical blades typical of sites like Quinçay, Grotte du Renne, Roc-de-Combe and La Côte [21, 25-26]. Some of these cores were abandoned after the removal of elongated flakes from the flanks. There are typical maintenance products from these production activities, for example, the one-sided crested blades, or the blade core rejuvenation flakes. Usually these cores are

abandoned after accidents such as a hinged blade. There are also a few bidirectional cores with not strictly parallel flaking surfaces *(bipolaire décalé*) for the production of pointed asymmetrical blades, that match some productions identified at sites like Vieux Coutets, Roc-de-Combe, Le Basté or Canaule II [21-24], and typical maintenance products from these productions are one-sided crested blades, overshot blades, usually dragging on opposed platform, and platform rejuvenation flakes. Normally, these cores are abandoned when the available volume is not enough to continue the exploitation, and there are some examples of exhausted cores. Finally there are also a few, non-diagnostic, unidirectional prismatic bladelet cores similar to those recovered from in situ collection. Among the blade products there are regular unidirectional blades, asymmetric unidirectional blades, asymmetric bidirectional blades, and pointed bidirectional blades. Usually platforms are not prepared, but many blades exhibit small detachments on the proximal part of the dorsal surface that account for the suppression of the ridge between the platform and the dorsal surface.

Retouched tools are varied and include a whole array of typical Upper Paleolithic tools plus a few typical Middle Paleolithic ones (Table S4). Among the most interesting ones are the endscrapers on flakes (n= 28), the Châtelperronian points (n=29 including typical and atypical), and the backed blades (n= 124), including some Châtelperronian-like knives, partially backed blades, typical backed blades and blades with marginal back, typical of the Châtelperronian [25].

Surface material

A lot of materials have been recovered from the surface of the Aranbaltza site between 2013 and 2019. Among these materials, a few can be attributed to the Châtelperronian, including 3 Châtelperronian points, a few backed blades, and some endscrapers on flakes (Figure S21).

Aranbaltzabarrena

In the vicinity of Aranbaltza, some materials were recovered from the sandy sediments removed during the building of a house in the place called Aranbaltzabarrena. As we said previously, some materials from this site were kept alongside with Aranbaltza materials, but some distinctive pieces, such as the Châtelperronian points, were kept aside. The assemblage separated by J.A. Libano as coming from Aranbaltzabarrena is formed pf 11 pieces, including 4 typical Châtelperronian points, 1 atypical Châtelperronian point, two backed blades, three one-sided crested blades and a flank blade (Figure S22). This material indicates a different Châtelperronian site in the vicinity of Aranbaltza II, but most probably this has been irremediably altered. In any case it would be on private property and access has not been granted.

SI.6 Lithic assemblage from Aranbaltza II US4b

Joseba Rios-Garaizar
Aixa San Emeterio
Laurence Bourguignon
María Silva
Illuminada Ortega

The lithic assemblage from Aranbaltza II US4b is composed of 5686 remains, basically made on flint (96.3%). The material is apparently well-preserved, without signs of transport, size-sorting or major chemical alterations. Despite this good state of preservation, the direct contact with the sandy sediment has generated a bright patina which is more noticeable at the microscopic level. This alteration has obliterated or masked the use-wear traces, preventing any traceological analysis. The clear definition of US4b precludes any contamination due to excavation. Though we cannot completely exclude the potential for minor contamination between US4a and US4b due to root action, any such localised mixing would only minimally bias the characterization of the US4b lithic assemblage. In fact, US4a rather than US4b appears to be more influenced by such processes, resulting in the admixture of Mesolithic and Châtelperronian artifacts [20] (see Fig. 6 in 20)..

Refittings

In flint, there are two refitting series of flint slabs from Area 3. One (#401347, #401328, #400283) is composed of three fragments of a thin flint slab (17 mm) and shows a typical star-like fracture revealing that on-anvil bipolar percussion was used. The biggest fragment shows an assay at making a unifacial crest and probably it was intended to be a core support, but it was abandoned. The other large fragment has also a single extraction, possibly the result of a test (Figure S23: 2).

The other refitting on flint (#401047, #400366) is a core roughout made on a thicker (25 mm) flint slab. It is formed by a series of elongated supports with enveloping cortex that were obtained from a non-prepared platform. The last one of these elongated flakes (#400366) is fragmented due to an internal joint fracture of the slab. After this, or in parallel, a single extraction was made on the core roughout, probably for preparing a platform. After this, it was abandoned (Figure S23: 1).

There is also a refitting of a multipolar flake core from Area 2 US4b (#40025), with a partially cortical flake from Area 2 recovered in disturbed sediments (#32434); a refitting between a proximal Châtelperronian point from Area 1 (#4032, Figure S23: 3) with a medial blade fragment from Area 2 (#40043); and a refitting between a proximal fragment of Châtelperronian point from Area 2 reworked sediments (#32668, Figure S23: 4) with a proximal fragment of marginally backed blade from Area 1 (#4011, Figure S3: 5).

In sandstone there are three refitting series. Two of them, involving 6 fragments in total, probably belong to a single big slab (thickness c. 30 mm) (Figure S24: b, c). This

slab had straight edges shaped by on-anvil percussion. Later the slab was broken into several fragments by on-anvil percussion that prompted a typical star-like, radial fracture, producing triangular fragments with straight sides, opposed percussion bulbs and sinuous central ridges. The other refitting series involves a series of three flakes (Figure S24: a) including one fragmented flake from Areas 1-3 US4b (proximal fragment #4090, and distal fragment #401345), one flake recovered from Area 1 US3 (#1535), and a later flake from Area 3 US4b (#400324).

Raw material

The most widely used raw material (94.58%) is Upper Cretaceous Flysch flint [95], which is very abundant in the vicinity, the best-known sources being those located in the cliff of Kurtzia, 1.2 km away from Aranbaltza II. However, geological strata with flint, or more precisely marls and sandy-limestones containing flint, run closer to the site (c. 500 m). The external aspect of this flint is highly variable: from a petrographic point of view it is fine-grained, with a little chalcedony and opaline cementation, detritic quartz, and occasionally quartz grains and altered fragments of volcanic rocks. The fossil content is dominated by abundant silicified sponge spicules, radiolarians, fragments of foraminifera and, to a lesser extent, other bioclasts like orbitolines, gastropods, algae, etc [95]. Among the Flysch flint found at Aranbaltza II we have not recorded any fragment with evidence of marine abrasion, suggesting that the blocks of raw material were not recovered from the sea-battered cliff surface. There is a huge variety of Flysch flint types used at Aranbaltza II: we have recorded very good quality flint with sandy orange cortex and almost no internal fractures, other varieties of lower quality that are heavily fractured and do not allow controlled knapping, and fragments with a high content in silicified sponge spicules (Figure S25: a) and even silcretes (Figure S25: b). Colors vary from black to creamy brown, depending on the degree of patina. We have also documented the use of naturally broken fragments as supports for tools.

Other varieties of flint (1.69%) documented at Aranbaltza are pending a precise characterization although, among others, we have detected a wackestone flint with very good grain sorting (Figure S25:c); flint with miliolids, which is perhaps Piloña flint (c. 190 km W) [96] (Figure S25: d); flint with external marine platform fossils (Figure S25: e-f); internal marine platform translucent flint (Loza, c. 85 km S, or Monte Picota, c. 80 km W) varieties [96] (Figure S25: g); and Upper Cretaceous pelagic flint with bioturbations, probably of the Salies-de-Béarn (c. 165 km E) variety (Figure S25: h).

Other raw materials like sandstone (2.69%), trachyte (0.35%) or marl (0.02%) have been collected in the surroundings of the site, with only sandstone evincing a certain relevance in the toolkit used in Aranbaltza II US4b, since it was used to produce flakes, while the others have been used only as anvils or hammerstones. There are also small numbers of quartzite (0.32%), quartz (0.37%), ophite (0.02%) and fine-grained siliceous mudstone (0.04%) pieces. It is likely that these materials were collected in the Upper Cretaceous conglomerates that are nearby (<6 km N-NW). It is interesting to note the presence of ochre lumps (0.53%), probably also recovered close by, since iron ore

nodules are present in the megabreccias and in the sandstones and sandy-limestones around the site.

Technological features

A great part of the assemblage (60.4%) is composed of natural blocks and fragments, chunks, thermal flakes and chips. This reflects intensive block testing, core flaking and tool production. Some flint blocks could represent raw material reserves, but the majority are small fragments naturally transported to the site; the chunks were generated during core exploitation; the thermal flakes indicate the presence of fire somewhere close to the excavated areas. The chips are waste generated in core configuration, during knapping and during the configuration and use of tools, while some of them were probably generated during Châtelperronian point configuration [28].

Without considering these remains, the flint assemblage (n=2218) is dominated by cortical flakes and blades (24.8%) generated in the initial stages of core configuration. Cores are not very abundant (1.7%), in contrast with the composition of the collections recovered from reworked sediments (5.37%) (SI-5), but in numbers similar to other open-air sites like Vieux Coutets (1.71%) [24]. Most of the cores have produced bladelets (n=10) and blades (n=7), while there are also some non-standardized “expedient” flake cores (n=4) and some core roughouts (n=6), tested blocks (n=3) and exhausted cores (n=1).

Blade cores are bidirectional (n=4) and unidirectional (n=3) prismatic cores. The bidirectional cores present two opposed flaking surfaces that are not strictly parallel (*bipolaire décalé*) for the production of pointed asymmetrical blades (Figure S26: 1), that match some productions identified at sites like Vieux Coutets, Roc-de-Combe, Le Basté and Canaule II [21-24]. Typical maintenance products from these productions are the one-sided crested blades, the overshot blades, usually dragging on opposed platform, and the platform rejuvenation flakes (Figure S27). These cores are normally abandoned when the available volume is insufficient to continue the exploitation, with some examples of exhausted cores being found. Unidirectional cores have quadrangular cross-sections for the production of asymmetrical blades typical of sites like Quinçay, Grotte du Renne, Roc-de-Combe and La Côte [21, 25-26]. Usually these cores retain one cortical side and this explains the relative abundance of cortical flank blades (3.7%) (Figure S26: 2). All of these cores have been abandoned after a final hinged blade extraction. There are typical maintenance products from these productions, such as the one-sided crested blades, flank blades and blade core rejuvenation flakes.

All the bladelet cores are unidirectional (Figure S28, S29). Five of them are on flake edges and can be described as nucleiform burin (Figure S29: 1, 2) two of them are also carinated and have produced narrow (c. 4 mm) and slightly curved bladelets. The other five bladelet cores have been made on blocks (Figure S29: 3,4 and 6). One of them, recycled from a blade core, has two independent slightly curved flaking surfaces for the production of narrow (c. 4 mm) and slightly curved bladelets (Figure 28: c). This bladelet production has similarities with the bladelet production described at Quinçay [26]. The

most abundant maintenance product related to bladelet production is one-sided crested blades.

Blade core maintenance products are quite abundant (13.1%), including hinged blades (2.7%), probably resulting from poor preparation of the core; flank blades which are often cortical (4.3%); overshot blades, most of which were obtained from bidirectional cores (0.9%); single-crested blades (2.3%); double-crested blades (0.1%); platform rejuvenation flakes (1.3%); and blade core rejuvenation flakes (1.5%). If to this category we add the cortical flakes and blades and a large proportion of the blanks classified as flakes, which are also probably blade core maintenance products, the Aranbaltza II assemblage would be clearly dominated by blade production byproducts.

Flake cores are scarce (n=4 on flint): two of them have produced unidirectional and elongated flakes, and one of these could be an exhausted blade core. The other two have produced short unidirectional flakes and are rather opportunistic and not standardized.

Blanks are dominated by blades (26.7%). We have identified three classes of blades according to their width, probably corresponding to different productions. The most abundant are the narrow blades (51.91%) between 6.4 and 13.5 mm, which makes up the principal blade production at the site. These narrow blades exhibit regular parallel edges, unidirectional or bidirectional negatives, sometimes with pointed ends and thin flat platforms, frequently with platform ridge abrasion. Wide blades (>13.6 mm) are rather abundant (27.83%), and these are probably the result of an independent production, but were also occasionally manufactured from the narrow blade cores. The small bladelet (<6.4 mm) proportion is also important (20.25%): this corresponds mostly to an independent production from bladelet cores on flakes, but some of them were probably also produced unintentionally from narrow blade cores.

Flakes are rather abundant (15.1%) and have mostly been obtained from unidirectional elongated flake cores, from non-standardized “expedient” flake cores, or in blade core repair and maintenance, such as in crest shaping, platform preparation, lateral or distal convexity correction, or even in blade core production surface rejuvenation. There are no typical Levallois, Discoid or Quina flakes.

Tool configuration and use

The retouched tool assemblage is composed of 117 tools (5.3% of the flint-only assemblage). All of them, except for one backed bladelet on unidentified flint, are made on Flysch flint. Tool configuration and maintenance is an important activity at the site, specially with regard to the production of Châtelperronian points in Area 3. Most of the proximal and distal fragments of points recovered were abandoned after some accident during back configuration. This contrasts with the abundance of finalized points among the out-of-context materials (SI-5). Interestingly, five accidental resharpening flakes originated during back configuration have been identified (Figure S31). The other tools at

the site do not show great investment in configuration or maintenance, with some exceptions like the two endscrapers on flakes and one sidescraper.

The best-represented retouched tools are marginally backed blades (23.1%) including fully, partially, pointed and inversely retouched blades (Figure 3: 21). These marginally backed blades have been described at Arcy-sur-Cure [25], Vieux Coutets [24], Canaule II [22] and Labeko Koba [27], among others, and are characteristic of the Châtelperronian technocomplex. Châtelperronian points represent 7.7% of the retouched toolkit: with two exceptions that are not broken, most of them are distal fragments. These fractures were produced, very likely, during the fabrication process [28]. There is a single Châtelperronian point with a fracture that can be linked with an impact (Figure 3: 8). The backs tend to be unidirectional, thicker in the 3/4 section of the back and slightly curved in the 4/4 section. There is also also one point with marginal back except in the 3/4 section of the back (Figure 3: 1), similar to a piece described for Labeko Koba [27], and others described for Canaule II, Le Basté or Bidart [23]. Another eight pieces are backed blades (6.8%), four of which present curved backs with typical Châtelperronian retouching, so are very likely proximal fragments of Châtelperronian points. Other typical tools like wide endscrapers (Figure 3: 31), with only two examples, are very scarce in the assemblage. Among the retouched bladelets (12%), there are 1 typical Dufour, 5 backed, 5 marginally backed (Figure 3: 23-27), 2 retouched and 1 truncated bladelets. The presence of retouched bladelets has been noted in Châtelperronian sites like Quinçay [26], and the abundance of these at Aranbaltza is probably linked with the careful sieving of the sediments. Other tools like retouched blades, burins, borers, truncations, notches, denticulates, sidescrapers, splintered pieces, marginally backed flakes and retouched flakes represent 47.9% of the retouched toolkit (Figure S32). With some exceptions, these tools are not very standardized and probably represent a sort of opportunistic activity of rather non-specific tool fabrication and use at the site.

Besides the retouched tools, many blades and flakes show edge attrition, probably related with use, but the use-wear preservation problems have hindered more precise analysis of function. However, the similarities with other coeval assemblages, such as Vieux Coutets, suggest that different activities, apart than flint knapping, were undertaken at Aranbaltza II.

There are also a few tools on sandstone that have been used in percussion activities, having served as hammerstones and anvils. In Area 3 there is a thick pebble fragment (112.7 x 96 x 61.4 mm) with percussion traces on two protuberant areas (Figure S33: 3). In Area 2, there is a big hammerstone/anvil made on fine-grained sandstone (94.6 x 78.4 x 55.8 mm). One of the large faces has been artificially flattened and bears an irregular depression caused by use as an anvil. The opposed face, which is convex, has similar traces at the top of the convex area. The piece also displays traces of percussion (direct) on both short extremities, and similar traces on one of the long and narrow surfaces (Figure S33: 1). We have also recorded a flat sandstone pebble, with percussion traces on one of the short and narrow edges, and a typical hinged fracture on the opposed edge (94.7 x 60.4 x 21 mm) (Figure S33: 2).

Ochre use

In Area 1, there is one fragment of ferruginous nodule form (#4019) with light faceting of one extremity, that could be due to post-depositional alterations (Figure S34: 1). In addition, there are another 29 small lumps of ochre without apparent traces of processing (Figure S34: 2-5). Although ochre can be found naturally in the sandstone strata and in the conglomerates that appear in the surroundings, we cannot rule out the possibility that these fragments are the result of ochre processing at the site. Further, the very nature of the sediment, with iron oxide precipitates, hampers the clear identification of ochre residues on the pieces. Nevertheless, there are one partially cortical blade (Figure S34: 7), one wide blade fragment (Figure S34: 6), one flank blade fragment, and one Châtelperronian point fragment (Figure 3: 5) bearing clear deposits of ochre.

SI-7. AMS ^{14}Cdating

Ana B. Marín-Arroyo
Lee J. Arnold
Martina Demuro
Joseba Rios-Garaizar
Amaia Arranz-Otaegi

In the absence of bone material, charcoal fragments from the Aranbaltza II sequence were selected for radiocarbon (^{14}C) dating. The only clear combustion feature was identified in the US2 layer (Chalcolithic–Early Bronze Age), where a simple hearth has been recognized. Selected charcoal fragments from US2, US3 and US4b were targeted for dating, but these represented isolated fragments and cannot be linked directly to human activity at the site.

Thirteen ^{14}C samples were analysed at Beta Analytics (USA), the Radiocarbon Accelerator Facility at Oxford University (UK) and the Poznan Radiocarbon Laboratory (Poland). The ^{14}C samples were prepared using standard acid-base-acid (AAA) pre-treatment (e.g. [97]), with isotopic fractionation corrected for using measured ^{13}C values. The 14C dating results have been calibrated with the IntCal20 curve [98] using OxCal v4.4 [77-78].

The analyzed charcoal fragments from US4b, either did not yield any results or produced unrealistically young ages that are incompatible with the archaeological assemblage (Figure S34). Some of the fragments dissolved during pre-treatment and thus, could not be further analyzed. This is probably related to post-depositional alteration of charcoal, rather than selection of non-charcoal materials, as some of these fragments preserved vegetation structures that allowed precise taxonomic classification (L. Zapata pers. communication). The two well-preserved charcoal fragments retrieved from US4b (OxA-34891 and OxA-34987) yielded very young ages that were identical to those obtained on overlying US3, attributed to the Mesolithic. This may be explained by lixiviation of the organic fraction, which likely contaminated the samples, or by bioturbation (basically roots). Given that these charcoal ages were obtained using standard AAA pretreatment procedures that may not completely remove exogenous carbon [99-100], they are conservatively interpreted as minimum age indicators for the US4b Châtelperronian occupations.

SI-8 Single-grain OSL dating experimental procedures and results

Lee J. Arnold
Martina Demuro
Carlos Pérez-Garrido

Equivalent dose (D_e) determination

Eight single-grain OSL dating samples were collected from Units 3 to 4c to provide estimates of when sedimentary quartz grains were last exposed to light prior to burial. Six of these samples were taken from the recently excavated B and C Sections in 2013 (samples AZ13-5 and AZ13-6 = Unit 3; sample AZ13-3 = Unit 4a; samples AZ13-1, 2 and 4 = Unit 4c). The remaining two OSL samples were collected in 2016 from Unit 4b deposits exposed along Section A (samples AAM16-10 and AAM16-11). All samples were collected from cleaned exposure faces using opaque PVC tubes, and were immediately sealed with light-proof plastic upon extraction. Approximately 500 g of additional bulk sediment was collected from material directly surrounding each sample for dosimetry and water content assessments.

Quartz grains were processed under safe light (dim red LED) conditions at the CENIEH luminescence dating laboratory using standard preparation procedures (e.g., [67]), including a 48% hydrofluoric acid etch (40 minutes) to remove the alpha-irradiated outer layers of the quartz extracts.

Single-grain D_e measurements were made using Risø TL-DA-20 readers equipped with blue LED units, infrared LEDs, and 10 mW Nd:YVO_4 single-grain laser attachments emitting at 532 nm. Ultraviolet OSL signals were detected using EMI 9235QA/B photomultiplier tubes fitted with 7.5 mm-thick Hoya U-340 filters. Samples were irradiated with mounted $^{90}Sr/^{90}Y$ beta sources that had been calibrated to administer known doses to multi-grain aliquots and single-grain discs. Purified quartz grains with a diameter of 212-250 µm were manually loaded onto aluminium discs drilled with a 10 x 10 array of 300 µm diameter holes to ensure true single-grain resolution during equivalent dose (D_e) evaluation [101].

For the 2013 samples, individual D_e values were determined using the single-aliquot regenerative-dose (SAR) procedure [102] shown in Table S9a, which yielded suitable multiple-grain aliquot and single-grain dose-recovery test results for sample AZ13-3 (see below). The D_e values of the 2016 sample were measured independently using the SAR procedure shown in Table S9b, which is based on that used previously for OSL samples from Aranbaltza III [33]. Sensitivity-corrected dose-response curves were constructed using the first 0.08 s of each OSL stimulation after subtracting a mean background count obtained from the last 0.25 s of the signal.

Between 2800 and 3400 single-grain D_e measurements were made for each sample (Table S10), with individual D_e values being included in the final age calculation if they satisfied a series of standard and widely tested quality-assurance criteria (e.g., [76]). Specifically, single-grain OSL D_e estimates were rejected from further

consideration if they exhibited one or more of the following properties: (i) weak OSL signals (i.e., the net intensity of the natural test-dose signal (T_n) was less than three times the standard deviation of the late-light background signal); (ii) poor recycling ratios (i.e., the ratios of sensitivity-corrected luminescence responses (L_x/T_x) for two identical regenerative doses were not consistent with unity at 2σ); (iii) high levels of signal recuperation / charge transfer between SAR cycles (i.e., the sensitivity-corrected luminescence response of the 0 Gy regenerative dose point amounted to >5% of the sensitivity-corrected natural signal response (L_n/T_n) at 2σ); (iv) anomalous dose-response curves (i.e., those displaying a zero or negative response with increasing dose) or dose-response curves displaying very scattered L_x/T_x values (i.e., those that could not be successfully fitted with the Monte Carlo procedure and, hence, did not yield finite D_e values and uncertainty ranges); (v) saturated or non-intersecting natural OSL signals (i.e., L_n/T_n values equal to, or greater than, the I_{max} saturation limit of the dose-response curve at 2σ); (vi) extrapolated natural signals (i.e. L_n/T_n values lying more than 2σ beyond the L_x/T_x value of the largest regenerative-dose administered in the SAR procedure); (vii) contamination by feldspar grains or inclusions (i.e., the ratio of the L_x/T_x values obtained for two identical regenerative doses measured with and without prior IR stimulation (OSL IR depletion ratio [103]) was less than unity at 2σ). The OSL grain classification statistics obtained for each sample after applying these SAR quality assurance criteria are summarised in Table S10.

Individual and sample-averaged D_e estimates are presented throughout this paper with their 1σ uncertainties, which are derived from three sources of uncertainty: (i) a random uncertainty term arising from photon counting statistics for each OSL measurement, calculated using Eq. 3 of Galbraith [104]; (ii) an empirically determined instrument reproducibility uncertainty of 1.8% for each single-grain measurement, calculated using the procedure outlined in Jacobs et al. [105]; and (iii) a dose-response curve fitting uncertainty determined using 1000 iterations of the Monte Carlo method described by Duller [106] and implemented in Analyst v4.

Dose rate determination

The environmental dose rates for the Aranbaltza II OSL samples have been calculated using a combination of *in situ* field gamma spectrometry and low level beta counting, as detailed in Table S11. Gamma dose rates were determined from *in situ* measurements made using a Canberra NaI:Tl detector to account for any spatial heterogeneity in the surrounding (~30 cm diameter) gamma radiation field of each sample. The 'energy windows' approach described in Arnold et al. [107] was used to derive individual estimates of U, Th and K concentrations from the field gamma-ray spectra. External beta dose rates were determined from measurements made using a Risø GM-25-5 beta counter [108] on dried and homogenised, bulk sediments collected directly from the OSL sampling positions. This approach was used to ensure that beta dose rates were derived from sample sizes that more closely approximate the very short (~2-3 mm) beta particle radiation fields affecting these samples. Background-subtracted count rates were measured for three aliquots of each sample and compared with net count rates obtained simultaneously for a loess sediment standard with known U, Th and K

concentrations [109]. Final beta dose rate estimates were calculated after making allowance for beta dose attenuation due to grain-size effects and HF etching [110].

Cosmic-ray dose rates were calculated using the approach described in Prescott and Hutton [111] after taking into consideration site altitude, geomagnetic latitude, and density / thickness of sediment and bedrock overburden. A small, assumed internal (alpha plus beta) dose rate of 0.03 ± 0.01 Gy / ka has been included in the final dose rate calculations based on published ^{238}U and ^{232}Th measurements for etched quartz grains from a range of locations (e.g., [112-115] and an alpha efficiency factor (*a*-value) of 0.04 ± 0.01 [116-117].

Radionuclide concentrations and specific activities have been converted to dose rates using the conversion factors given in Guérin et al. [118], making allowance for beta-dose attenuation [110, 119] and long-term sediment water contents [120-121]. Seven of the eight OSL dating samples (AZ13-1 to AZ13-6 and AAM16-11) exhibit broadly consistent present-day sediment water contents of 11 to 20% dry sediment weight (mean water content ± 1σ = 14 ± 2%), which overlap with published values for similar types of fluvial and alluvial deposits from Spain [75, 122-124]. The present-day sediment water contents of these seven samples are considered representative of moisture conditions prevailing throughout the sample burial period (particularly as they were collected from newly exposed excavation trenches), hence they have been used in the final dose rate calculations.

The remaining sample (AAM16-10) yielded an uncharacteristically low present-day sediment water content of <6%. It is unclear whether the low moisture content recorded for this sample reflects localised drying out of the exposure face immediately prior to sampling, or if it is an experimental artefact of the laboratory procedures (e.g., the bulk sediment bag was not properly sealed following sampling). The lack of consistency with all other samples from the site, as well as with samples collected from similar deposits across the region (see above), suggests that this empirical water content estimate may not be entirely representative of the moisture conditions prevailing throughout the sample burial period. To determine a more suitable long-term sediment moisture content for this sample, we have adopted a more conservative estimate of 11% dry sediment weight, which is equivalent to the average measured water contents of the two closest related OSL samples collected from comparable deposits (i.e., Unit 4b and the upper horizons of Unit 4c = samples AAM16-11 and AZ13-4). A relative 1σ uncertainty of 20% has been assigned to the long-term moisture estimates of all eight samples to accommodate any minor variations in hydrologic conditions during burial.

While a long-term water content of 11 ± 2% has been chosen for the final dose rate calculation of sample AAM16-10, it is worth noting that use of the measured present-day water content (5.9 ± 1.2%) would not alter the final age estimate of this sample beyond its existing 1σ uncertainty range. Specifically, use of a long-term sediment moisture content of 5.9 ± 1.2% would cause the OSL age of AAM16-10 to decrease by 2.2 ka, with the resultant age of 41.3 ± 2.6 ka remaining statistically indistinguishable from that shown in Table S11 at 1σ (43.5 ± 2.9 ka). Our final

chronological interpretations for this sample are therefore relatively insensitive to the preferred choice of long-term water content.

SAR validation test results

The suitability of the SAR D_e determination procedure used for the 2013 samples (Table S9a) was evaluated by undertaking a series of multi-grain aliquot and single-grain dose-recovery tests on sample AZ13-3. Multi-grain aliquot dose-recovery tests were first used to ascertain optimal preheating conditions for bulk grain populations. These tests were performed on ~180-grain aliquots using a series of different regenerative dose preheat (PH_1) conditions (ranging between 200 ºC for 10 s and 260 ºC for 10 s) and different test dose preheat (PH_2) combinations (160 ºC for 10 s and 200 ºC for 10 s). A known laboratory dose of 51 Gy was applied to groups of 4 aliquots after optically bleaching their natural OSL signals using two 1,000 s blue LED stimulations separated by a 10,000 s pause (to ensure complete decay of any phototransferred charge in the 110 ºC TL trap). The administered dose was treated as a surrogate natural dose and subsequently measured using a multi-grain version of the SAR sequence shown in Table S9a, which involved replacing 125°C green laser stimulations with 125°C blue LED stimulations for 60 s, and inserting a 50°C IR bleach for 40 s prior to each OSL measurement to remove any feldspar signal contamination. Figure S36a summarises the results of the multi-grain aliquot dose-recovery tests performed on sample AZ13-3. The most suitable dose-recovery results were obtained using a PH_1 of 200 ºC for 10 s and a PH_2 of 160 ºC for 10 s. This preheat combination yielded a weighted mean measured-to-given dose ratio of 0.99 ± 0.02, low inter-aliquot D_e scatter, low-dose and high-dose mean recycling ratios in agreement with unity at 1σ (0.99 ± 0.02 and 1.01 ± 0.01, respectively) and a mean recuperation ratio of less than 1%.

To confirm the suitability of this SAR procedure at the single-grain scale, we repeated the dose-recovery test on 1200 individual quartz grains from sample AZ13-3 using the optimum multi-grain preheat conditions (PH_1 = 200 ºC for 10 s, PH_2 = 160 ºC for 10 s). A dose of 50 Gy was administered to these quartz grains after bleaching their natural signals using the same procedure described above. The single-grain OSL dose recovery test yielded a mean measured-to-given dose ratio of 0.97 ± 0.03 (n = 42 accepted grains) and an overdispersion value of 9 ± 4%, confirming the suitability of the chosen preheat combination for this sample (Figure S36b).

The D_e values of the 2016 OSL samples were measured independently from the 2013 OSL samples, and employed the SAR procedure shown in Table S9b, which is based on that used previously at the neighbouring site of Aranbaltza III [33]. This SAR procedure involves a PH_1 of 220 ºC for 10 s and a PH_2 of 200 ºC for 10 s, and an additional high temperature OSL treatment at the end of each cycle to prevent signal carry over from previous L_x and T_x measurement steps (OSL 'hot wash' stimulation at 260ºC for 40 s). A single-grain dose recovery test was performed on sample AAM16-10 to confirm the suitability of this SAR procedure for the 2016 Aranbaltza II samples. A dose of 40 Gy was administered to 2400 quartz grains of sample AAM16-10 after bleaching their natural signals using the procedure detailed above for the 2013 samples.

The SAR procedure shown in Table S9b was then applied to these optically bleached and artificially dosed grains to determine their surrogate D_e values. This approach yielded a measured-to-given dose ratio consistent with unity at 1σ (1.00 ± 0.03) and an overdispersion value of 14 ± 3% (Figure S36c). The dose recovery results for sample AAM16-10 are comparable with those obtained for the 2013 samples, confirming the suitability of both SAR procedures in this study.

Single-grain OSL D_e results and ages

Table S11 provides a summary of the environmental dose rates, single-grain D_e values and final ages obtained for the eight OSL dating samples from Units 3 to 4c. Between 2 and 4% of quartz grains measured per sample were considered suitable for OSL dating purposes after application of the SAR quality assurance criteria (Table S10), with the majority of measured grains (70–88%) being rejected from further consideration because they exhibited weak or no OSL signals. Representative OSL dose-response and decay curve for grains that passed the quality assurance criteria are shown in Figure S37. The majority of accepted grains display rapidly decaying OSL curves (reaching background levels within 0.5 s), and the single-grain OSL dose-response curves are generally well-represented by either a single saturating exponential function or a saturating exponential plus linear function.

The single-grain D_e distributions of grains that passed the SAR quality assurance criteria are shown as radial plots in Figure S38a-h. Four of the OSL samples (samples AZ13-1, AZ13-2, AZ13-4 from Unit 4c and sample AAM16-10 from Unit 4b) share similar D_e distributions characterised by moderate dose dispersion (relative D_e range = 2.0 to 2.3), D_e scatter that is reasonably well-represented by the weighted mean value (as indicated by the large proportions of grains lying within the 2σ grey bands), and low-to-moderate overdispersion of 25 ± 3% to 35 ± 5% (Figure S38d, f-h). The overdispersion values for these samples are broadly similar to those reported for well-bleached and unmixed single-grain OSL D_e datasets from similar settings across the Iberian Peninsula [76, 123], though they are slightly higher than the average overdispersion of 20 ± 1% reported for ideal samples by Arnold and Roberts [125] and systematically higher than the overdispersion values of 10 ± 4% to 14 ± 3% obtained for the single-grain dose recovery test of samples AZ13-3 and AAM16-10. None of these four D_e datasets are considered significantly positively skewed according to the weighted skewness test outlined by Bailey and Arnold [126] and Arnold and Roberts [127]; though sample AAM16-10 is considered to be significantly negatively skewed when compared with its 95.4% critical skewness score (i.e., twice the standard error of skewness score; [126]) (Table S12). Application of the maximum log likelihood (L_{max}) test [128] indicates that the central age model (CAM) is statistically favoured over the three- or four-parameter minimum age models (MAM-3 or MAM-4) of Galbraith et al. [129] for all four D_e datasets (Table S12).

Collectively, these single-grain OSL D_e characteristics suggest that samples AZ13-1, AZ13-2, AZ13-4 and AAM16-10 do not suffer from major extrinsic D_e scatter related to insufficient bleaching prior to burial [130-131] or widespread post-depositional

sediment mixing between units [75, 107]. It therefore seems likely that the low-to-moderate overdispersion and negatively skewed / normally distributed D_e dispersion observed for these samples is attributable to intrinsic experimental scatter not captured by the dose recovery test (e.g., grain-to-grain variations in luminescence responses due to the fixed SAR conditions; [74]) or extrinsic field-related scatter associated with beta-dose spatial heterogeneity [132-133]. Pedogenic processes that are known to have locally affected parts of Units 4b and 4c may have additionally contributed to the D_e scatter for these four samples. However, these processes do not appear to have resulted in widespread mixing of different aged grain populations at the sampling localities. The single-grain OSL ages for AZ13-1, AZ13-2, AZ13-4 and AAM16-10 have been obtained using the weighted mean (CAM) D_e estimate, in accordance with their L_{max} test results [128] (Tables S11-S12).

The remaining four OSL samples from Aranbaltza II (AZ13-5 and AZ13-6 from Unit 3, AZ13-3 from Unit 4a, and AAM16-11 from Unit 4b) exhibit more complex D_e distributions characterised by high dose dispersion (relative D_e range = 4.5 to 7.2), larger proportions of individual D_e values lying outside of the weighted mean burial dose 2σ ranges, and distinct leading-edges of low D_e values or tails of higher D_e values (Figure S38a-c, e). These single-grain D_e datasets are additionally considered to be significantly positively skewed according to the weighted skewness test outlined by Bailey and Arnold [126] and Arnold and Roberts [125] (Table S12). With the exception of AZ13-6, these samples also yield overdispersion values that are significantly higher (54 – 70%) than those obtained for the 'ideal' (well-bleached and unmixed) sedimentary samples from this site (e.g., sample AZ13-4 = 25 ± 3%) and from broader single-grain OSL datasets [125]. Application of the L_{max} test [128] indicates that either the MAM-3 or MAM-4 are statistically favoured over the CAM for all four D_e datasets (Table S12). These various D_e characteristics are consistent with those commonly reported for heterogeneously bleached single-grain OSL samples [126, 134-135], which seems reasonable in this sedimentary context given the host deposits were deposited by fluvial and alluvial processes that could have involved limited transportation distances, UV-filtered (subaqueous) daylight exposures or localised erosion and entrainment of pre-existing deposits (syn-depositional mixing). In the case of AZ13-3 and AAM16-11, it is also possible that the high tails of D_e values may partly relate to sampling issues. These two samples were collected from the lowermost horizons of units that displayed undulating and laterally variable sedimentary boundaries, and it is possible that the PVC tubes incorporated the interfaces of underlying sedimentary units.

On the basis of these D_e interpretations, we have opted to use the MAM to derive the burial doses for samples AZ13-3, AZ13-5, AZ13-6 and AAM16-11 (Tables S11-S12). The decision of whether to use the MAM-3 or MAM-4 for the final age calculation of each sample has been made on statistical grounds using the L_{max} score, as outlined by Arnold et al. [128]. It is worth emphasising that for the two samples that may have suffered sampling complications, application of the finite mixture model (FMM) instead of the MAM would not alter the final age estimates beyond their existing uncertainty ranges (Table S12). In both cases, the FMM identifies two discrete dose components, with the dominant dose component yielding a final age of 12.1 ± 0.9 ka for sample AZ13-

3 (compared to a MAM-4 age of 11.9 ± 0.9 ka) and 61.4 ± 4.8 for sample AAM16-11 (compared to a MAM-3 age of 51.9 ± 4.0 ka).

The reliability of the final OSL results is supported by the overall stratigraphic agreement of the eight single-grain OSL ages (Table S11), and by the consistency (at 1 or 2σ) of the replicate or closely associated ages obtained on individual sedimentary units (e.g., samples AZ13-5 and AZ13-6; samples AZ13-2 and AZ13-1). The OSL ages obtained for Unit 4c indicate a series of aggradation events and paleosol development phases took place prior to the Chatelperronian occupation of Aranbaltza II between late MIS 5 and early MIS 3. The various OSL ages obtained on Units 4b and 4c provide bracketing (indirect) age constraint on the Chatelperronian layer, with sample AAM13-10 providing the closest and most secure estimate for the timing of the Chatelperronian occupation ~43.5 ± 2.9 ka (1σ).

SI-9 Bayesian modelling of available ages for the Châtelperronian culture and associated hominin remains across France and the Iberian Peninsula

Ana B. Marín-Arroyo
Lee J. Arnold
Martina Demuro
Asier Gómez-Olivencia
Joseba Rios-Garaizar

Bayesian modelling has been used to constrain the timing of Châtelperronian layers at individual sites across France and Iberia, and to derive a combined age estimate for the Châtelperronian culture across southwest Europe. For this purpose, we have considered all published radiometric age estimates that are methodologically reliable, are unaffected by post-depositional complications and have direct or indirect stratigraphic association with Châtelperronian layers (according to the information provided in the original publications). The published radiocarbon, OSL and TL ages that meet these initial criteria are summarised in Tables S13-S14, and have been derived from the French sites of Grotte du Renne, Saint-Césaire, Les Cottés, Grotte des Fées, La Quina Amont, La Ferrassie and Vieux Coutets, as well as the Iberian sites of Aranbaltza (this study), Labeko Koba, Ekain and Cova Foradada. As noted in the footnotes of Tables S13 and S14, we have cautiously excluded three of the age estimates from these various sites on the grounds of methodological complications associated with sample pretreatment procedures (sample Beta-414539 from layer IV at Cova Foradada) and equivocal stratigraphic association with Châtelperronian layers (sample ETH-99102 from La Ferrassie 8 and sample OxA-18099 from Saint Césaire, both belonging to hominin remains). However, we note that inclusion or exclusion of these three ages does not significantly alter our final modelled estimate for the regional timing of the Châtelperronian culture, especially when considering the size of the existing 95.4% C.I. The 32 ^{14}C ages included in our model from the Châtelperronian layers at Grotte du Renne are based on the two most recent ^{14}C dating studies of this site [12, 14]. The ^{14}C ages of Higham et al. [13] have not been included in our Bayesian modelling analysis owing to their poor internal consistency and a lack of consensus on the stratigraphic interpretations of this dataset (see discussions in [12, 14, 136-137]. At Grotte du Renne, Châtelperronian Layers IX and X are considered as a single chronological phase (based on lithic refits between these two layers [14]) and have been grouped accordingly in our model.

Bayesian modelling has been undertaken using OxCal v4.4.4 [77], which enables the integration of reliable numerical dating results (likelihoods) with all known stratigraphic and relative dating information (priors) to derive unified chronostratigraphic frameworks. To calculate the combined age range of the Châtelperronian layer(s) at sites that have multiple dating results, we have pooled all chronometric likelihoods in direct association with Châtelperronian deposits as a single, unordered *Phase* model with delineating start and end boundaries. The radiocarbon ages included in these site-specific models have been calibrated against the IntCal20 calibration curve [98]. Individual OSL and TL ages have been input into *Sequence* and *Phase* models after adjusting to the radiocarbon datum (AD1950) using the year of sample collection (or the year of publication if this information cannot be ascertained from the original study). The OxCal *Date* function [70] has been used to calculate the duration of the Châtelperronian layer(s) at individual sites from the posterior probabilities of the start and end boundaries of the site-specific *Phase* models.

Phase and *Sequence* models have been run using the general *Outlier* Function [78], with prior outlier probabilities of 5% equally assigned to all likelihood samples to identify potentially significant

statistical outliers that do not agree with the model framework. Likelihood estimates with posterior outlier probabilities >5% were not excluded from the final models; rather they were proportionally down-weighted in the Monte Carlo iterations [78].

To constrain the timing of the Châtelperronian culture in southwest Europe as a whole, we have pooled the modelled durations of the Châtelperronian layers at individual sites (derived from the site-specific *Phase* models using the *Date* command for sites that have multiple dating results) with the new OSL age obtained for the Aranbaltza Châtelperronian, as part of a single, unordered regional Châtelperronian *Phase* model. The combined age range for the Châtelperronian culture of southwest Europe has been calculated from the modelled posterior probabilities of the start and end boundaries of this regional *Phase* model using the *Date* function. The CQL code used to construct the site-specific and regional Châtelperronian *Phase* models are shown below.

The Bayesian model was run 4–5 times and the results compared to check for consistency. The repeated modelling runs revealed acceptable levels of reproducibility, with the corresponding posterior boundary and *Date* distribution ranges typically overlapping within a few decades of each other across the various model runs. Given this level of reproducibility, we have rounded all modelled age ranges reported in this study to the nearest decade.

The Bayesian modelling results for the regional Châtelperronian culture and the site-specific Châtelperronian layers are summarised in Figure 4 and Figure S39, respectively. Only one of the 67 likelihoods (sample EVA29) was identified as an extreme statistical outlier, returning a posterior outlier probability of 93% (Table S15). A further eighteen likelihoods exhibited posterior outlier probabilities ranging between 5 and 20%, and can therefore be considered minor statistical outliers; though it is worth noting that at least 3-4 outliers would be expected for a dataset of this size when adopting a prior outlier threshold of 5% [78]. The average convergence integral for all individual posterior distributions is 99.14 with individual convergence integrals exceeding 99.79% for all dating samples, supporting the overall effectiveness of the Monte Carlo solutions (Table S15). The combined age range for the Châtelperronian culture of southwest Europe (calculated from the regional *Phase* model using the *Date* function) is estimated to be 43,760-39,220 at the 95.4% C.I. (Figure 4; Table S15).

OxCal v4.4.4 CQL code

```
Plot()
 {
  Outlier_Model("General",T(5),U(0,4),"t");
  Sequence("Châtelperronian Grotte du Renne")
  {
   Boundary("Start Châtelperronian Grotte du Renne");
   Phase("Grotte du Renne Layer IX+X")
   {
    R_Date("EVA-33", 40970, 424)
    {
     color="mediumblue";
     Outlier("General", 0.05);
    };
    R_Date("EVA-28", 40930, 393)
```

```
{
 color="mediumblue";
 Outlier("General", 0.05);
};
R_Date("EVA-49", 40830, 778)
{
 color="mediumblue";
 Outlier("General", 0.05);
};
R_Date("EVA-34", 40520, 389)
{
 color="mediumblue";
 Outlier("General", 0.05);
};
R_Date("EVA-27", 40230, 395)
{
 color="mediumblue";
 Outlier("General", 0.05);
};
R_Date("EVA-51", 39960, 702)
{
 color="mediumblue";
 Outlier("General", 0.05);
};
R_Date("EVA-46", 39930, 361)
{
 color="mediumblue";
 Outlier("General", 0.05);
};
R_Date("EVA-47", 39750, 360)
{
 color="mediumblue";
 Outlier("General", 0.05);
};
R_Date("EVA-37", 39450, 340)
{
 color="mediumblue";
 Outlier("General", 0.05);
};
R_Date("EVA-26", 39390, 334)
{
 color="mediumblue";
 Outlier("General", 0.05);
};
R_Date("EVA-31", 39290, 334)
{
 color="mediumblue";
```

```
 Outlier("General", 0.05);
};
R_Date("EVA-44", 39280, 351)
{
 color="mediumblue";
 Outlier("General", 0.05);
};
R_Date("EVA-35", 39240, 341)
{
 color="mediumblue";
 Outlier("General", 0.05);
};
R_Date("EVA-48", 39070, 332)
{
 color="mediumblue";
 Outlier("General", 0.05);
};
R_Date("EVA-43", 39020, 352)
{
 color="mediumblue";
 Outlier("General", 0.05);
};
R_Date("EVA-41", 38730, 333)
{
 color="mediumblue";
 Outlier("General", 0.05);
};
R_Date("EVA-24", 38400, 317)
{
 color="mediumblue";
 Outlier("General", 0.05);
};
R_Date("EVA-42", 38070, 311)
{
 color="mediumblue";
 Outlier("General", 0.05);
};
R_Date("EVA-30", 37980, 284)
{
 color="mediumblue";
 Outlier("General", 0.05);
};
R_Date("EVA-36", 37740, 307)
{
 color="mediumblue";
 Outlier("General", 0.05);
};
```

```
 R_Date("EVA-40", 37510, 275)
 {
  color="mediumblue";
  Outlier("General", 0.05);
 };
 R_Date("MAMS-25149", 36840, 660)
 {
  color="red";
  Outlier("General", 0.05);
 };
 R_Date("EVA-23", 36840, 335)
 {
  color="mediumblue";
  Outlier("General", 0.05);
 };
 R_Date("EVA-32", 36820, 257)
 {
  color="mediumblue";
  Outlier("General", 0.05);
 };
 R_Date("EVA-38", 36540, 248)
 {
  color="mediumblue";
  Outlier("General", 0.05);
 };
 R_Date("EVA-25", 36210, 250)
 {
  color="mediumblue";
  Outlier("General", 0.05);
 };
 //originally excluded outlier
 R_Date("EVA-29", 35500, 216)
 {
  color="mediumblue";
  Outlier("General", 0.05);
 };
 Date("Duration Grotte du Renne Layer IX+X");
};
Boundary("Grotte du Renne Layer IX+X/VIII");
Phase("Grotte du Renne Layer VIII")
{
 //originally excluded outlier
 R_Date("EVA-56", 37710, 533)
 {
  color="mediumblue";
  Outlier("General", 0.05);
 };
```

```
  R_Date("EVA-55", 36630, 452)
  {
   color="mediumblue";
   Outlier("General", 0.05);
  };
  R_Date("EVA-53", 36230, 435)
  {
   color="mediumblue";
   Outlier("General", 0.05);
  };
  R_Date("EVA-52", 35980, 432)
  {
   color="mediumblue";
   Outlier("General", 0.05);
  };
  R_Date("EVA-54", 35380, 390)
  {
   color="mediumblue";
   Outlier("General", 0.05);
  };
  Date("Duration Grotte du Renne Layer VIII");
 };
 Boundary("End Châtelperronian Grotte du Renne");
};
Sequence("Châtelperronian Saint-Césaire")
{
 Boundary("Start Châtelperronian Saint-Césaire");
 Phase("Châtelperronian Saint-Césaire")
 {
  R_Date("OxA-21636", 37200, 1000)
  {
   color="mediumblue";
   Outlier("General", 0.05);
  };
  R_Date("OxA-21700", 36650, 750)
  {
   color="mediumblue";
   Outlier("General", 0.05);
  };
  R_Date("OxA-21699", 36000, 700)
  {
   color="mediumblue";
   Outlier("General", 0.05);
  };
  Date("Duration Châtelperronian Saint-Césaire");
 };
 Boundary("End Châtelperronian Saint-Césaire");
```

```
};
Sequence("Châtelperronian Les Cottés")
{
 Boundary("Start Châtelperronian Les Cottés");
 Phase("Châtelperronian Les Cottés")
 {
  R_Date("EVA-5", 42360, 370)
  {
   color="mediumblue";
   Outlier("General", 0.05);
  };
  R_Date("EVA-21", 41070, 300)
  {
   color="mediumblue";
   Outlier("General", 0.05);
  };
  R_Date("MAMS-10803", 38540, 270)
  {
   color="mediumblue";
   Outlier("General", 0.05);
  };
  R_Date("EVA-13", 38100, 210)
  {
   color="mediumblue";
   Outlier("General", 0.05);
  };
  R_Date("EVA-12", 37360, 610)
  {
   color="mediumblue";
   Outlier("General", 0.05);
  };
  R_Date("EVA-11", 36230, 210)
  {
   color="mediumblue";
   Outlier("General", 0.05);
  };
  Date("OSL-LC10-15", N(2010-43300,2300))
  {
   color="mediumblue";
   Outlier("General", 0.05);
  };
  Date("OSL-LC11-2", N(2011-43100,2100))
  {
   color="mediumblue";
   Outlier("General", 0.05);
  };
  Date("OSL-LC10-13", N(2010-38400,1800))
```

```
   {
    color="mediumblue";
    Outlier("General", 0.05);
   };
   Date("Duration Châtelperronian Les Cottés");
  };
  Boundary("End Châtelperronian Les Cottés");
 };
 Sequence("Châtelperronian Labeko Koba")
 {
  Boundary("Start Châtelperronian Labeko Koba");
  Phase("Châtelperronian Labeko Koba")
  {
   R_Date("OxA-22562", 38100, 900)
   {
    color="mediumblue";
    Outlier("General", 0.05);
   };
   R_Date("OxA-22561", 38000, 900)
   {
    color="mediumblue";
    Outlier("General", 0.05);
   };
   R_Date("OxA-22563", 37800, 900)
   {
    color="mediumblue";
    Outlier("General", 0.05);
   };
   R_Date("OxA-22560", 37400, 800)
   {
    color="mediumblue";
    Outlier("General", 0.05);
   };
   Date("Duration Châtelperronian Labeko Koba");
  };
  Boundary("End Châtelperronian Labeko Koba");
 };
 Sequence("Châtelperronian Grotte des Fées")
 {
  Boundary("Start Châtelperronian Grotte des Fées");
  Phase("Châtelperronian Grotte des Fées")
  {
   R_Date("OxA-13621", 40650, 600)
   {
    color="mediumblue";
    Outlier("General", 0.05);
   };
```

```
   R_Date("OxA-14320", 39240, 380)
   {
    color="mediumblue";
    Outlier("General", 0.05);
   };
   R_Date("OxA-13622", 39150, 600)
   {
    color="mediumblue";
    Outlier("General", 0.05);
   };
   Date("Duration Châtelperronian Grotte des Fées");
  };
  Boundary("End Châtelperronian Grotte des Fées");
 };
 Sequence("Châtelperronian La Quina Amont")
 {
  Boundary("Start Châtelperronian La Quina Amont");
  Phase("Châtelperronian La Quina Amont")
  {
   R_Date("OxA-21706", 39400, 1000)
   {
    color="mediumblue";
    Outlier("General", 0.05);
   };
   R_Date("OxA-21707", 38100, 900)
   {
    color="mediumblue";
    Outlier("General", 0.05);
   };
   Date("Duration Châtelperronian La Quina Amont");
  };
  Boundary("End Châtelperronian La Quina Amont");
 };
 Sequence("Châtelperronian Foradada")
 {
  Boundary("Start Châtelperronian Foradada");
  Phase("Châtelperronian Foradada")
  {
   R_Date("Beta-435465", 34750, 240)
   {
    color="mediumblue";
    Outlier("General", 0.05);
   };
   R_Date("OxA-X-2649-9", 34490, 320)
   {
    color="mediumblue";
    Outlier("General", 0.05);
```

```
  };
  R_Date("OxA-X-2650-9", 34300, 1000)
  {
   color="mediumblue";
   Outlier("General", 0.05);
  };
  Date("Duration Châtelperronian Foradada");
 };
 Boundary("End Châtelperronian Foradada");
};
Sequence("Châtelperronian La Ferrasie")
{
 Boundary("Start Châtelperronian La Ferrasie");
 Phase("Châtelperronian La Ferrasie")
 {
  Date("OSL-FER2", N(2015-42200,2900))
  {
   color="mediumblue";
   Outlier("General", 0.05);
  };
  R_Date("MAMS-21206", 40890, 500)
  {
   color="mediumblue";
   Outlier("General", 0.05);
  };
  R_Date("MAMS-25524", 40770, 650)
  {
   color="mediumblue";
   Outlier("General", 0.05);
  };
  R_Date("MAMS-25523", 39000, 510)
  {
   color="mediumblue";
   Outlier("General", 0.05);
  };
  R_Date("MAMS-21207", 38910, 390)
  {
   color="mediumblue";
   Outlier("General", 0.05);
  };
  R_Date("MAMS-16373", 37380, 390)
  {
   color="mediumblue";
   Outlier("General", 0.05);
  };
  R_Date("MAMS-25522", 36590, 390)
  {
```

```
   color="mediumblue";
   Outlier("General", 0.05);
  };
  R_Date("MAMS-21208", 36300, 300)
  {
   color="mediumblue";
   Outlier("General", 0.05);
  };
  Date("Duration Châtelperronian La Ferrasie");
 };
 Boundary("End Châtelperronian La Ferrasie");
};
Sequence("Châtelperronian Vieux Coutets")
{
 Boundary("Start Châtelperronian Vieux Coutets");
 Phase("Châtelperronian Vieux Coutets")
 {
  Date("VC-OSL1", N(2018-44600,4800))
  {
   color="mediumblue";
   Outlier("General", 0.05);
  };
  Date("TL-Bdx-11972", N(2018-41000,2000))
  {
   color="mediumblue";
   Outlier("General", 0.05);
  };
  Date("Duration Châtelperronian Vieux Coutets");
 };
 Boundary("End Châtelperronian Vieux Coutets");
};
Sequence("Châtelperronian Ekain")
{
 Boundary("Start Châtelperronian Ekain");
 R_Date("Châtelperronian Ekain OxA-34930", 34350, 550)
 {
  color="mediumblue";
  Outlier("General", 0.05);
 };
 Boundary("End Châtelperronian Ekain");
};
Sequence("Châtelperronian timing")
{
 Boundary("Start Châtelperronian");
 Phase("Châtelperronian timing")
 {
  Date("=Duration Grotte du Renne Layer IX+X");
```

```
  Date("=Duration Grotte du Renne Layer VIII");
  Date("=Duration Châtelperronian Saint-Césaire");
  Date("=Duration Châtelperronian Les Cottés");
  Date("=Duration Châtelperronian Labeko Koba");
  Date("=Duration Châtelperronian Grotte des Fées");
  Date("=Duration Châtelperronian La Quina Amont");
  Date("=Duration Châtelperronian Foradada");
  Date("=Duration Châtelperronian La Ferrasie");
  Date("=Duration Châtelperronian Vieux Coutets");
  Date("=Châtelperronian Ekain OxA-34930");
  Date("Aranbaltza II US4b OSL-AAM16-10", N(2016-43500,2900))
  {
   color="mediumblue";
   Outlier("General", 0.05);
  };
  Date("Duration Châtelperronian");
 };
 Boundary("End Châtelperronian");
};
```

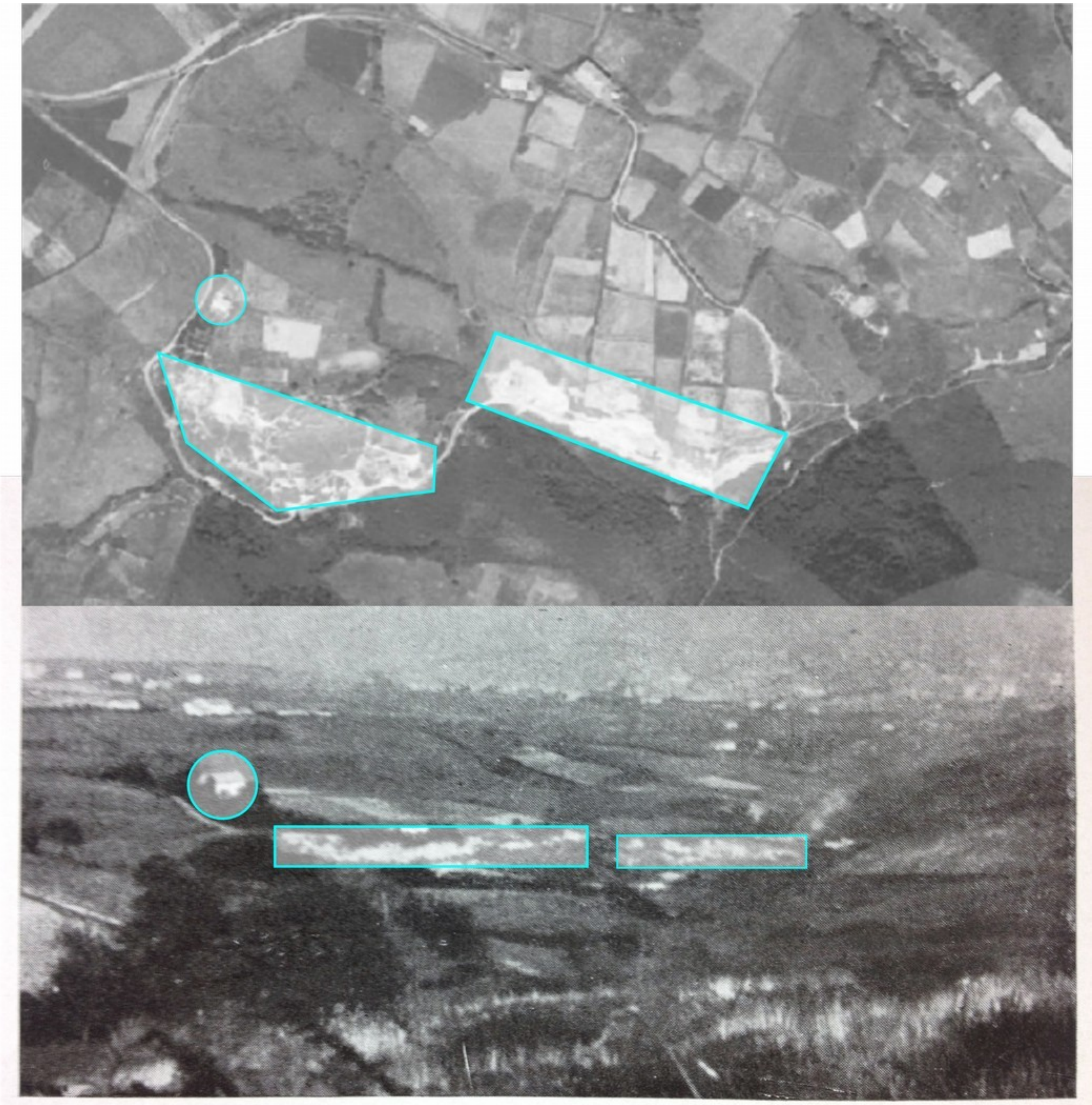

Fot. 3.—Arenal de Ollagorta. En el fondo, Plencia.

Fig. S1. Comparison between the 1965 aerial photo (https://www.geo.euskadi.eus), and the photo of Ollagorta quarry published by J.M. Barandiarán [18: Foto 3]. The circle marks the Ollagorta farmhouse, and the squares the west (left) and east (right) sides of the quarry. The east side is Aranbaltza.

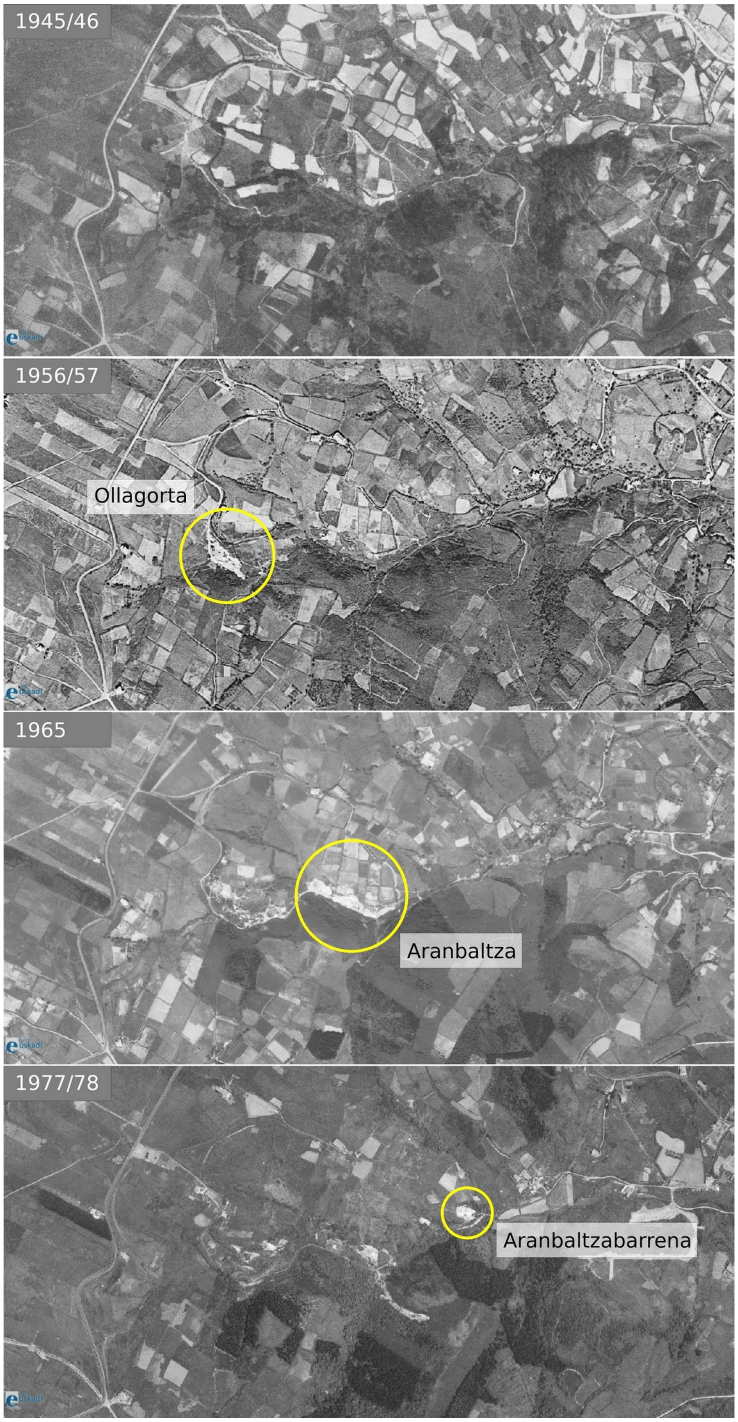

Fig. S2. Evolution of the sand quarry between 1946 and 1978. The analysis of a series of aerial photos taken by the U.S. Army (1946/47, 1956/57), Diputación Foral de Bizkaia (1965) and the Spanish Government (1977/78) show how the first quarry was opened at Ollagorta between 1946/47 and 1956/57, the second quarry at Aranbaltza between 1956/57 and 1965, and a final small quarry was opened close to Aranbaltzabarrena between 1965 and 1977/78. Aerial photos retrieved from https://www.geo.euskadi.eus.

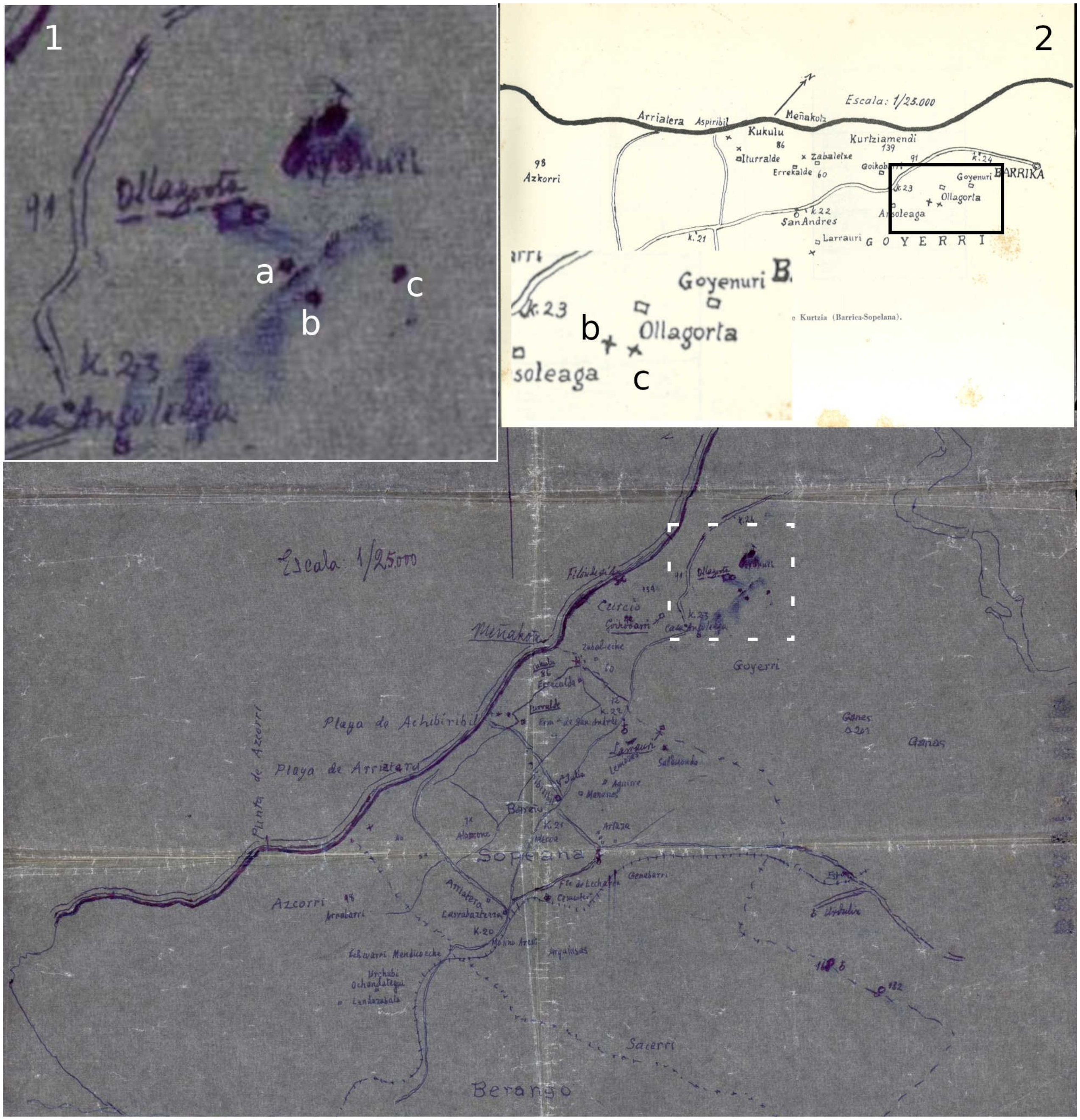

Fig. S3. Hand-drawn map of the Uribe Kosta region indicating the position of the excavation test sites (Courtesy of Barandiarán Foundation). 1) Closeup of the Ollagorta site, a-b western side excavations, c: eastern side excavation. 2) Simplified map published in 1960 [18: Fig. 1] with a closeup showing the correspondence to the west (b) and east (c) sides of the Ollagorta sand quarry.

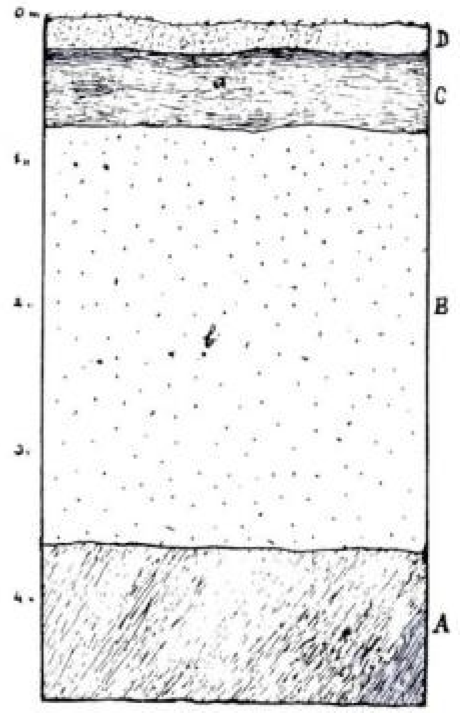

Corte del arenal (lado E.) de Ollagorta: D, humus reciente; C, arena rojiza; B, arena de color ceniza; A, arcilla.

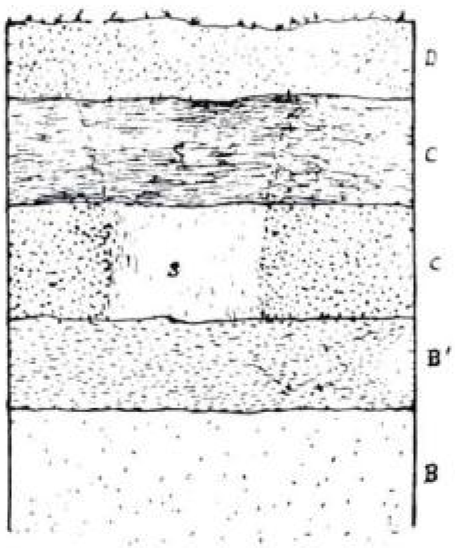

Corte del hogar (S) de Ollagorta.

Fig. S4. Eastern (left) and western (right) sections of Ollagorta site [18: Fig. 2-3).

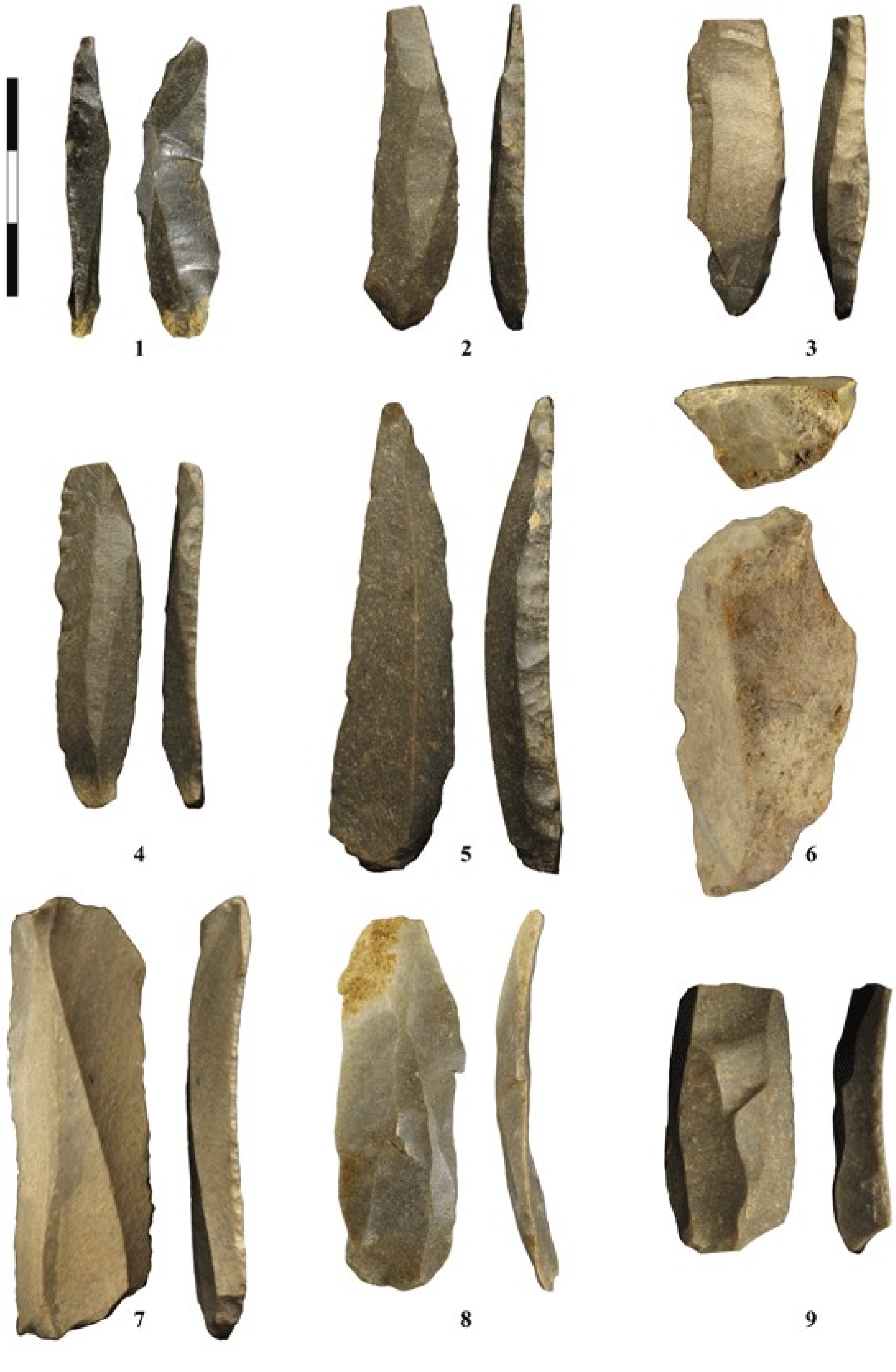

Fig. S5. Châtelperronian materials recovered by J.M. Barandiarán (Level C: 1, 6, 8) and A. Aguirre (2-5, 7, 9) in Ollagorta. 1-5 Châtelperronian points; 6: endscraper on cortical blade; 7-9: marginally backed blade [19].

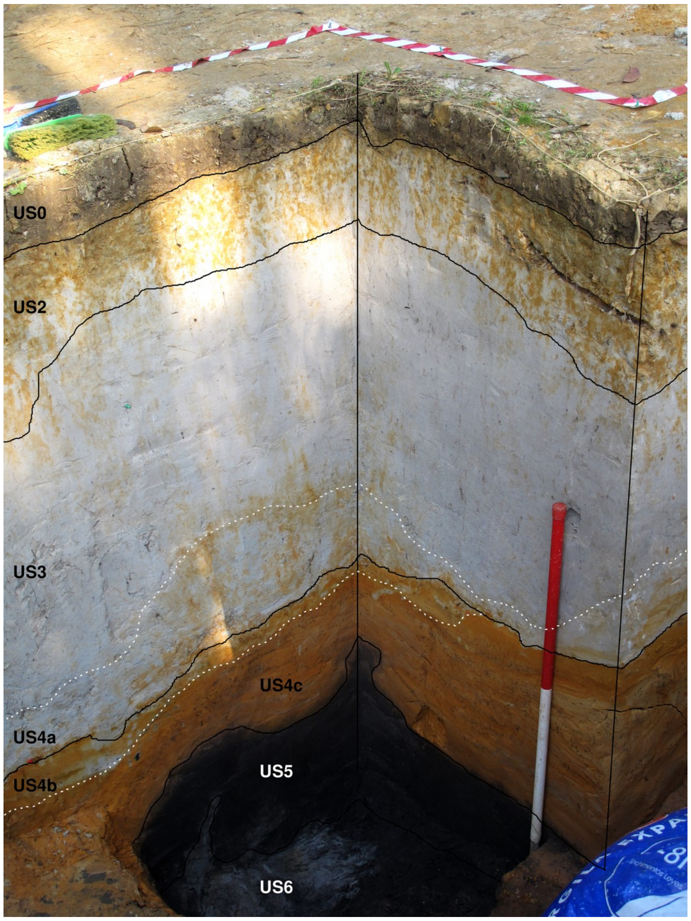

Fig. S6. NW corner section of Aranbaltza II (2013, Area 1).

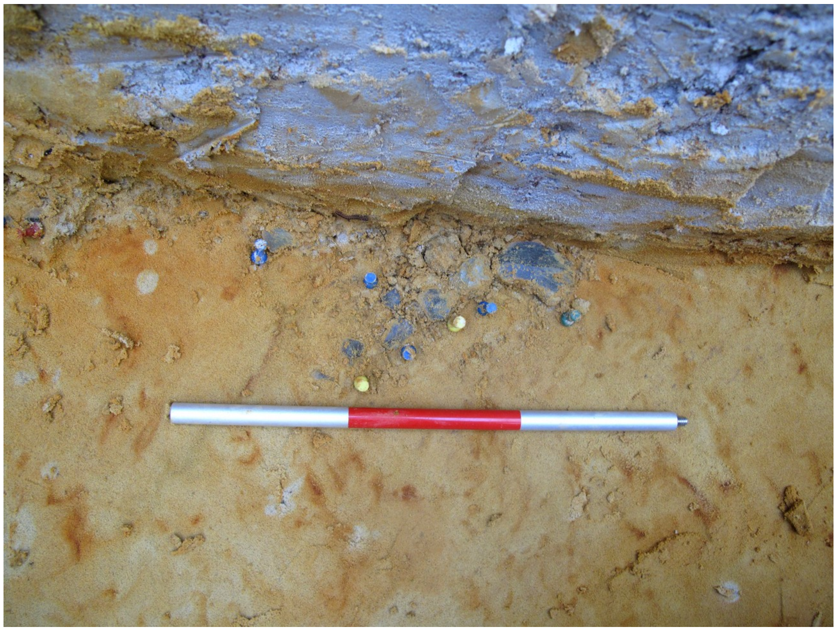

Fig. S7. Concentration of materials in US4b (2013, Area 1).

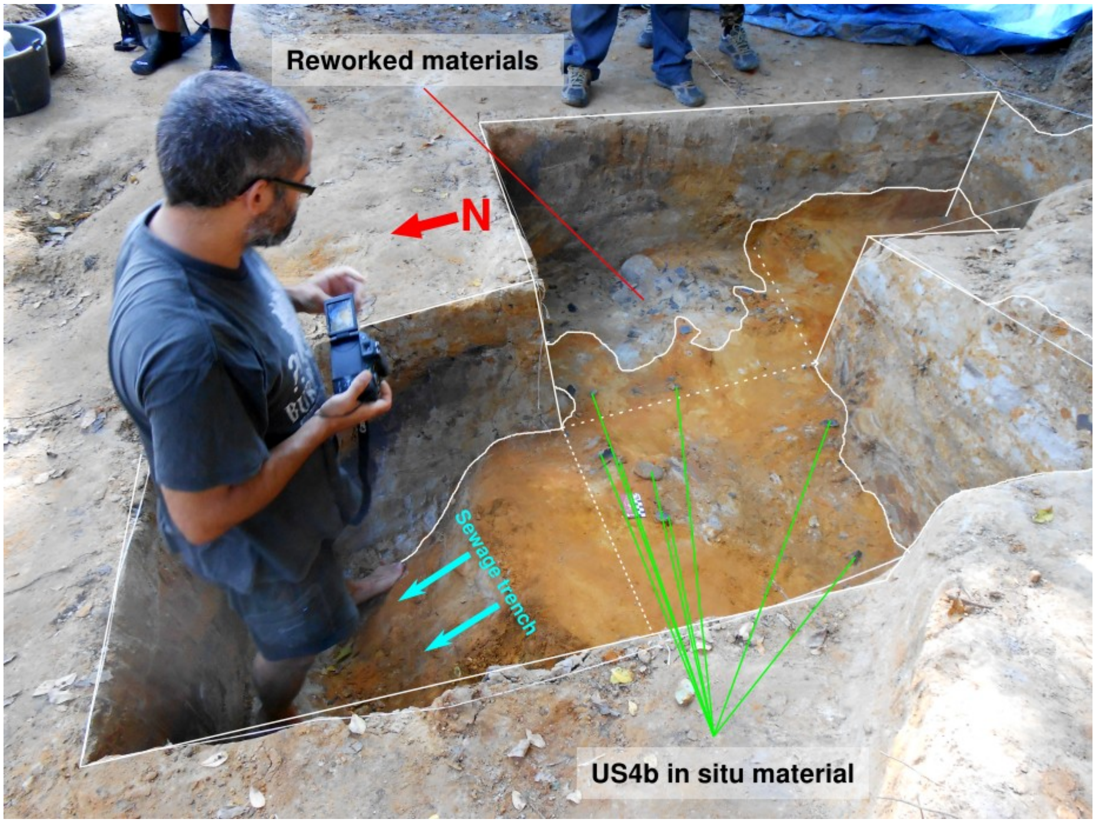

Fig. S8. 2014 excavation surface (Area 2) with US4b materials on surface. In the photo appears Joseba Rios-Garaizar (Photo: I. Libano).

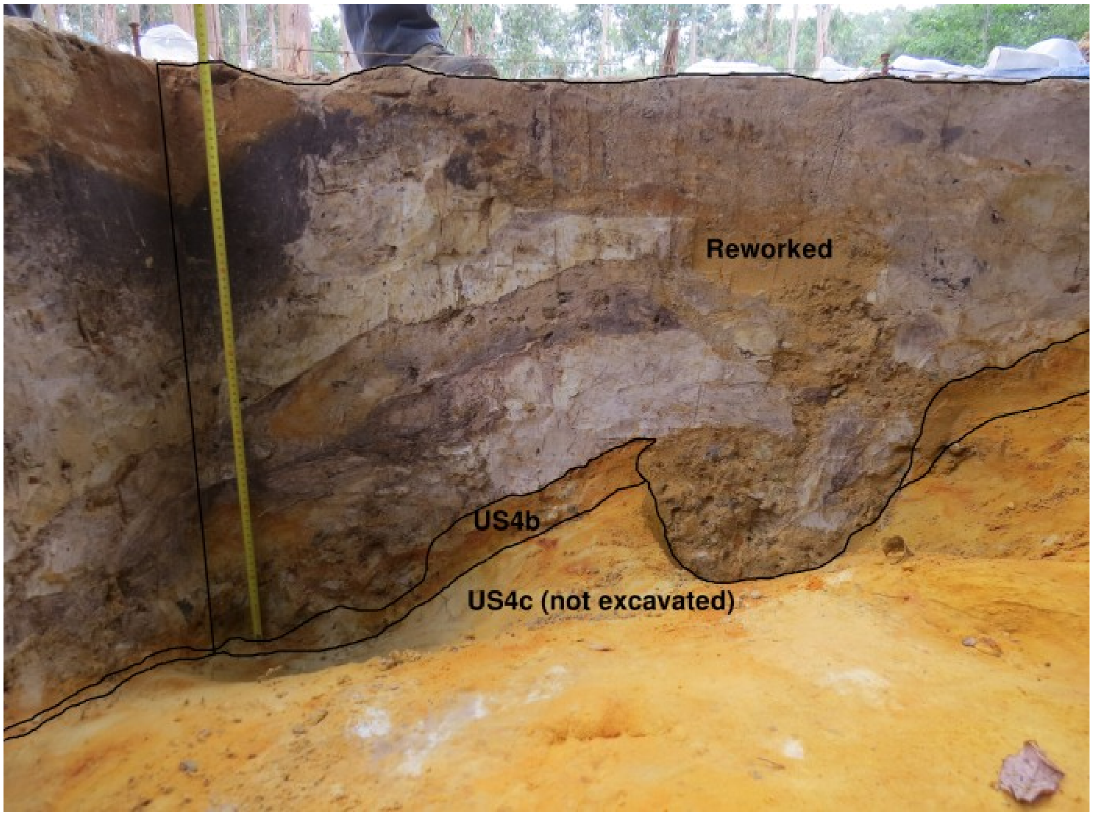

Fig. S9. Eastern section of the 2014 excavation area (Area 2).

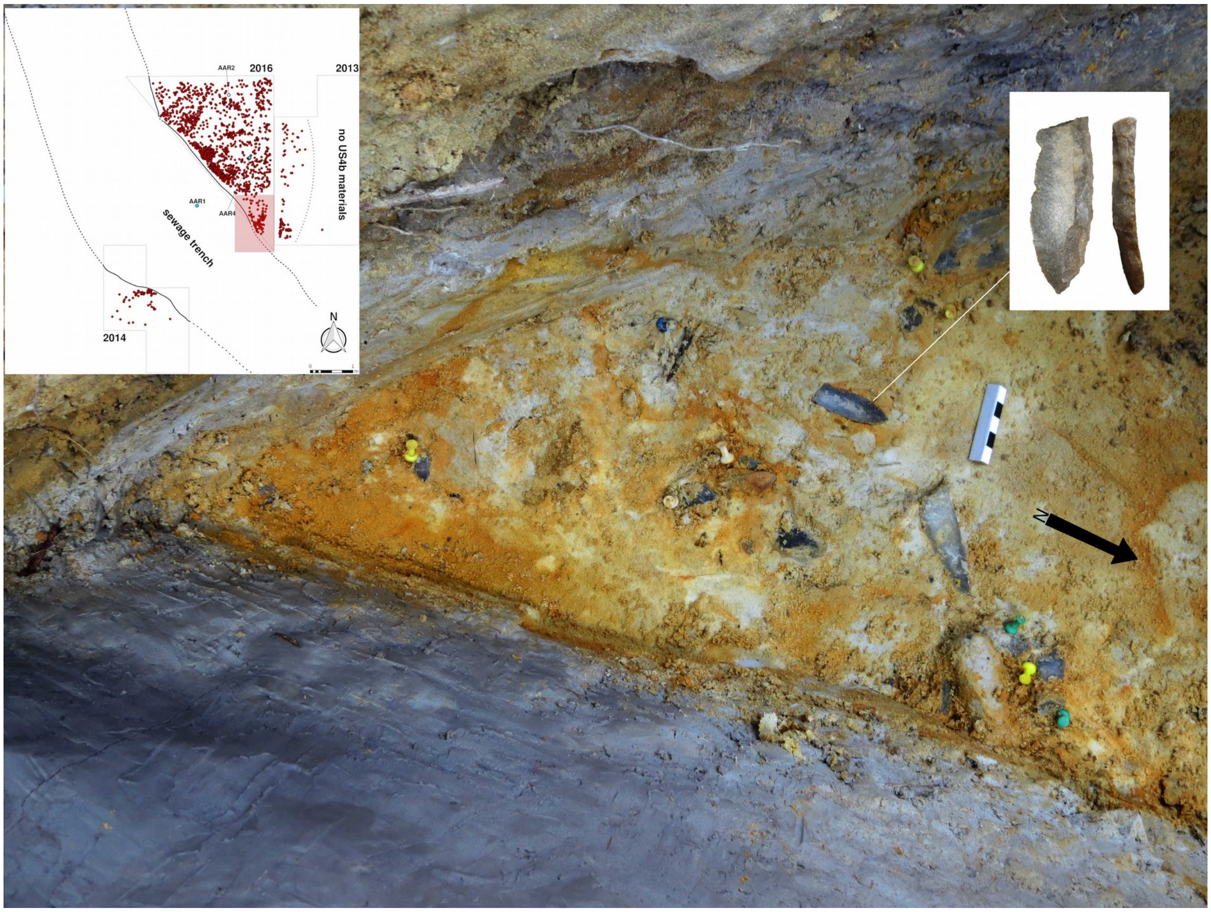

Fig. S10. Concentration of materials in US4b (Area 3) including one proximal fragment of a Châtelperronian point.

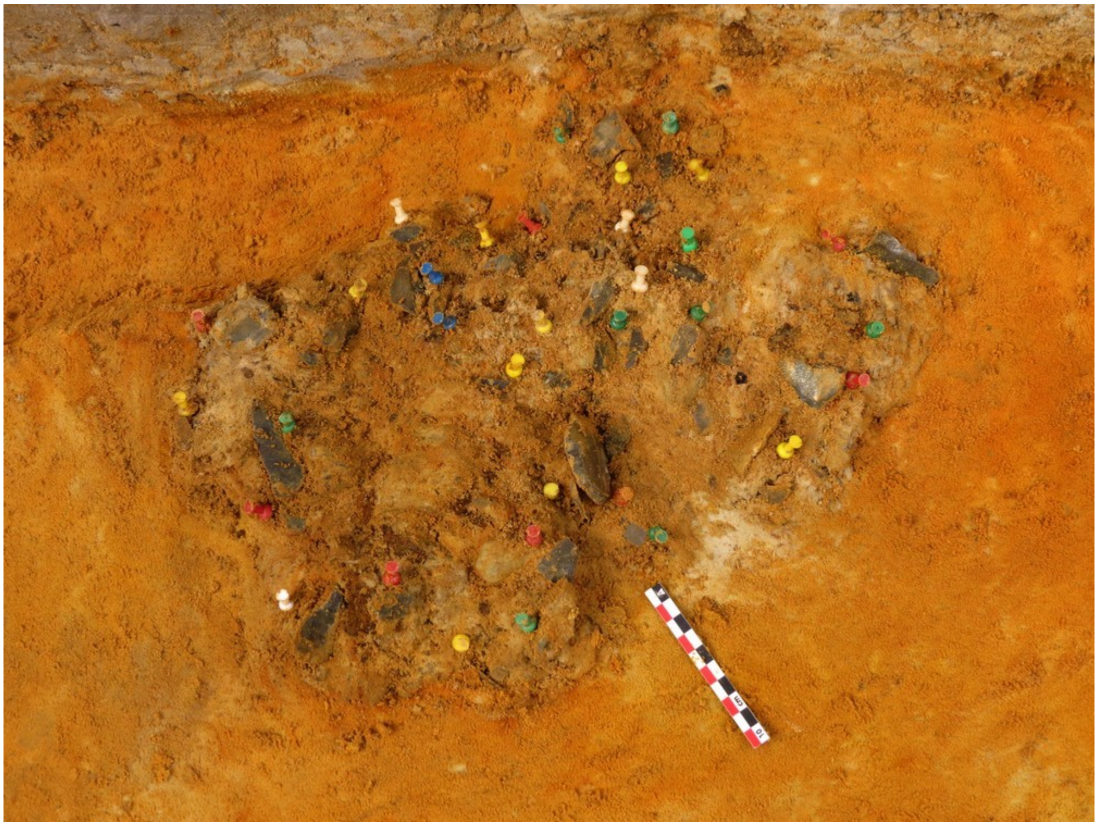

Fig. S11. Concentration of materials in US4b (Area 3).

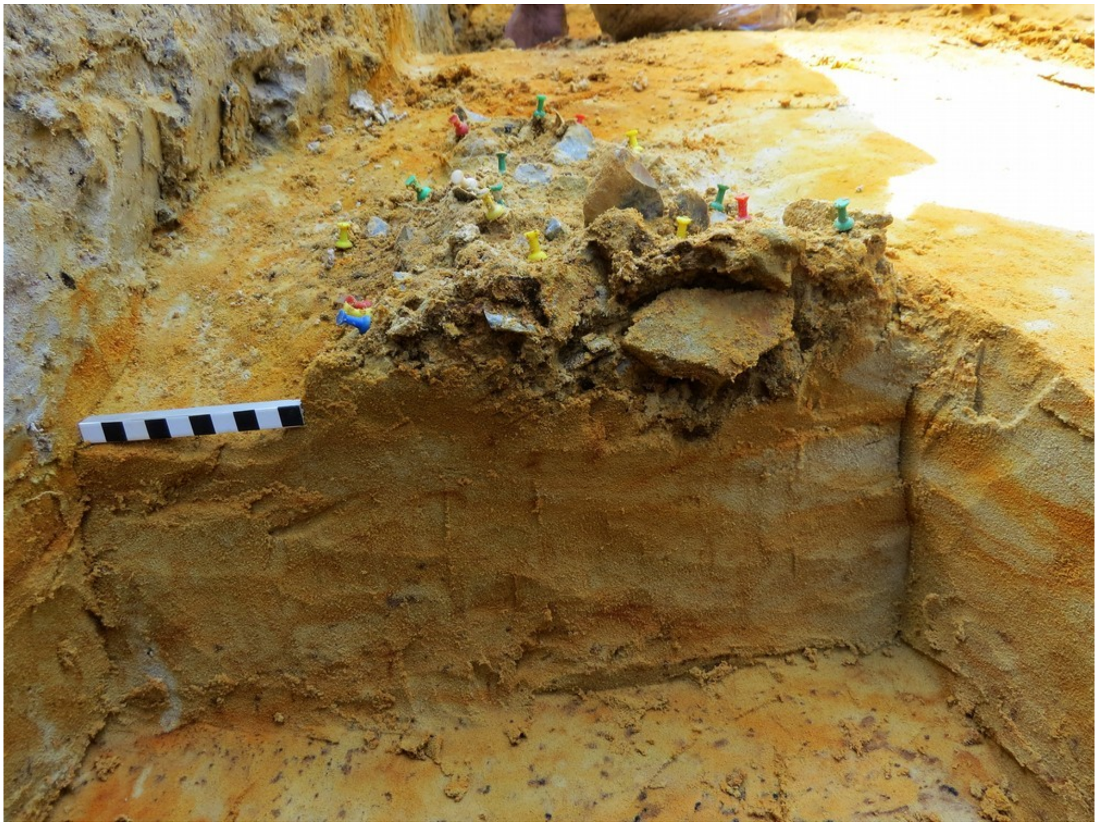

Fig. S12. Section of the concentration shown in Figure S11 (Area 3).

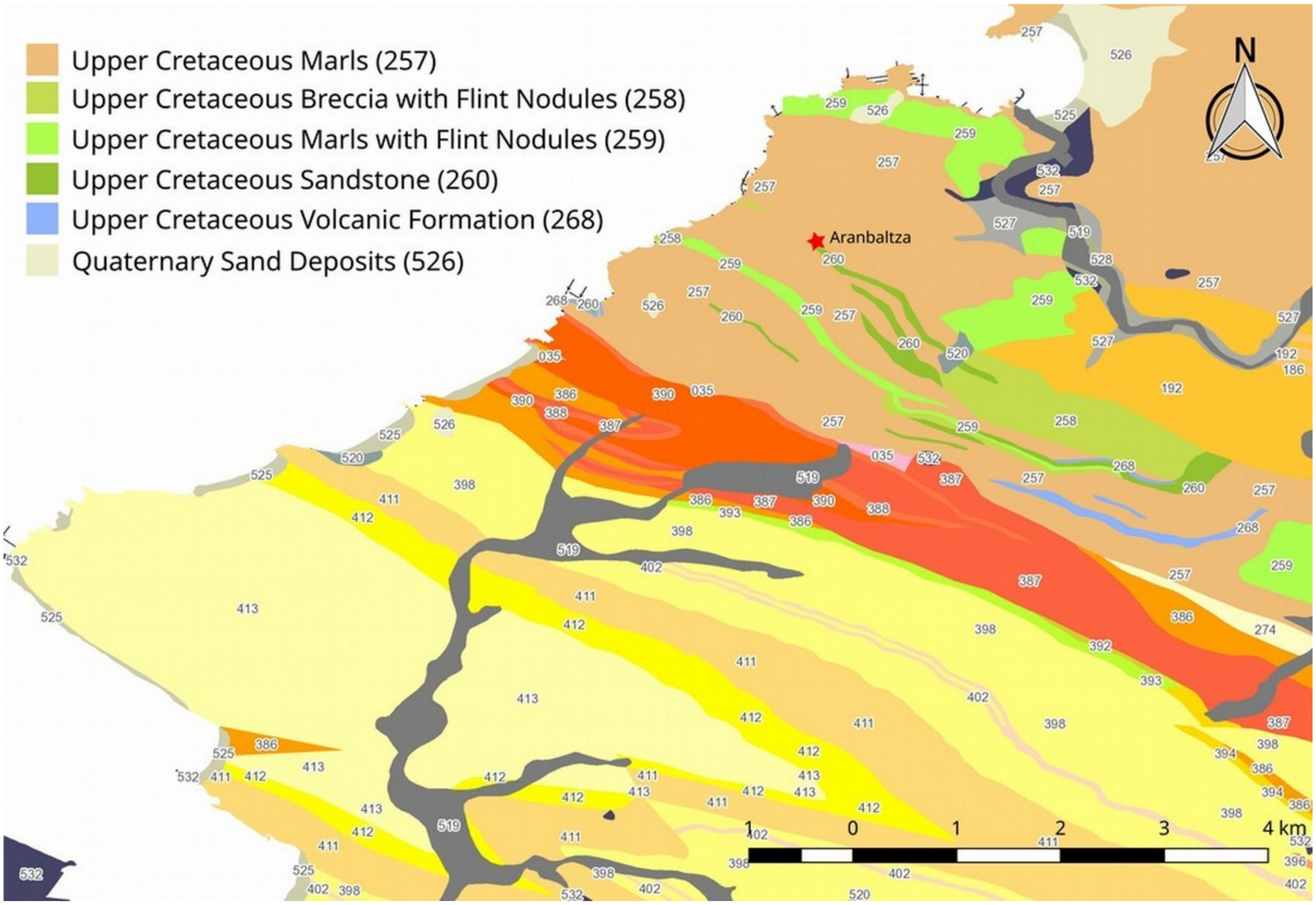

Fig. S13. Lithological map of the Uribe Kosta area (EVE- https://agserver.eve.eus/agserver/services/Geologico/j_Geologico_25000_WGS84/MapServer/WMSServer?request=GetCapabilities&service=WMS).

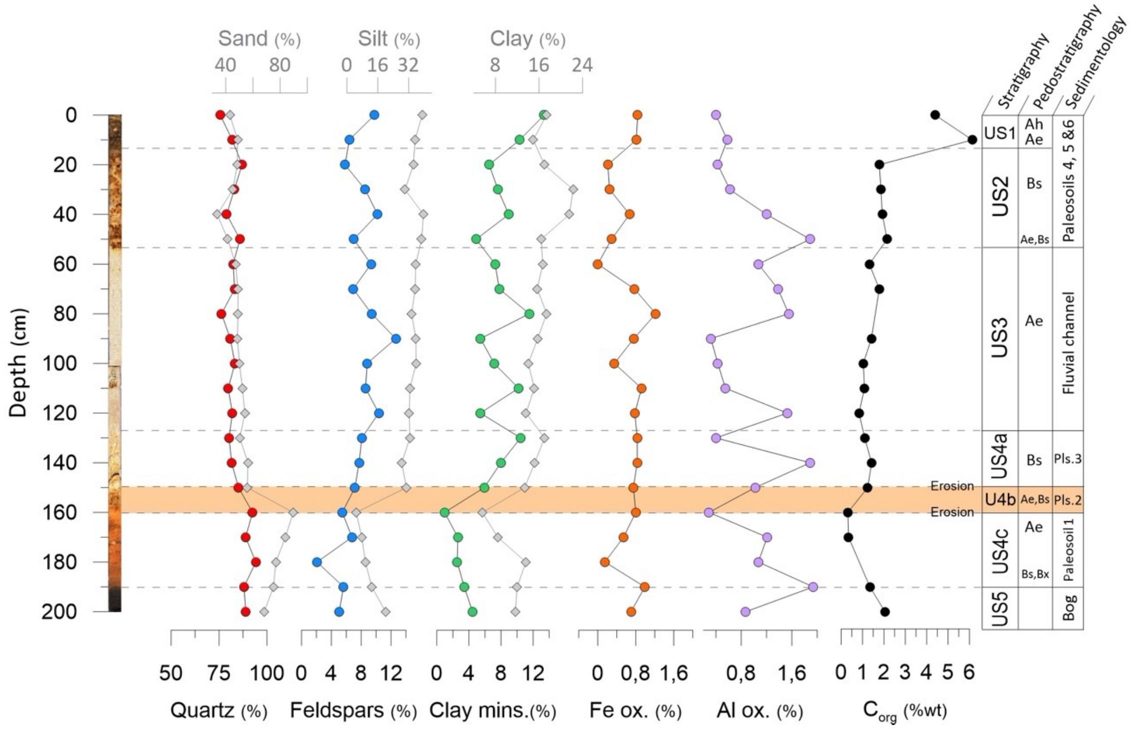

Fig. S12. Mineralogy, granulometry and organic carbon content graphics for the Aranbaltza II sedimentary sequence from AAR4 core. Defined pedo/lithostratigraphic units and their sedimentary interpretation are also represented.

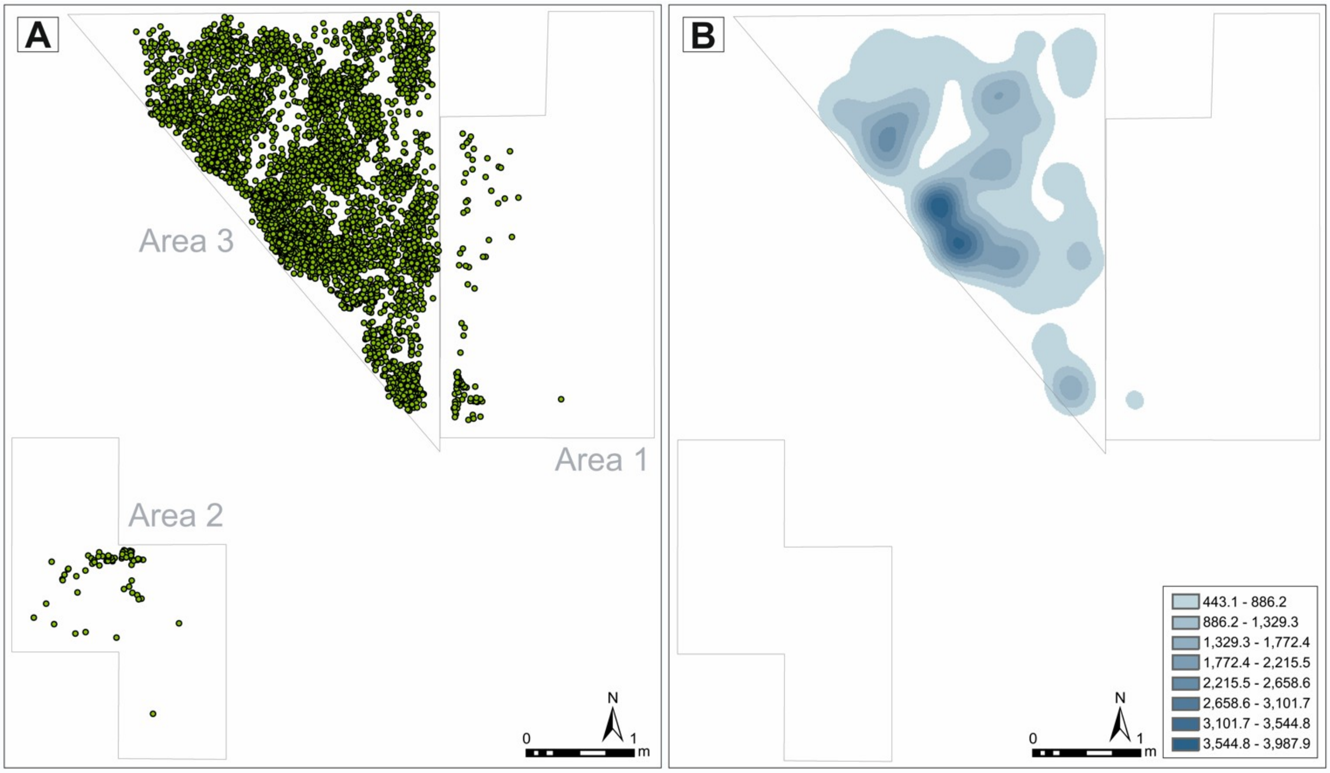

Fig. S13. A) Projection of all the materials excavated in level US4b of Aranbaltza II (n=5414); B) Kernel density analysis.

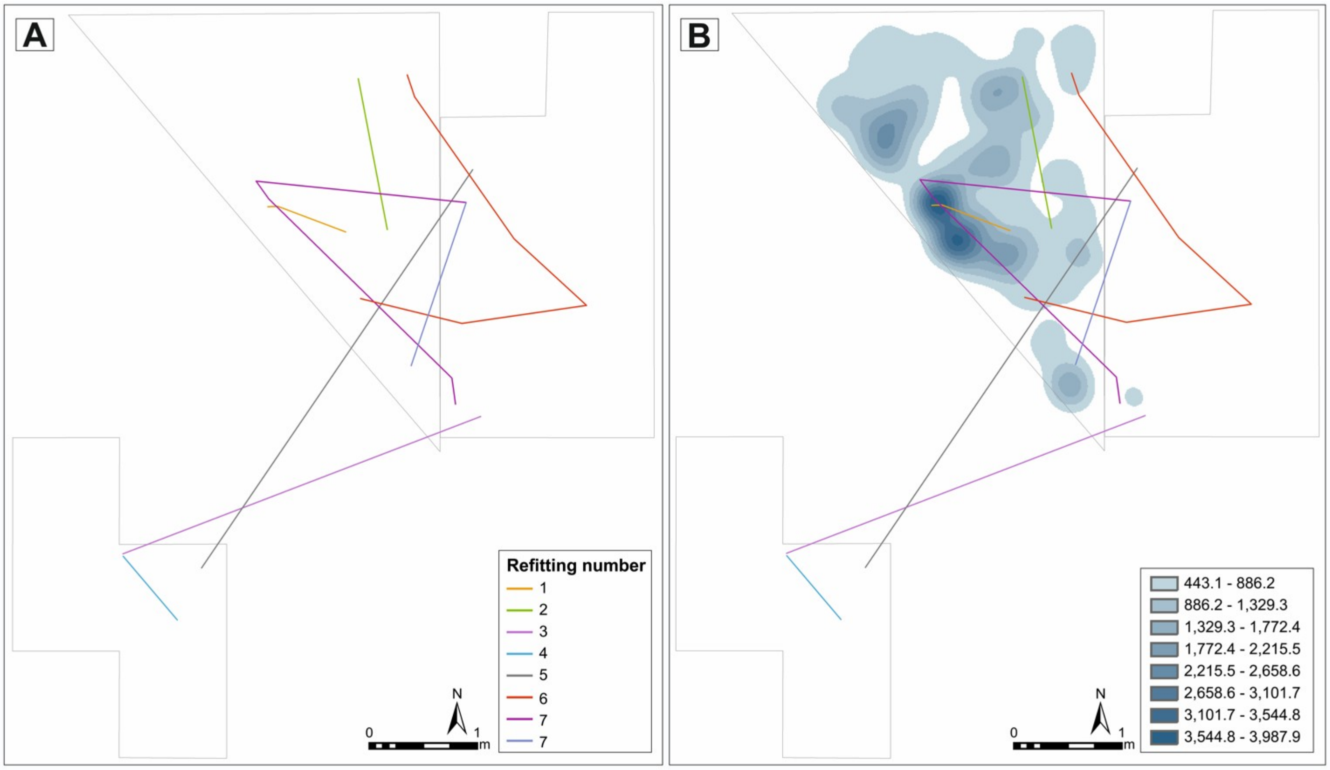

Fig. S14. A) Refitting lines for Aranbaltza II; 2) Comparison between the dispersion of the refitting lines and the kernel density analysis of the lithic pieces at Aranbaltza II.

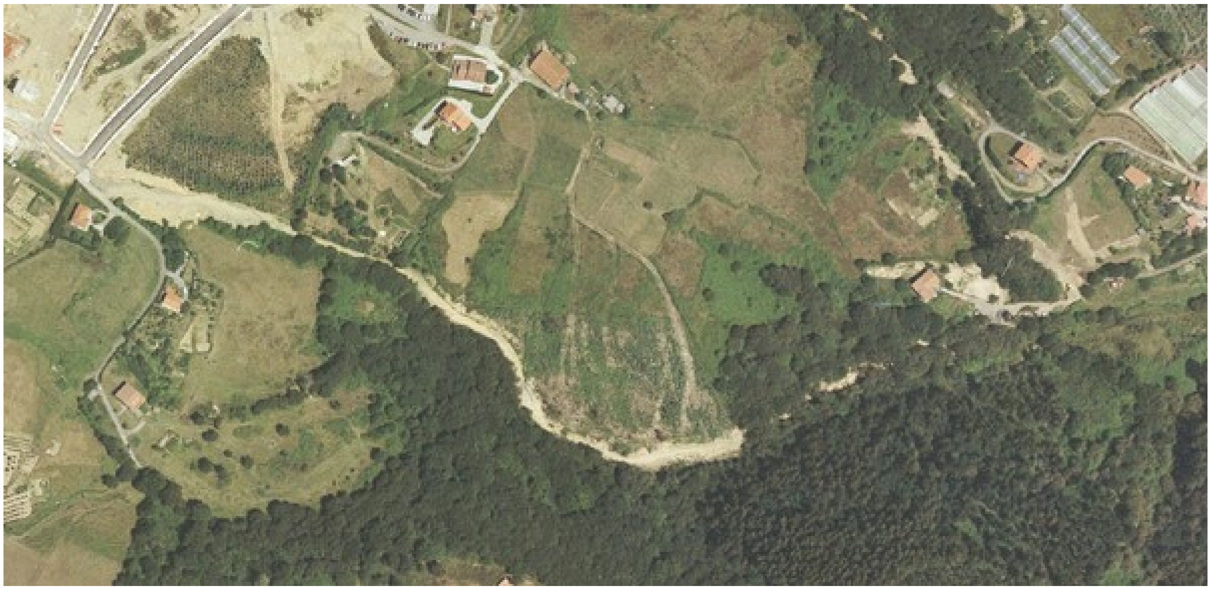

Fig. S15. Aranbaltza and Ollagorta during the excavation of the sewage trench in 2004. Photo from https://www.geo.euskadi.eus.

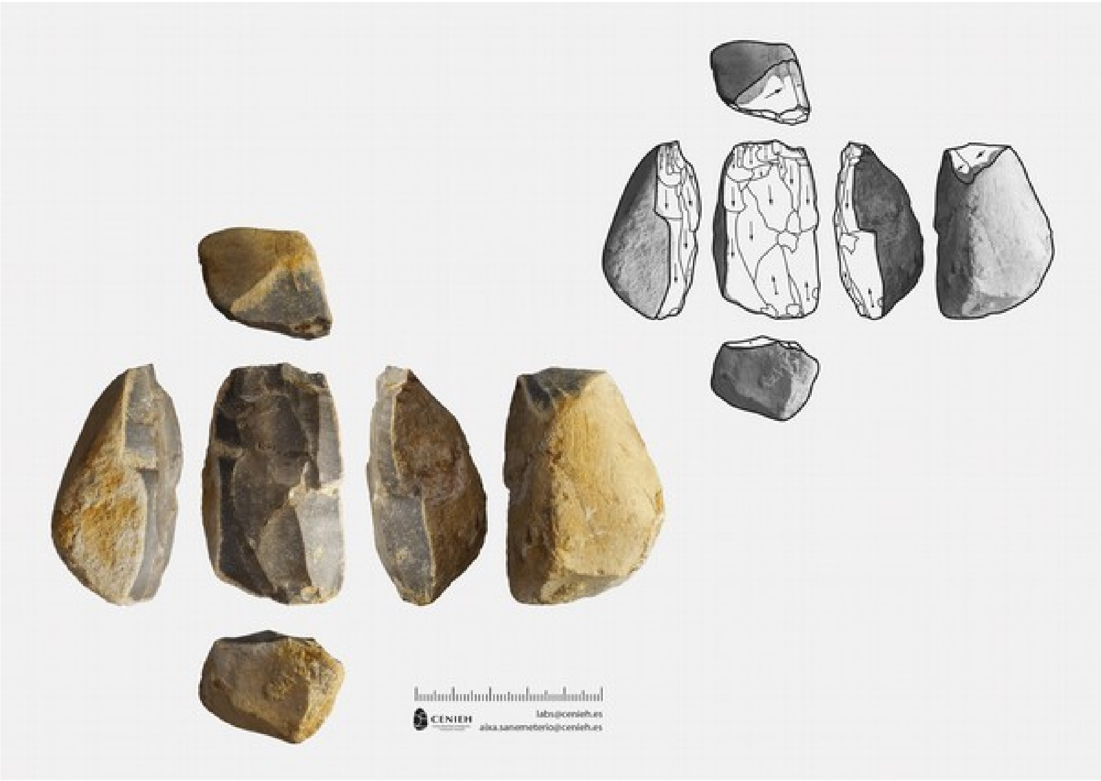

Fig. S16. Bidirectional (*bipolaire décalé*) blade core.

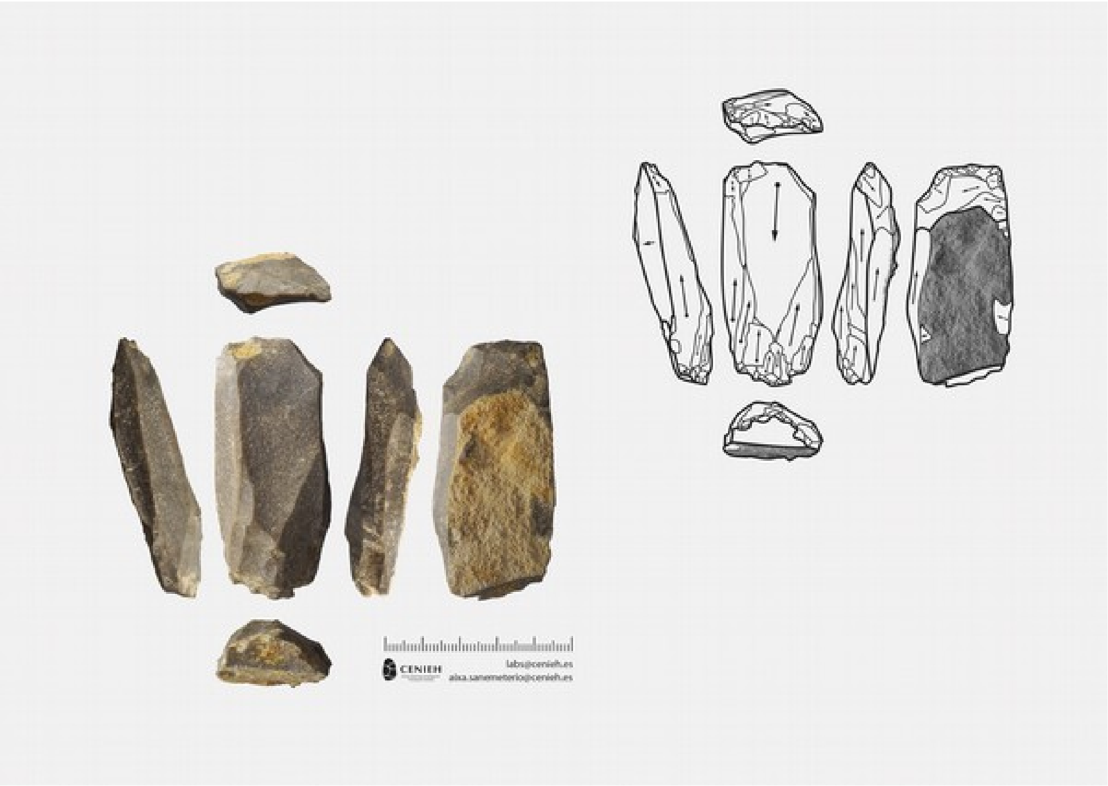

Fig. S17. Bidirectional (*bipolaire décalé*) blade core.

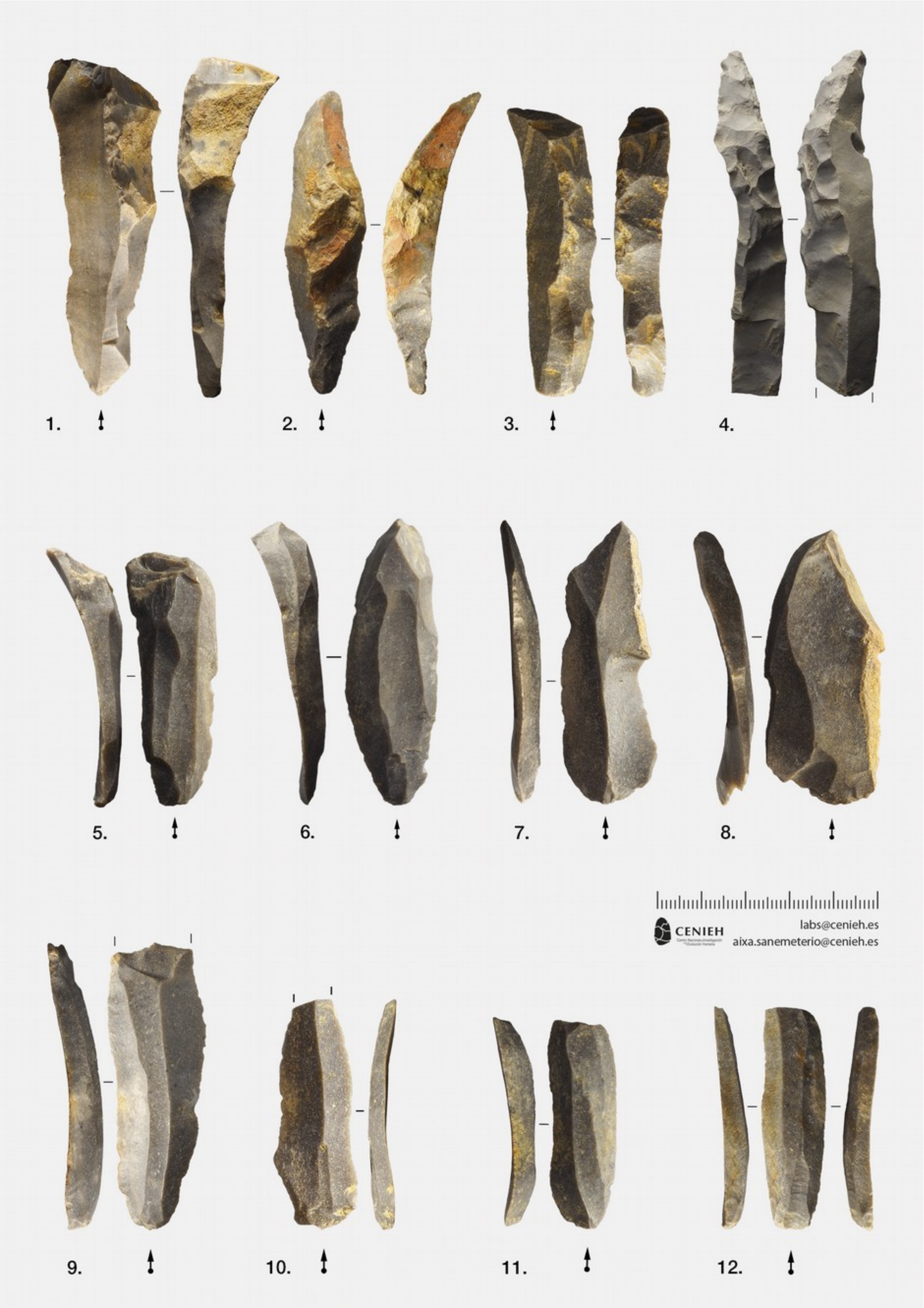

Fig. S18. 1-4: crested blades; 5-6: overshot blades; 7-8: flank blades; 9-12: blade fragments

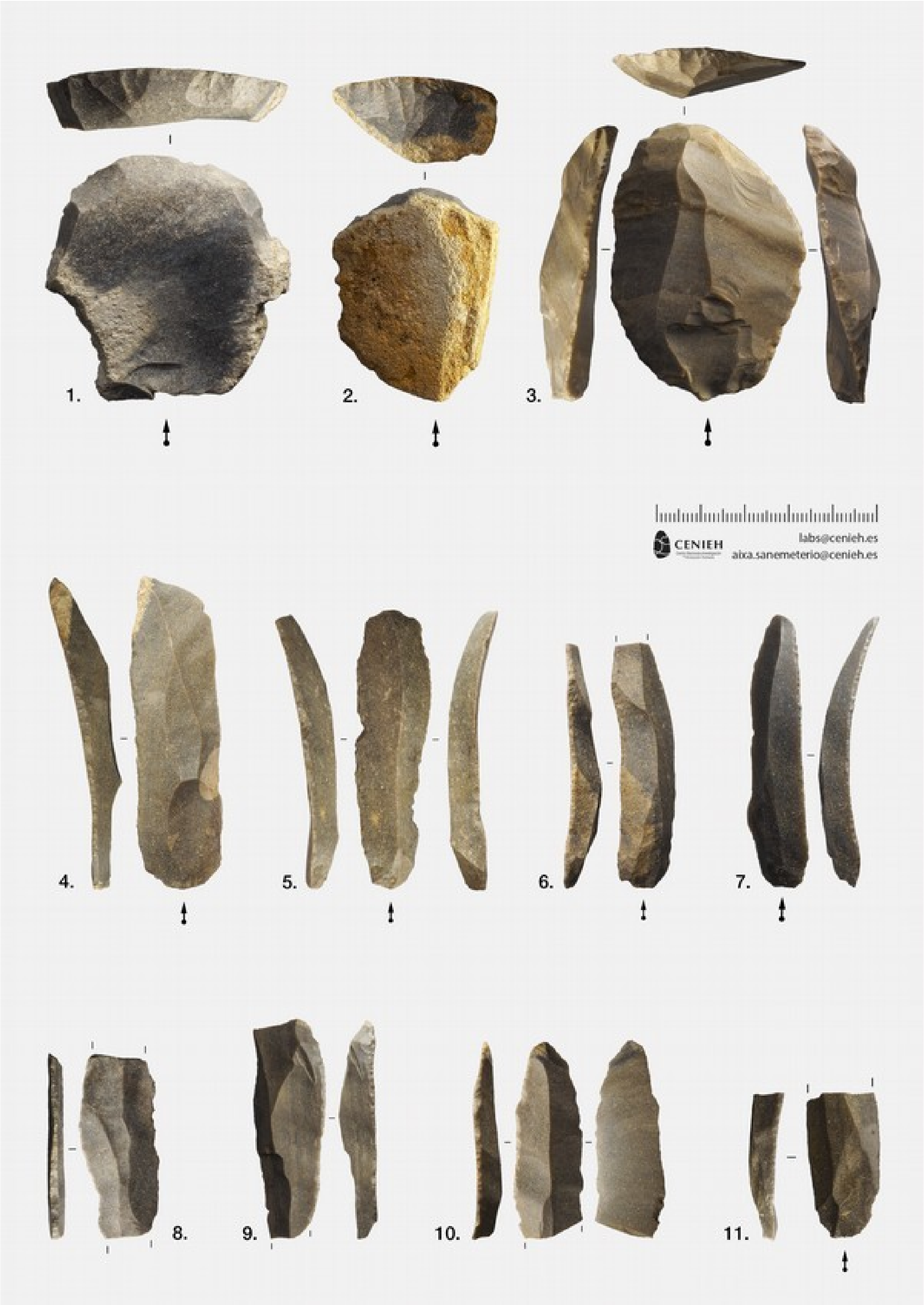

Fig. S19. 1-3: endscrapers on flakes; 4-11: marginally backed blades.

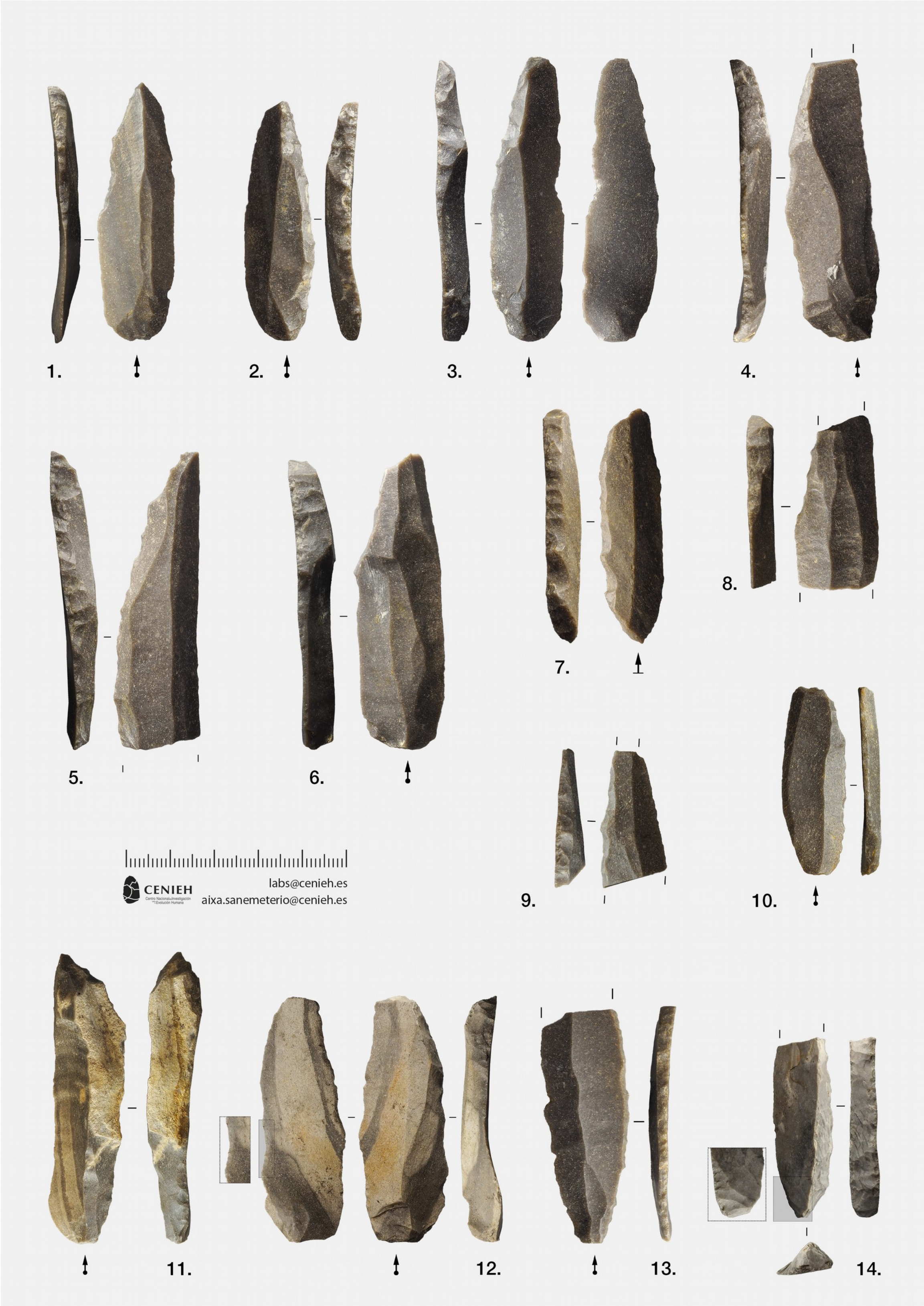

Fig. S20. 9-10: Châtelperronian points and point fragments; 11-14: backed blades.

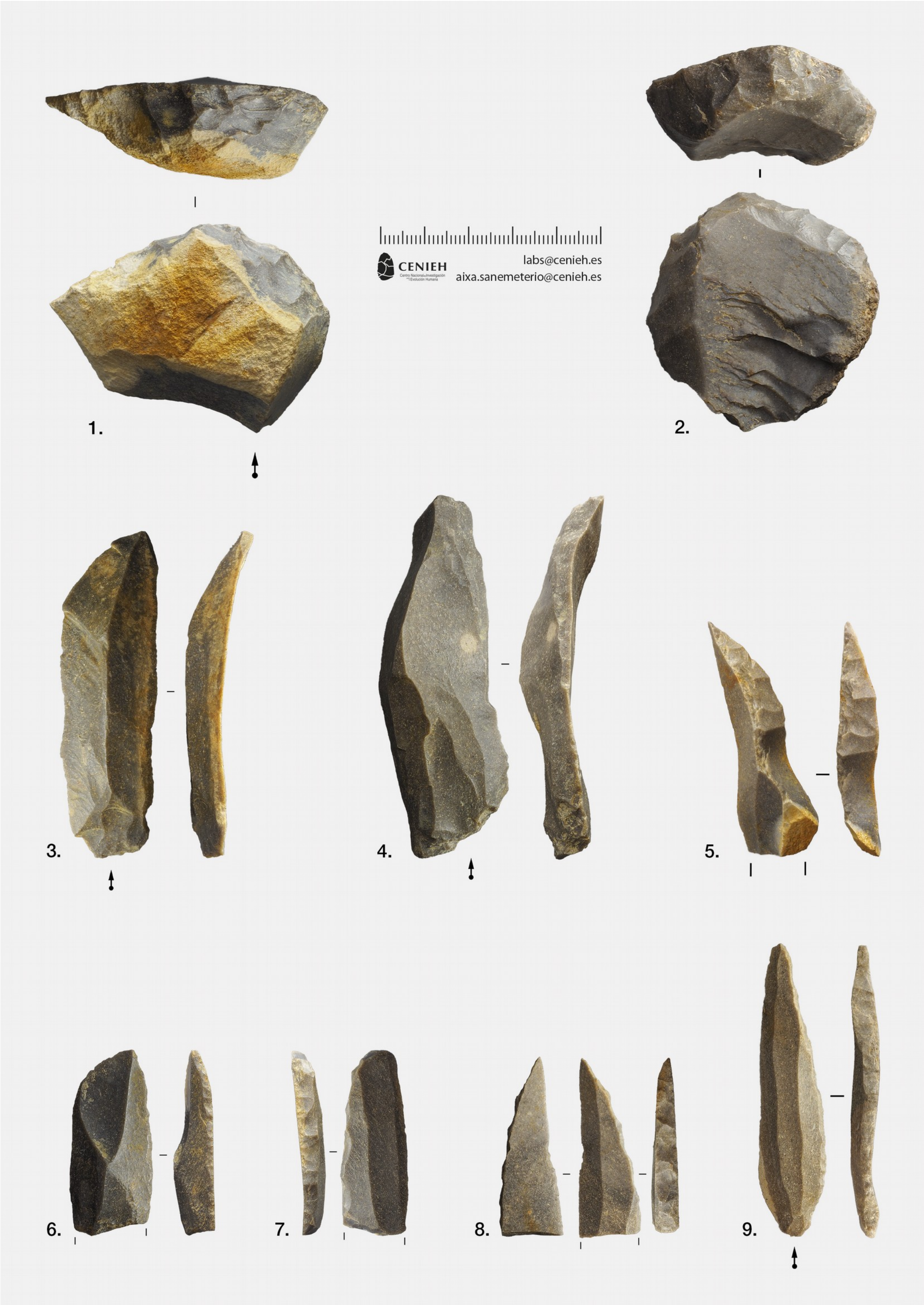

Fig. S21. 1-2: endscrapers on flakes; 3-4,6,7: backed blades: 5,8-9: backed points.

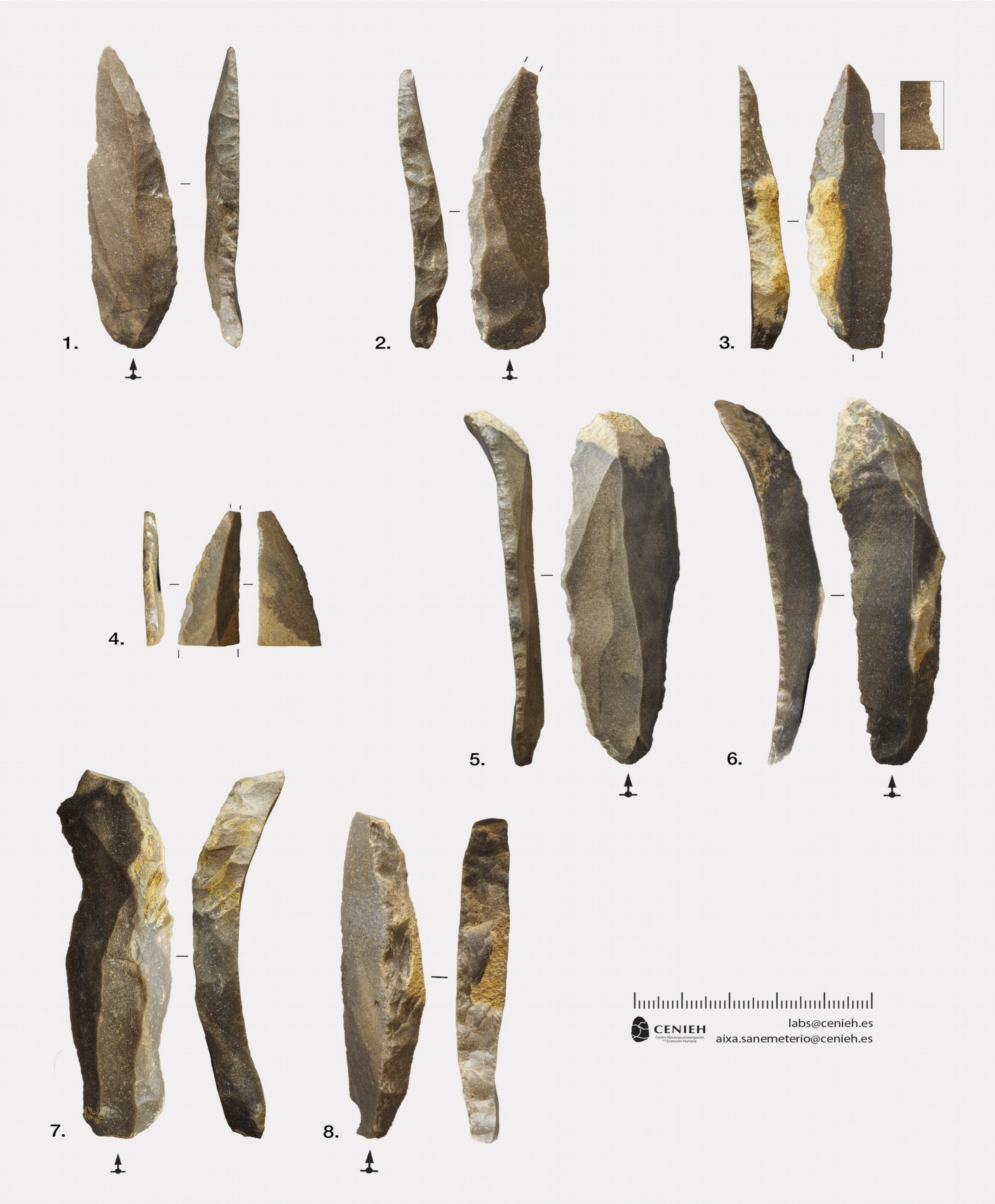

Fig. S22. Aranbaltzabarrena materials: 1-4: Châtelperronian points; 5-6: backed blades; 7-8: crested blades.

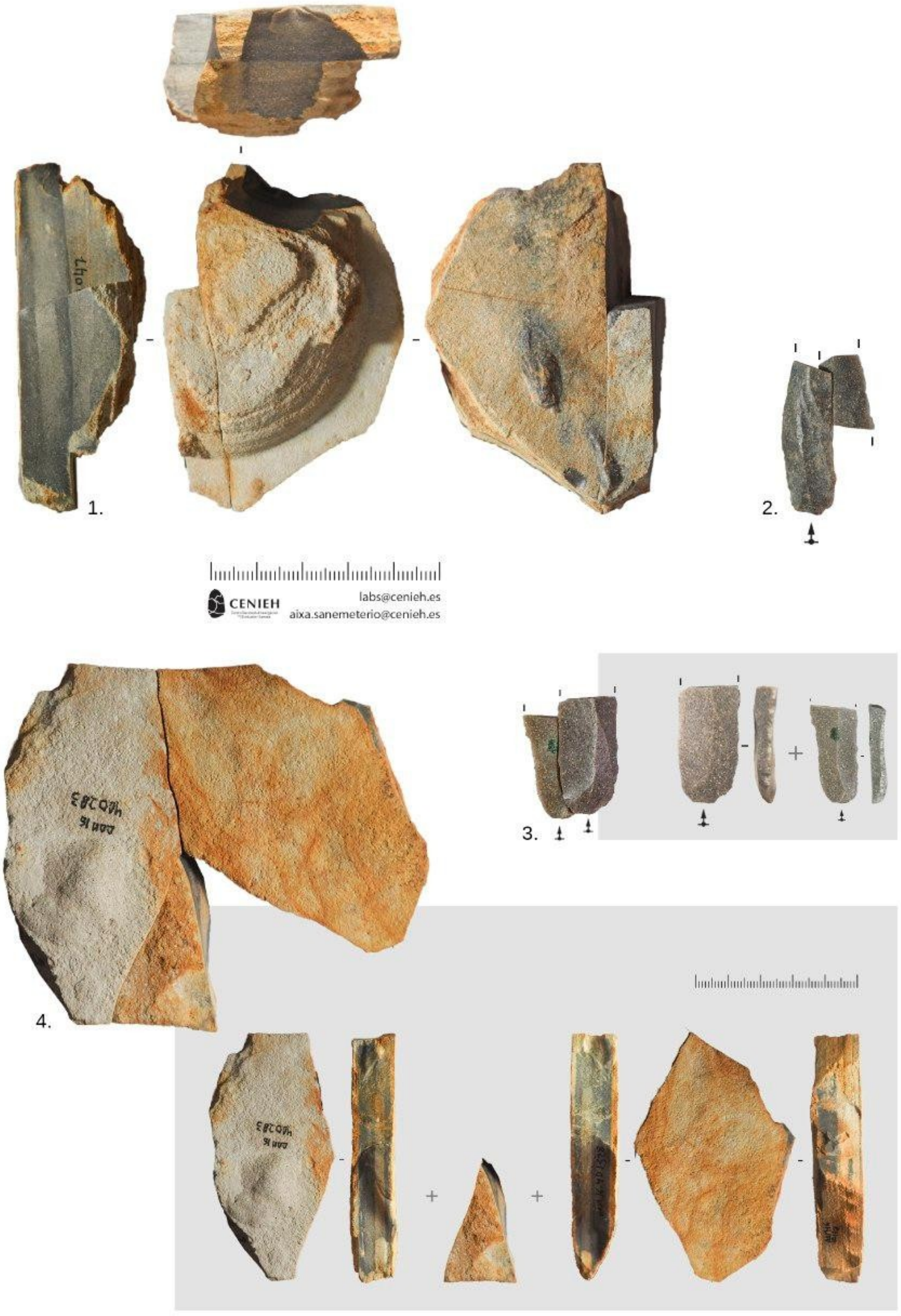

Fig. S23. Flint refittings, including two slabs (1, 4) and backed blades (2-3).

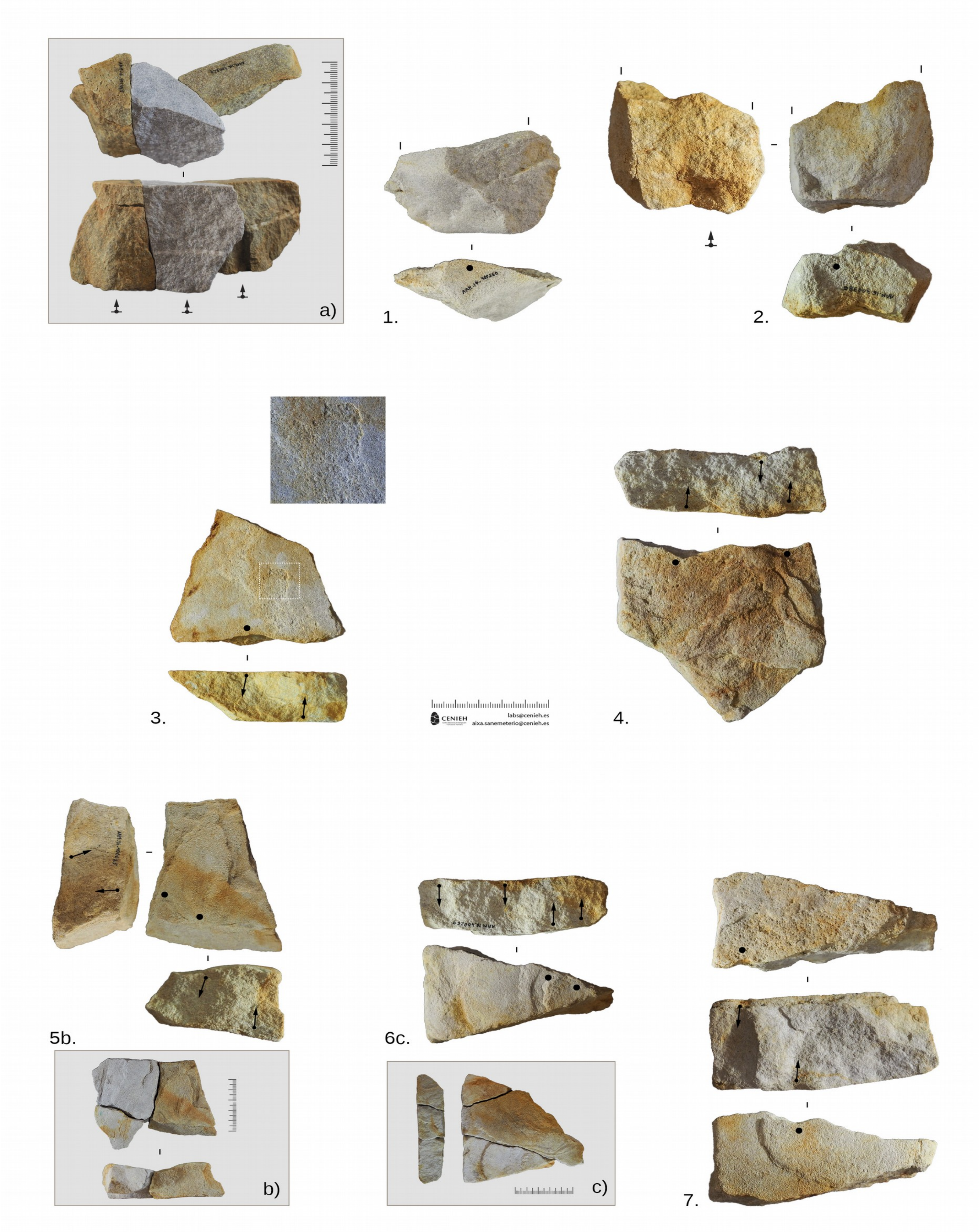

Fig. S24. Sandstone slab fragments and flakes, including three refitting series (a, b and c).

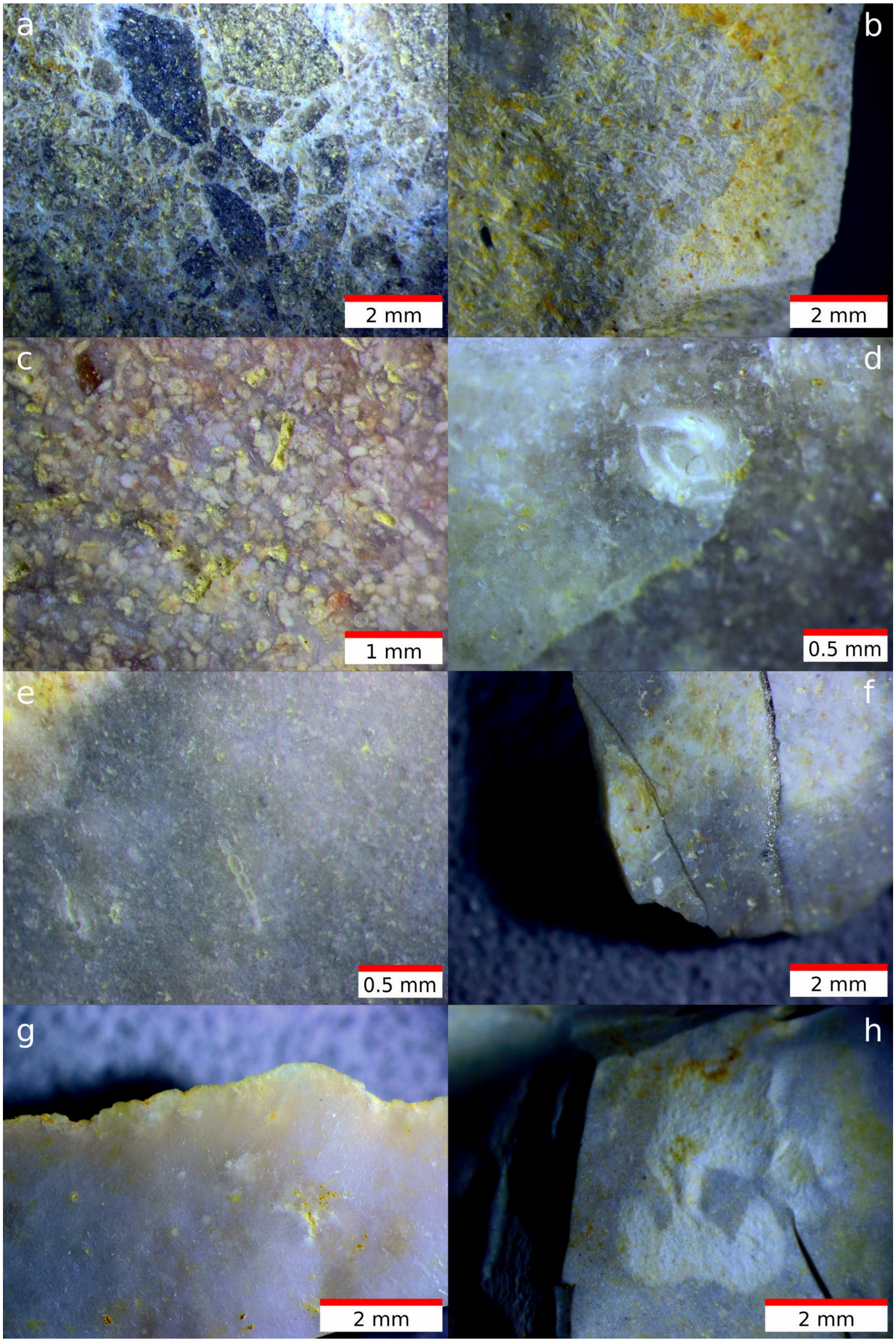

Fig. S25. Macroscopic aspect of the different flint types identified. a) Flysch flint silcrete; b) Flysch flint with silicified sponge spicules; c) wackestone flint, with very good sorting of grains; d) flint with miliolids; e-f) flint with external marine platform fossils; g) internal marine platform translucent flint; h) Salies-de-Béarn flint with bioturbations.

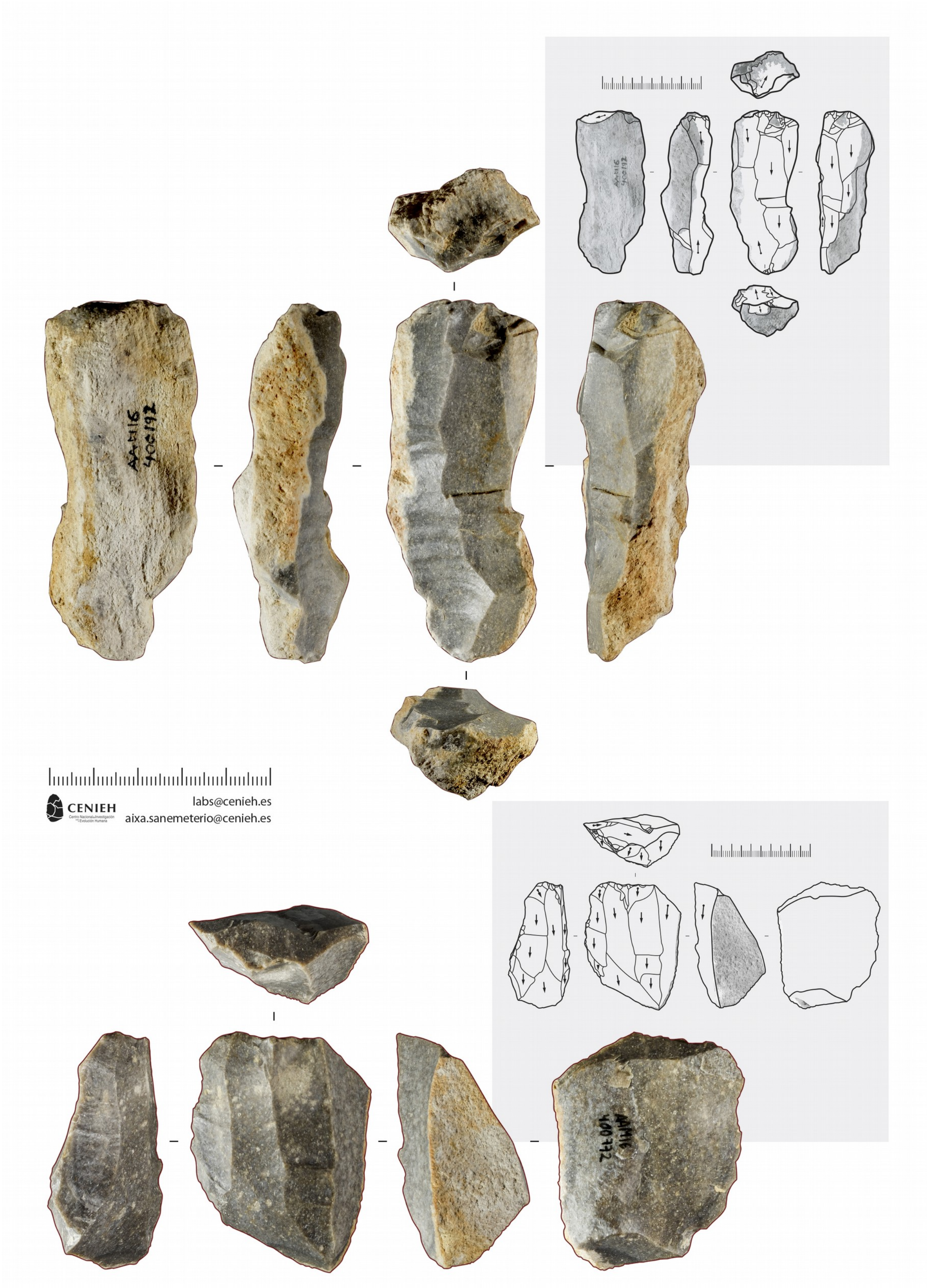

Fig. S26. 1: Bidirectional (*bipolaire décalé*) blade core (3D model available in https://doi.org/10.6084/m9.figshare.17871749). 2: Unidirectional blade core.

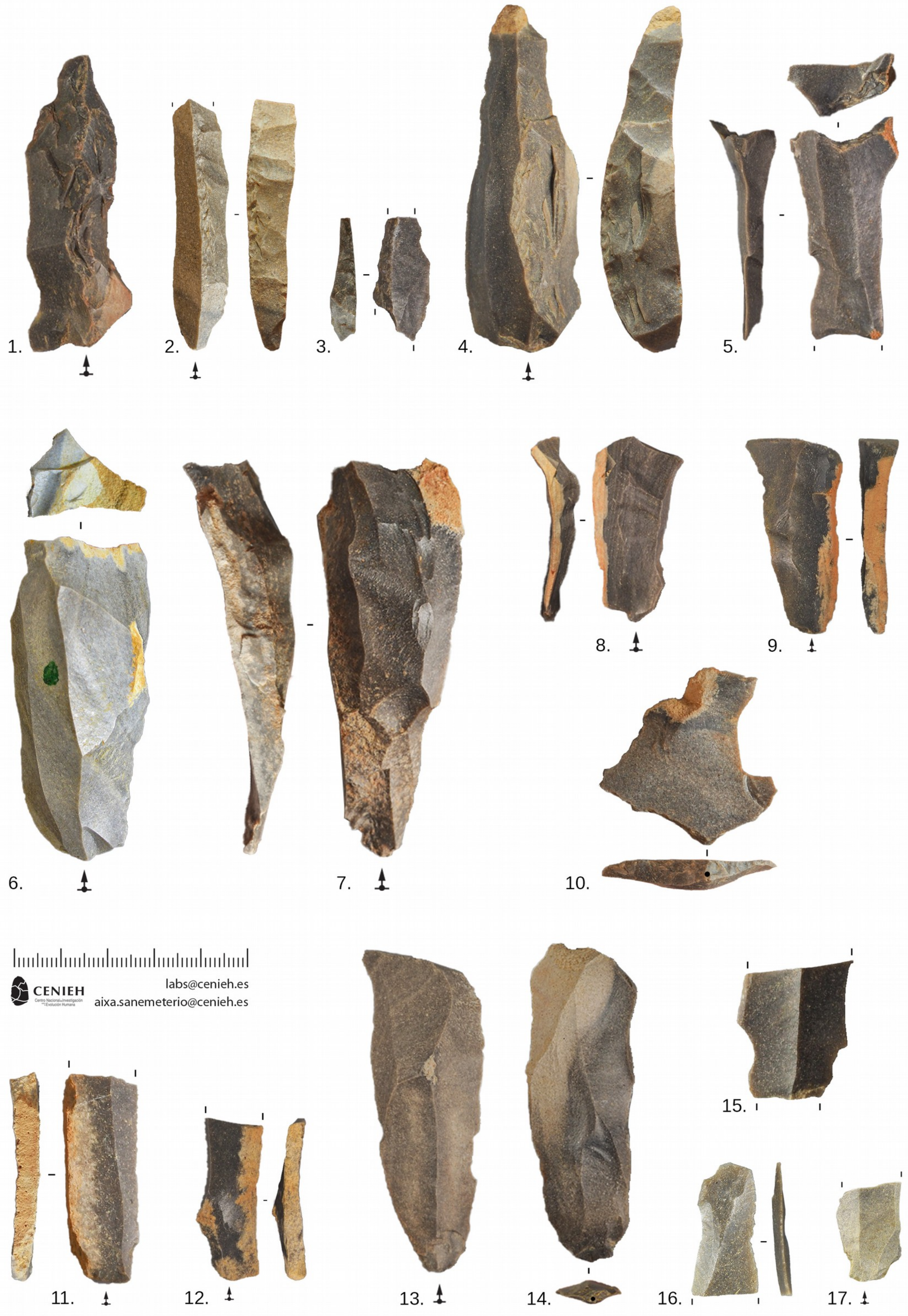

Fig. S27. Blade core maintenance products: 1-4: crested blades; 5-9: overshot blades from bidirectional cores; 10: platform rejuvenation flake: 11-12: cortical flank blades; 13-14: bidirectional blades; 15-17: blade fragments.

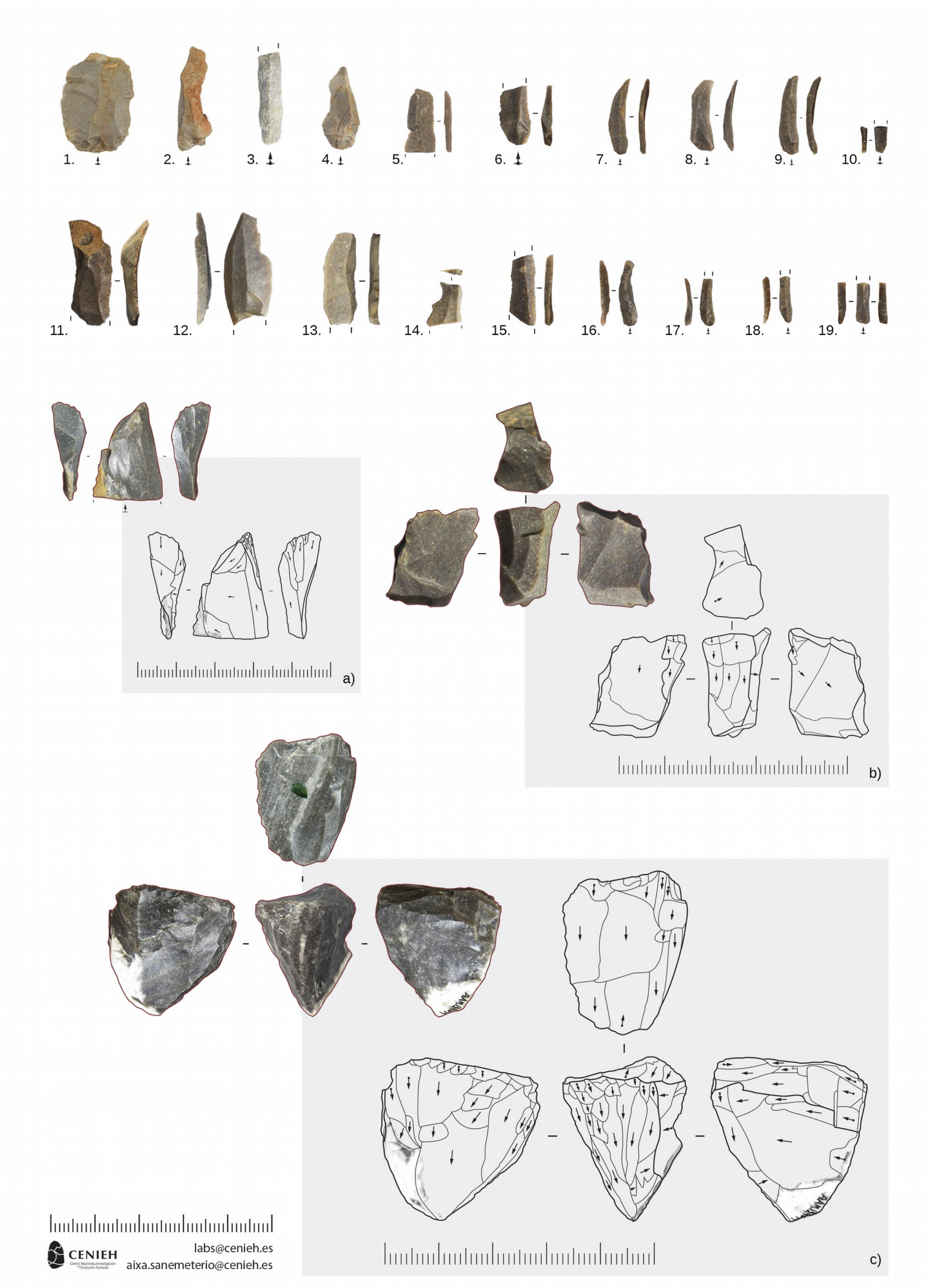

Fig. S28. 1-19: bladelet products; a-c: bladelet cores.

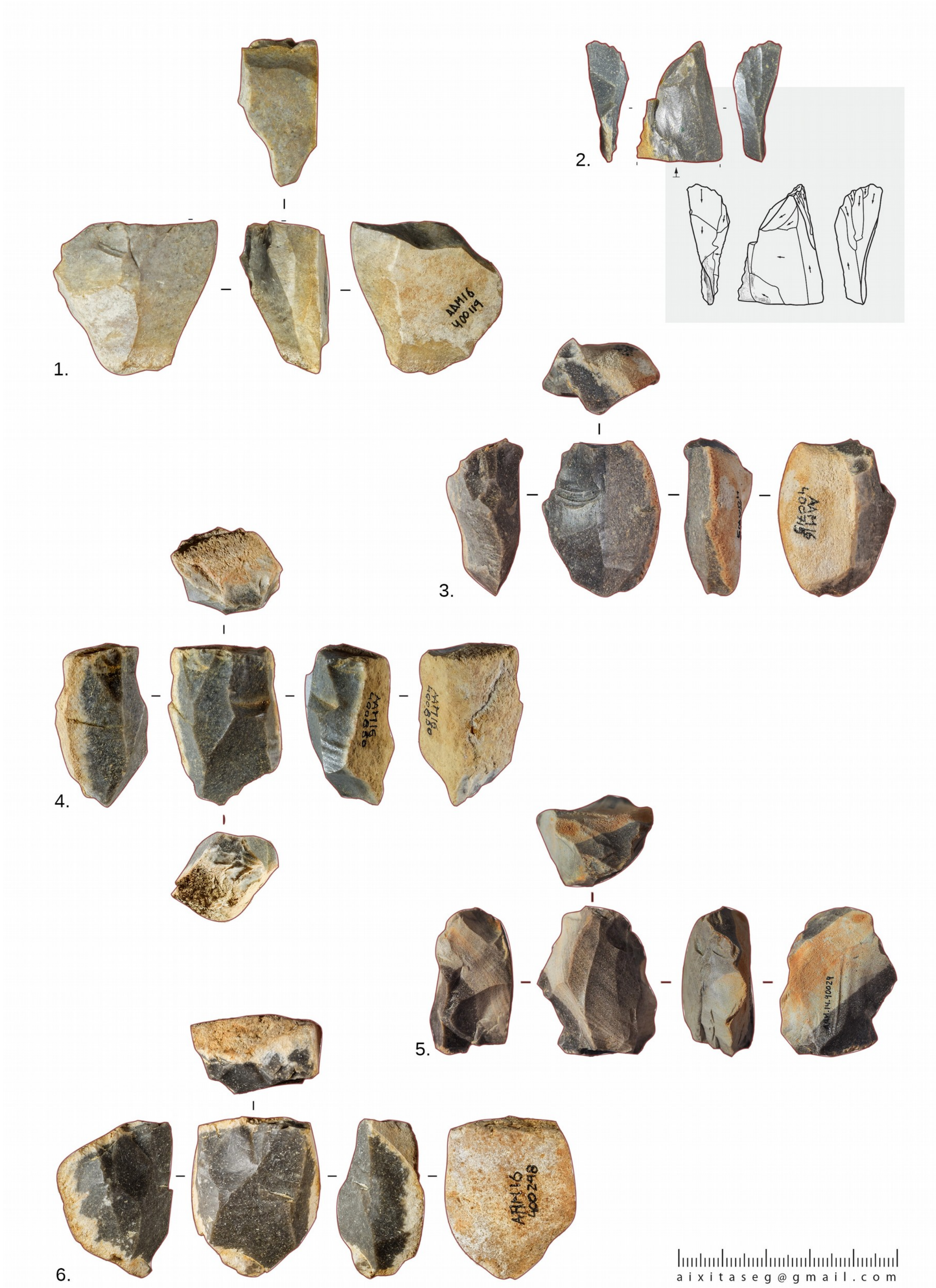

Fig. S29. 1-6: Bladelet cores.

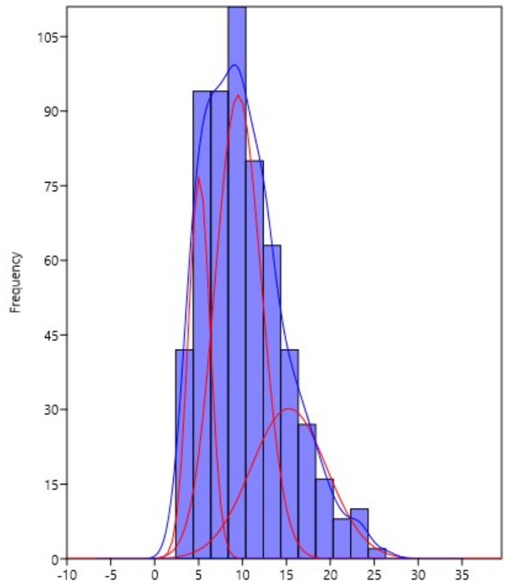

Fig. S30. Mixture analysis of the blade widths distribution. The histogram shows the actual distribution, the blue line is the kernel grouping, and the red curves are the distributions of the three groups identified (log lk.hood: -1153; Akaike IC: 2318). Analysis conducted using the software Past [138].

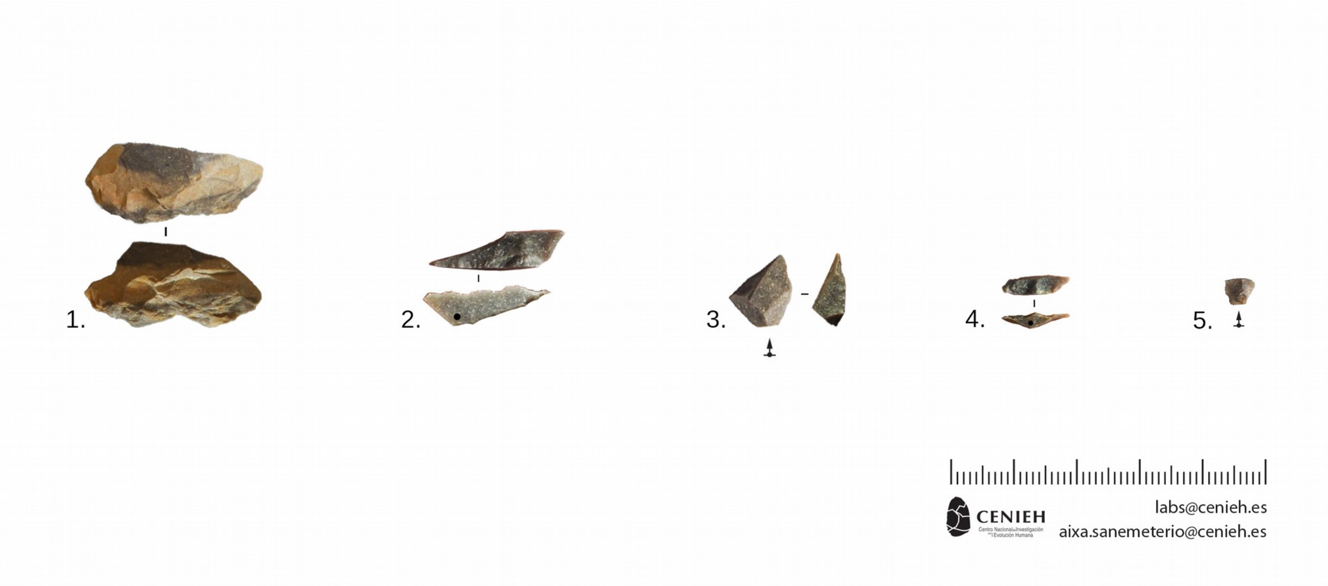

Fig. S31. Resharpening flakes originated during back configuration: numbers 2 and 3 are accidental flakes that have removed the tip of a backed point.

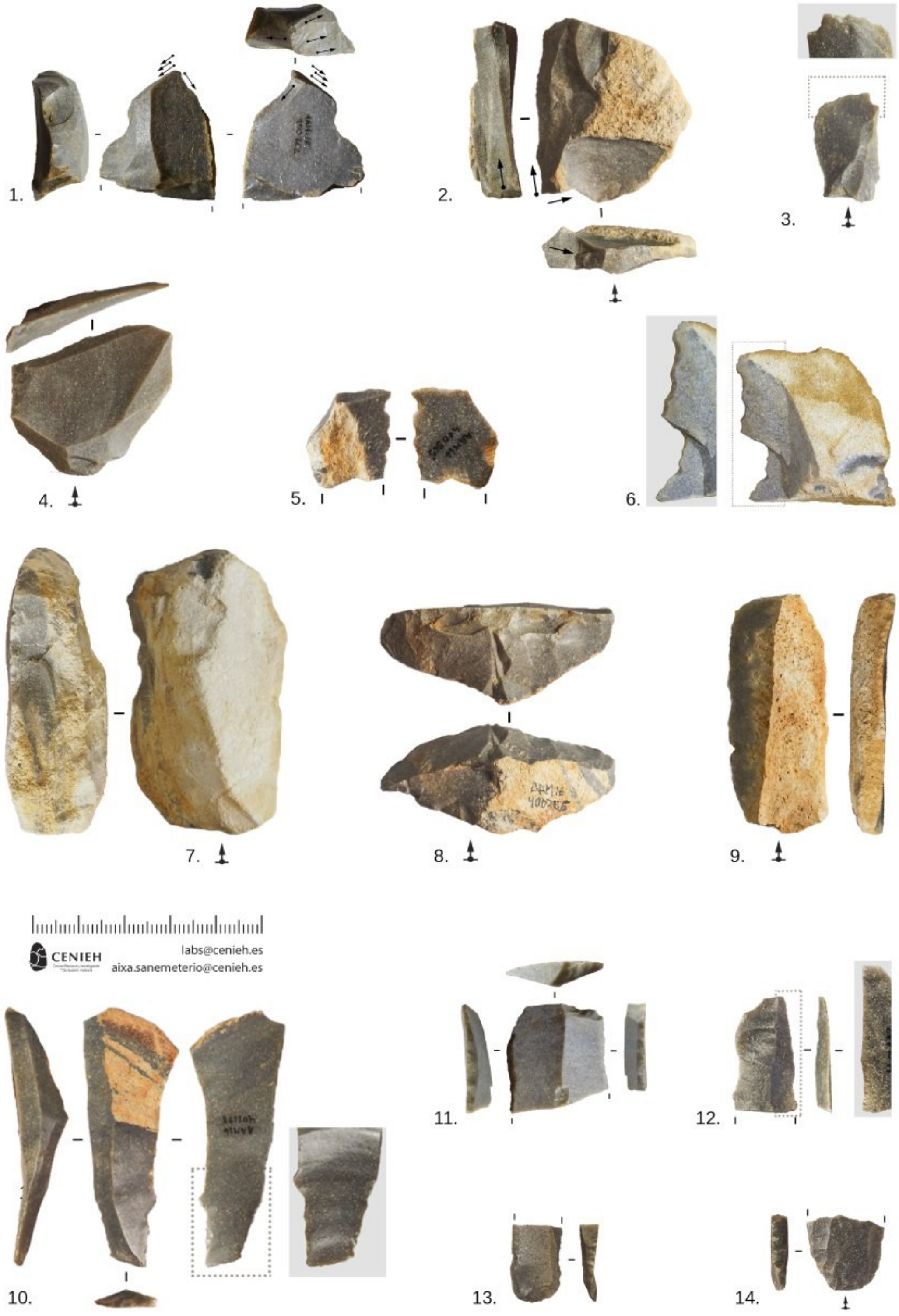

Fig. S32. Aranbaltza II-US4a retouched tools. 1-2: nucleiform burins; 3: borer; 4: marginally backed flake; 5-6: denticulated flakes; 7-8: sidescrapers; 9: partially backed blade; 10: truncated blade-borer; 11: retouched blade; 12: blade with marginal back on ventral surface; 13-14: backed blade fragments.

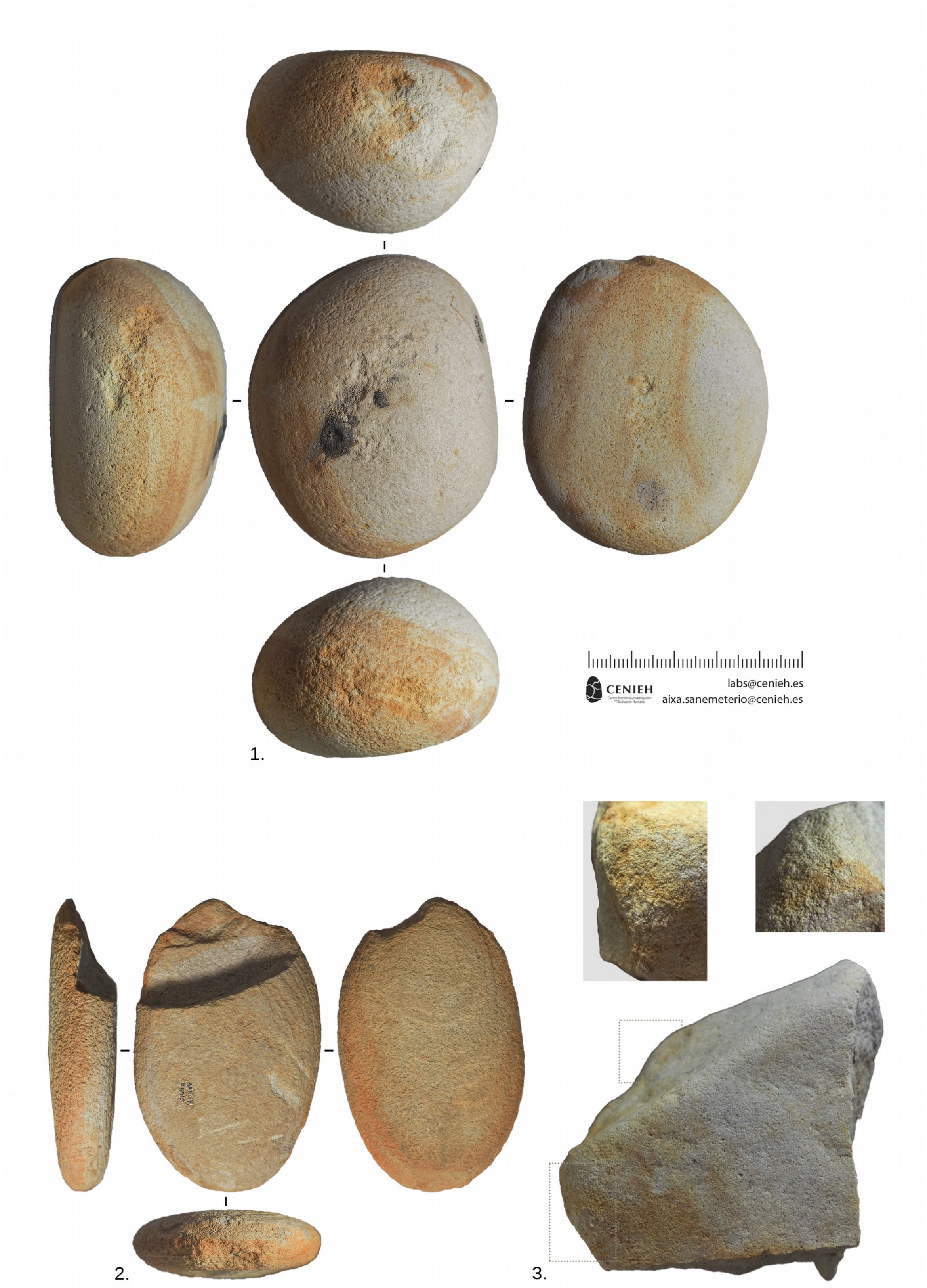

Fig. S33. 1. 1: hammerstone/anvil; 2: Hammerstone with typical hinged fracture; 3: Hammerstone.

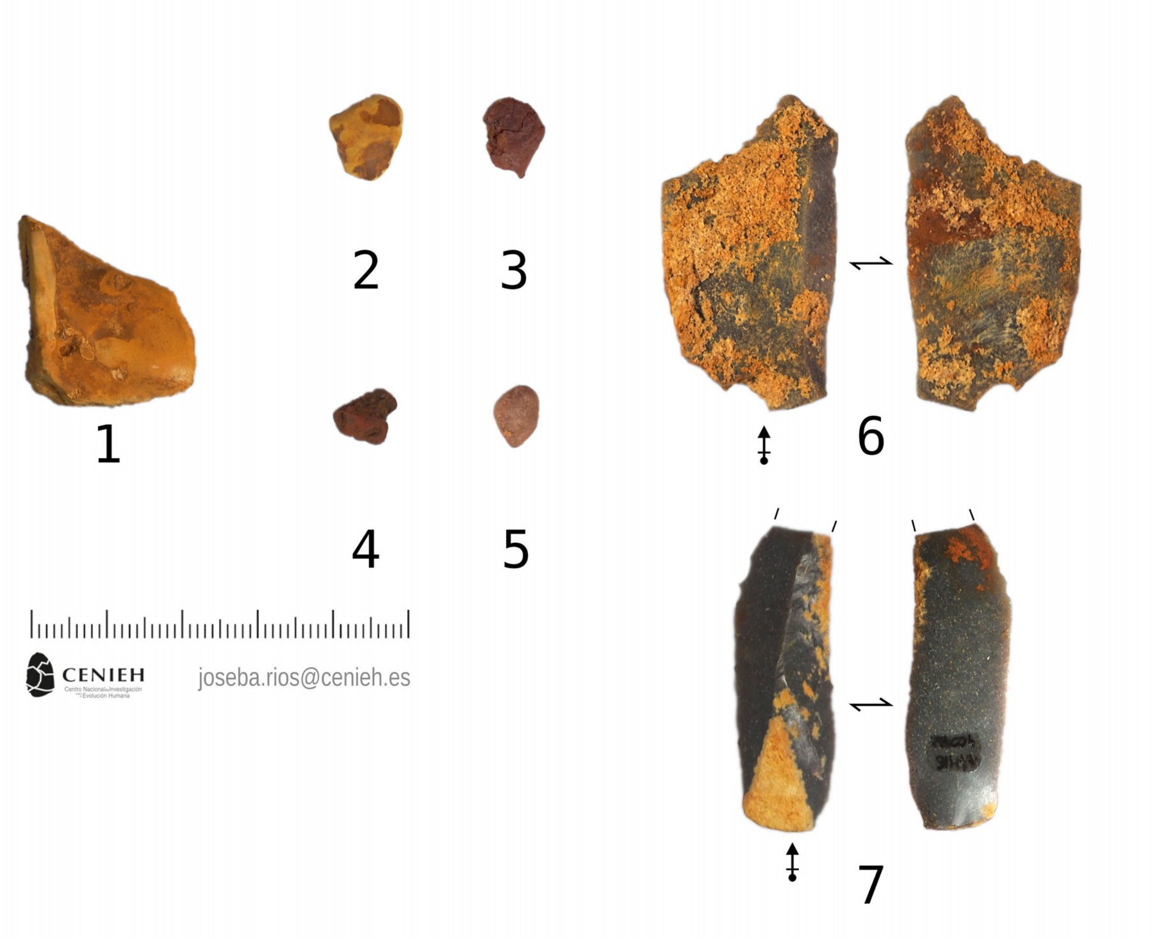

Fig. S34. Ochre fragments found in US4b (1-5), and pieces with ochre deposits (6-7).

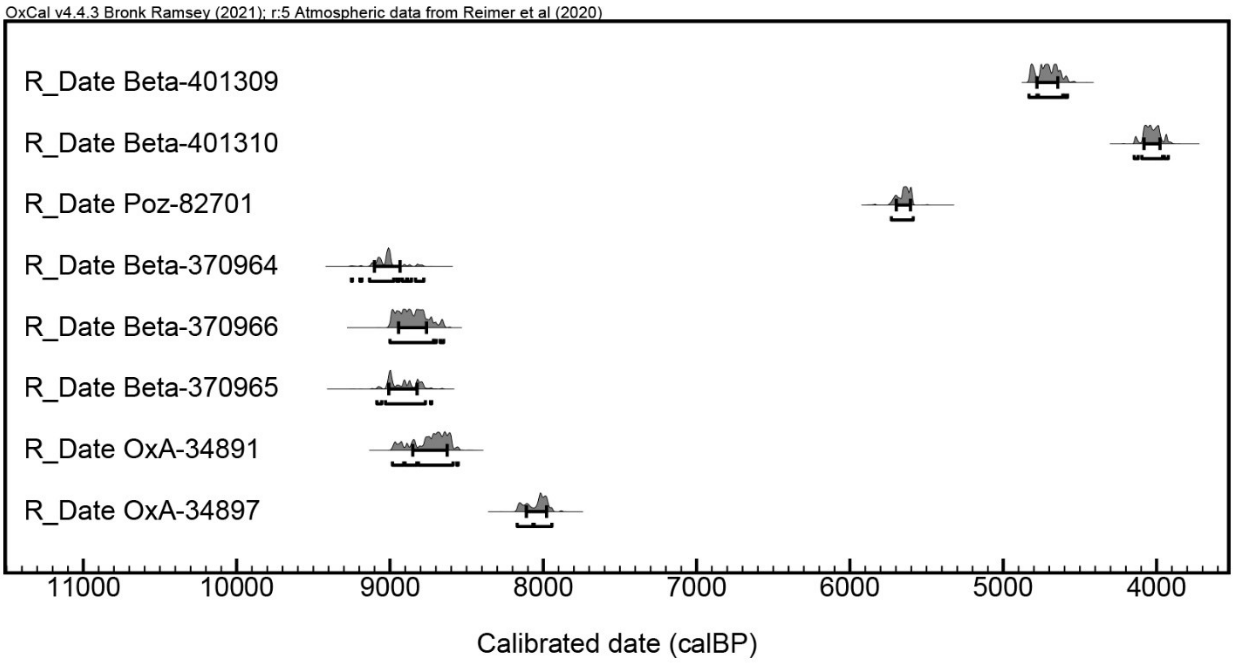

Fig. S35. Plot of dated AMS ^{14}C samples at Aranbaltza II. The ^{14}C samples have been calibrated and plotted using OxCal4.4.2 software [138] and the INTCAL20 curve [98].

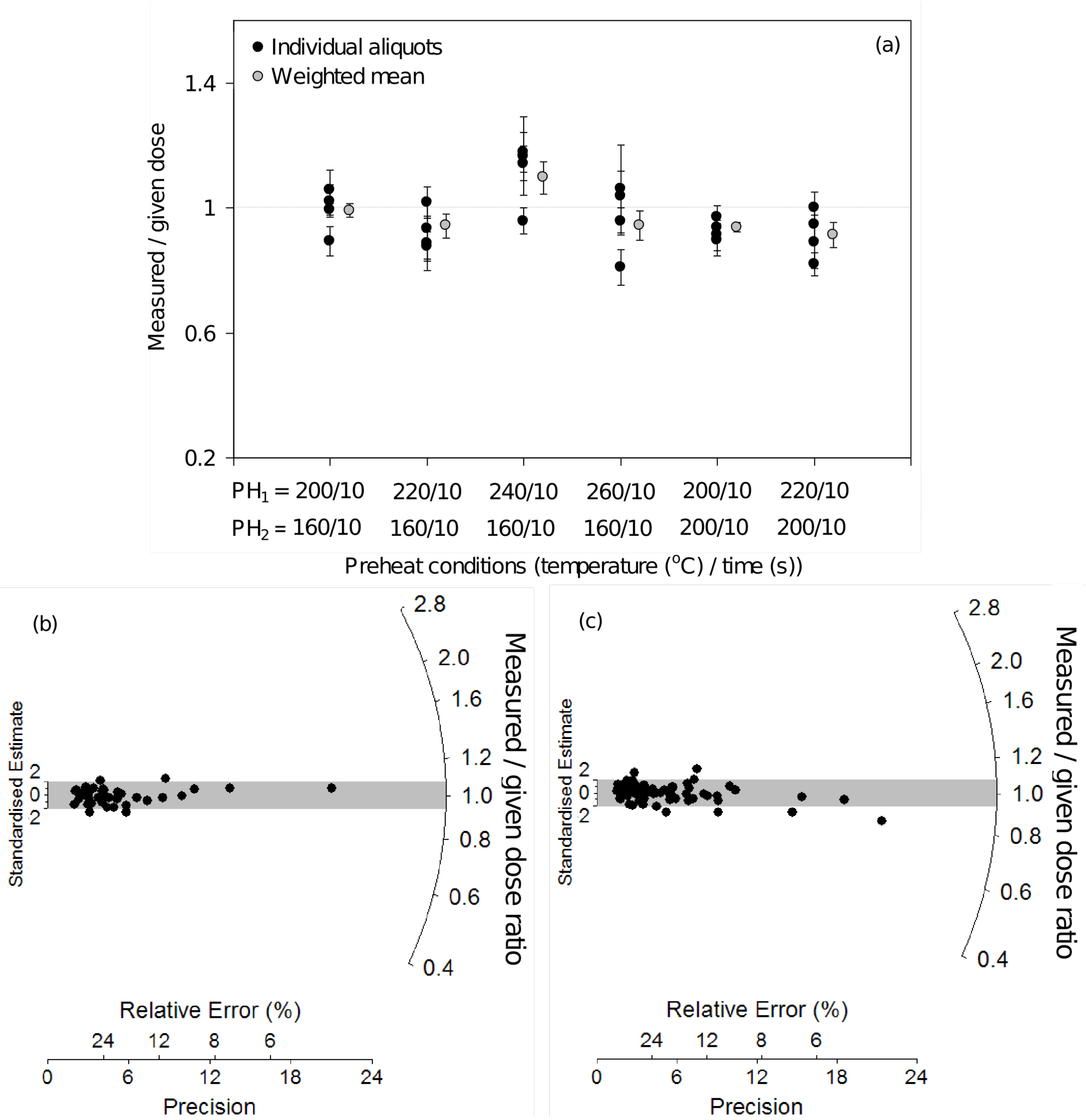

Fig. S36. Multiple-grain and single-grain OSL dose-recovery test results for samples from Aranbaltza II. (a) Measured-to-given dose ratios versus regenerative dose preheat (PH_1) and test dose preheat (PH_2) temperature (held for 10 s) for ~180-grain aliquots. The natural OSL signals of the multi-grain aliquots were optically bleached with two 1000 s blue LED illuminations at ambient temperature, each separated by a 10 000 s pause. A known dose of 51 Gy was then administered to each aliquot and a multi-grain aliquot version of the SAR procedure shown in Table S8a was subsequently used to estimate this dose (replacing 125°C green laser stimulations with 125°C blue LED stimulations for 60

s, and inserting a 50°C IR bleach for 40 s prior to each OSL measurement to remove any feldspar signal contamination). (b) Measured-to-given dose ratios obtained for individual quartz grains of sample AZ13-3 in the single-grain SAR dose-recovery test. The grey shaded region is centred on the administered dose for each grain (sample average = 50 Gy). Individual D_e values that fall within the shaded region are consistent with the administered dose at 2σ. (c) Measured-to-given dose ratios obtained for individual quartz grains of sample AAM16-10 in the single-grain SAR dose-recovery test. The grey shaded region is centred on the administered dose for each grain (sample average = 40 Gy). Individual D_e values that fall within the shaded region are consistent with the administered dose at 2σ.

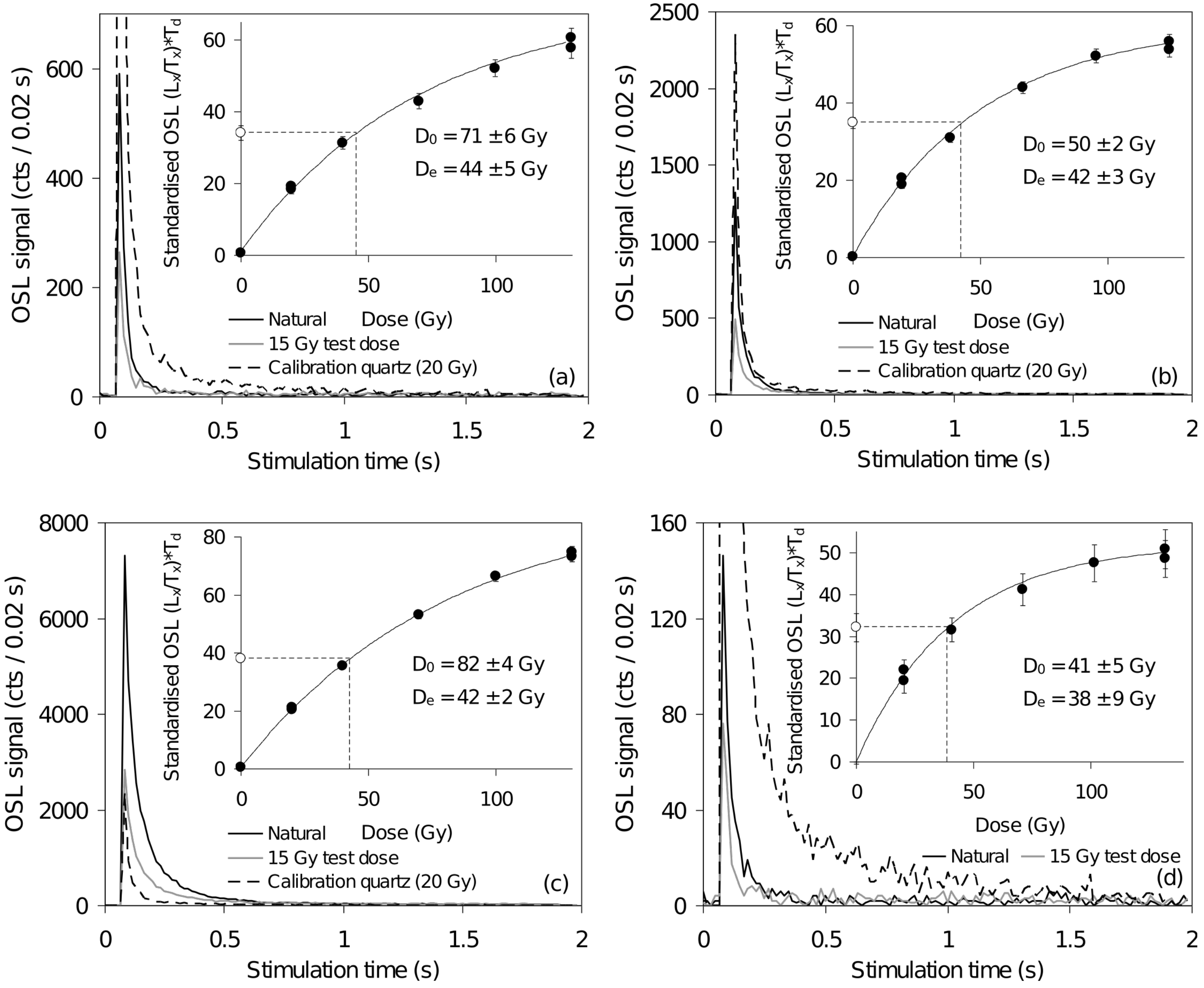

Fig. S37. Representative single-grain OSL decay and dose-response curves for quartz grains from sample AZ13-1. The decay curve of a fast-component dominated calibration quartz grain is shown for comparison (Risø calibration quartz standard from Rømø, batch #98; [139]). In the insets, the open circle denotes the sensitivity-corrected natural OSL signal, and filled circles denote the sensitivity-corrected regenerated OSL signals. The D_0 value characterises the rate of signal saturation with respect to administered dose and equates to the dose value for which the saturating exponential dose-response curve slope is 1/e (or ~ 0.37) of its initial value. (a) grain with moderate OSL signal brightness (T_n intensity = 200–1,000 counts / 0.08 s). (b) grain with bright OSL signal (T_n intensity = ~1,000–5,000 counts / 0.08 s). (c) grain with very bright OSL signal (T_n intensity = >5,000 counts / 0.08 s). (d) grain with relatively dim OSL signal (T_n intensity = <200 counts / 0.08 s).

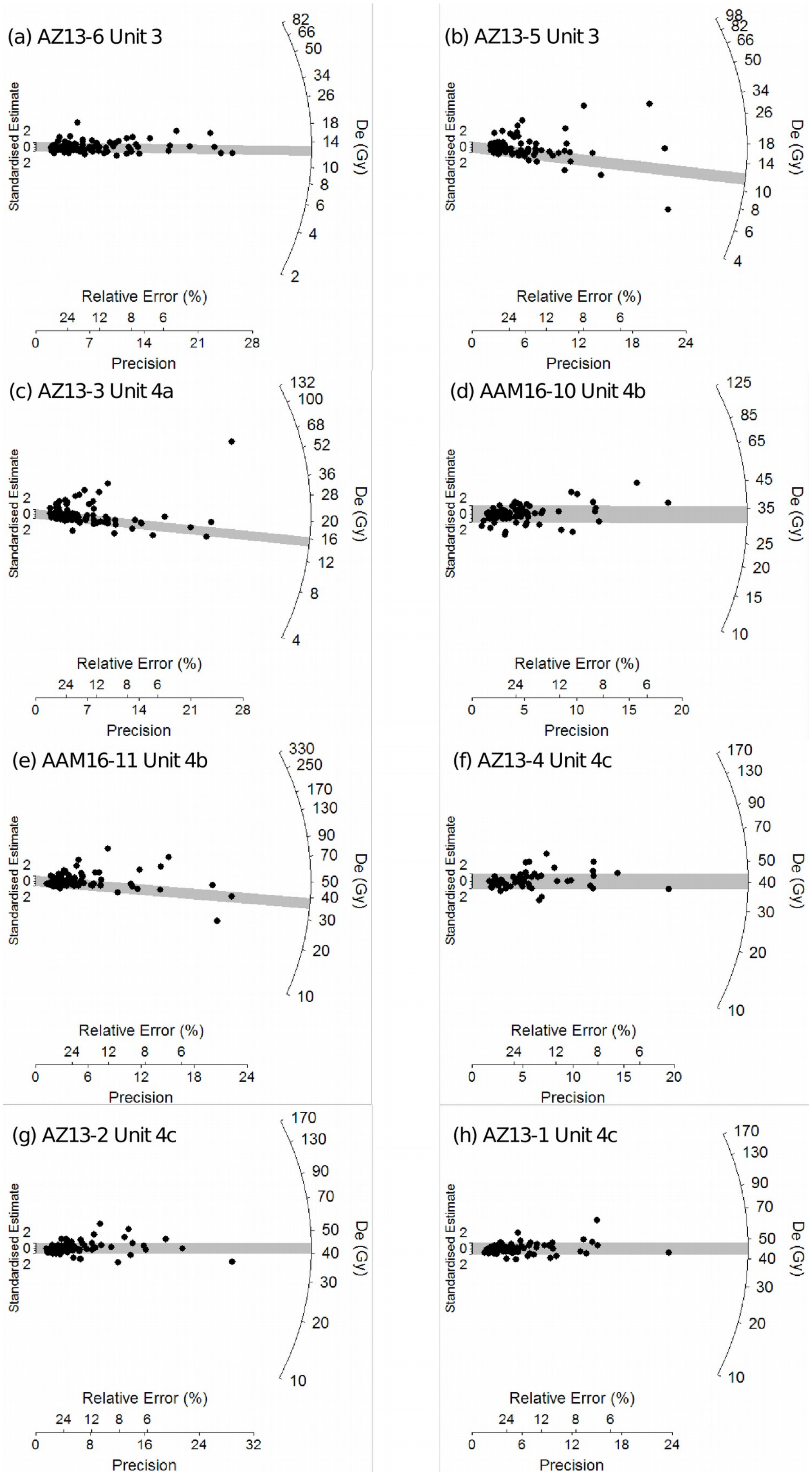
(a) AZ13-6 Unit 3
Standardised Estimate
2 0 2
De (Gy)
82 66 50 34 26 18 14 10 8 6 4 2
Relative Error (%)
24 12 8 6
0 7 14 21 28
Precision
(b) AZ13-5 Unit 3
Standardised Estimate
2 0 2
De (Gy)
98 82 66 50 34 26 18 14 10 8 6 4
Relative Error (%)
24 12 8 6
0 6 12 18 24
Precision
(c) AZ13-3 Unit 4a
Standardised Estimate
2 0 2
De (Gy)
132 100 68 52 36 28 20 16 12 8 4
Relative Error (%)
24 12 8 6
0 7 14 21 28
Precision
(d) AAM16-10 Unit 4b
Standardised Estimate
2 0 2
De (Gy)
125 85 65 45 35 25 20 15 10
Relative Error (%)
24 12 8 6
0 5 10 15 20
Precision
(e) AAM16-11 Unit 4b
Standardised Estimate
2 0 2
De (Gy)
330 250 170 130 90 70 50 40 30 20 10
Relative Error (%)
24 12 8 6
0 6 12 18 24
Precision
(f) AZ13-4 Unit 4c
Standardised Estimate
2 0 2
De (Gy)
170 130 90 70 50 40 30 20 10
Relative Error (%)
24 12 8 6
0 5 10 15 20
Precision
(g) AZ13-2 Unit 4c
Standardised Estimate
2 0 2
De (Gy)
170 130 90 70 50 40 30 20 10
Relative Error (%)
24 12 8 6
0 8 16 24 32
Precision
(h) AZ13-1 Unit 4c
Standardised Estimate
2 0 2
De (Gy)
170 130 90 70 50 40 30 20 10
Relative Error (%)
24 12 8 6
0 6 12 18 24
Precision

Fig. S38. Single-grain OSL D_e distributions for the eight OSL samples dated in this study, shown as radial plots. The grey bands are centred on the D_e values used for the age calculations, which were derived using either the central age model (samples AZ13-4, AZ13-5, AZ13-6, AAM16-10), 3-parameter minimum age model (samples AZ13-5, AZ13-6, AAM16-11) or the 4-parameter minimum age model (sample AZ13-3) of Galbraith et al. [140].

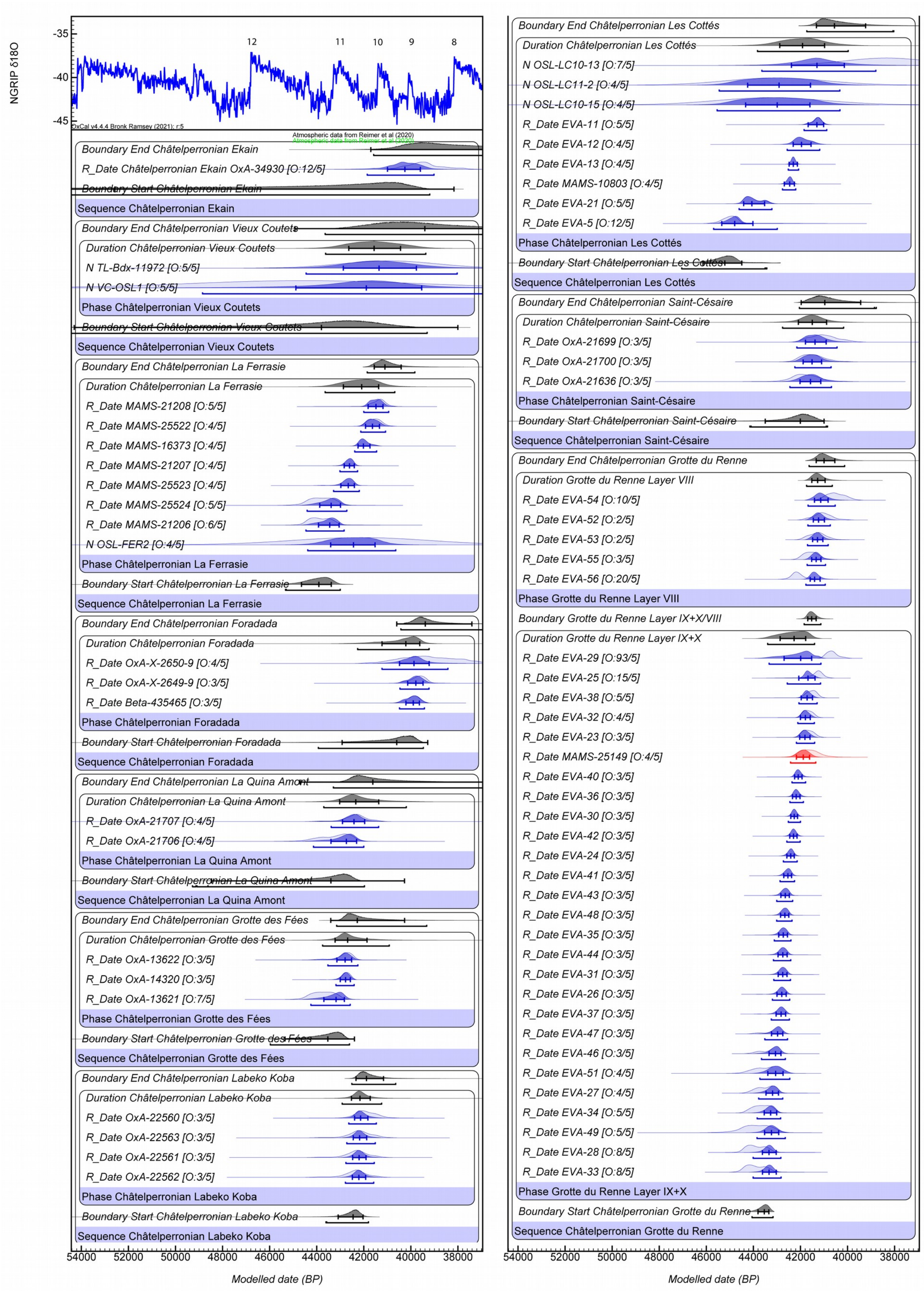

NGRIP δ18O
-35
-40
-45
12
11
10
9
8
OxCal v4.4.4 Bronk Ramsey (2021); r:5
Atmospheric data from Reimer et al (2020)
Boundary End Châtelperronian Ekain
R_Date Châtelperronian Ekain OxA-34930 [O:12/5]
Boundary Start Châtelperronian Ekain
Sequence Châtelperronian Ekain
Boundary End Châtelperronian Vieux Coutets
Duration Châtelperronian Vieux Coutets
N TL-Bdx-11972 [O:5/5]
N VC-OSL1 [O:5/5]
Phase Châtelperronian Vieux Coutets
Boundary Start Châtelperronian Vieux Coutets
Sequence Châtelperronian Vieux Coutets
Boundary End Châtelperronian La Ferrasie
Duration Châtelperronian La Ferrasie
R_Date MAMS-21208 [O:5/5]
R_Date MAMS-25522 [O:4/5]
R_Date MAMS-16373 [O:4/5]
R_Date MAMS-21207 [O:4/5]
R_Date MAMS-25523 [O:4/5]
R_Date MAMS-25524 [O:5/5]
R_Date MAMS-21206 [O:6/5]
N OSL-FER2 [O:4/5]
Phase Châtelperronian La Ferrasie
Boundary Start Châtelperronian La Ferrasie
Sequence Châtelperronian La Ferrasie
Boundary End Châtelperronian Foradada
Duration Châtelperronian Foradada
R_Date OxA-X-2650-9 [O:4/5]
R_Date OxA-X-2649-9 [O:3/5]
R_Date Beta-435465 [O:3/5]
Phase Châtelperronian Foradada
Boundary Start Châtelperronian Foradada
Sequence Châtelperronian Foradada
Boundary End Châtelperronian La Quina Amont
Duration Châtelperronian La Quina Amont
R_Date OxA-21707 [O:4/5]
R_Date OxA-21706 [O:4/5]
Phase Châtelperronian La Quina Amont
Boundary Start Châtelperronian La Quina Amont
Sequence Châtelperronian La Quina Amont
Boundary End Châtelperronian Grotte des Fées
Duration Châtelperronian Grotte des Fées
R_Date OxA-13622 [O:3/5]
R_Date OxA-14320 [O:3/5]
R_Date OxA-13621 [O:7/5]
Phase Châtelperronian Grotte des Fées
Boundary Start Châtelperronian Grotte des Fées
Sequence Châtelperronian Grotte des Fées
Boundary End Châtelperronian Labeko Koba
Duration Châtelperronian Labeko Koba
R_Date OxA-22560 [O:3/5]
R_Date OxA-22563 [O:3/5]
R_Date OxA-22561 [O:3/5]
R_Date OxA-22562 [O:3/5]
Phase Châtelperronian Labeko Koba
Boundary Start Châtelperronian Labeko Koba
Sequence Châtelperronian Labeko Koba
54000 52000 50000 48000 46000 44000 42000 40000 38000
Modelled date (BP)
Boundary End Châtelperronian Les Cottés
Duration Châtelperronian Les Cottés
N OSL-LC10-13 [O:7/5]
N OSL-LC11-2 [O:4/5]
N OSL-LC10-15 [O:4/5]
R_Date EVA-11 [O:5/5]
R_Date EVA-12 [O:4/5]
R_Date EVA-13 [O:4/5]
R_Date MAMS-10803 [O:4/5]
R_Date EVA-21 [O:5/5]
R_Date EVA-5 [O:12/5]
Phase Châtelperronian Les Cottés
Boundary Start Châtelperronian Les Cottés
Sequence Châtelperronian Les Cottés
Boundary End Châtelperronian Saint-Césaire
Duration Châtelperronian Saint-Césaire
R_Date OxA-21699 [O:3/5]
R_Date OxA-21700 [O:3/5]
R_Date OxA-21636 [O:3/5]
Phase Châtelperronian Saint-Césaire
Boundary Start Châtelperronian Saint-Césaire
Sequence Châtelperronian Saint-Césaire
Boundary End Châtelperronian Grotte du Renne
Duration Grotte du Renne Layer VIII
R_Date EVA-54 [O:10/5]
R_Date EVA-52 [O:2/5]
R_Date EVA-53 [O:2/5]
R_Date EVA-55 [O:3/5]
R_Date EVA-56 [O:20/5]
Phase Grotte du Renne Layer VIII
Boundary Grotte du Renne Layer IX+X/VIII
Duration Grotte du Renne Layer IX+X
R_Date EVA-29 [O:93/5]
R_Date EVA-25 [O:15/5]
R_Date EVA-38 [O:5/5]
R_Date EVA-32 [O:4/5]
R_Date EVA-23 [O:3/5]
R_Date MAMS-25149 [O:4/5]
R_Date EVA-40 [O:3/5]
R_Date EVA-36 [O:3/5]
R_Date EVA-30 [O:3/5]
R_Date EVA-42 [O:3/5]
R_Date EVA-24 [O:3/5]
R_Date EVA-41 [O:3/5]
R_Date EVA-43 [O:3/5]
R_Date EVA-48 [O:3/5]
R_Date EVA-35 [O:3/5]
R_Date EVA-44 [O:3/5]
R_Date EVA-31 [O:3/5]
R_Date EVA-26 [O:3/5]
R_Date EVA-37 [O:3/5]
R_Date EVA-47 [O:3/5]
R_Date EVA-46 [O:3/5]
R_Date EVA-51 [O:4/5]
R_Date EVA-27 [O:4/5]
R_Date EVA-34 [O:5/5]
R_Date EVA-49 [O:5/5]
R_Date EVA-28 [O:8/5]
R_Date EVA-33 [O:8/5]
Phase Grotte du Renne Layer IX+X
Boundary Start Châtelperronian Grotte du Renne
Sequence Châtelperronian Grotte du Renne
54000 52000 50000 48000 46000 44000 42000 40000 38000
Modelled date (BP)

Fig. S38. OxCal 4.4.3 [77] Bayesian modelling results used to constrain the timing of the Châtelperronian culture at individual sites from France and the Iberian Peninsula. Previously published ^{14}C, OSL and TL ages obtained on Châtelperronian layers at each site have been combined in a single *Phase* or *Sequence* model with delineating start and end boundaries. The radiocarbon ages included in the site-specific models have been calibrated against the IntCal20 calibration curve [98]. The unmodelled age distributions for the dating samples (likelihoods) are shown in light blue shading (light red shading for hominin remains), while the modelled posterior distributions for the dating samples are shown in dark blue shading (dark red shading for hominin remains). The modelled posterior distributions for the *Phase* and *Sequence* boundaries are shown in grey. The site-specific models include a general t type outlier function [78] with a prior outlier probability of 5% assigned to each likelihood. The modelled duration of the Châtelperronian culture at individual sites that contain multiple dating results (also shown in grey) have been calculated from the modelled posterior probabilities of the start and end boundaries using the *Date* command. The median ages and 1σ uncertainty ranges are shown for the modelled probability distributions. The 95.4% ranges of the highest posterior probabilities are also indicated by the broader horizontal bars underneath the probability density functions. The modelled durations are compared against the NGRIP GICC05 $\delta^{18}O$ record, with the interstadials (milder climatic periods) numbered accordingly.

Table S1. Mineralogy, granulometry and organic carbon content data for the Aranbaltza II sedimentary sequence from the AAR4 core.

Stratigraphic Unit	Sample	Quartz	Feldspars	Clay minerals	Fe Ox.	Al Ox.	C_{org}	Sand	Silt	Clay	HSC	Convexity	Aspect ratio
**Unit 1**	AAR-0	75,63	9,77	13,37	0,84	0,4	4,39	43,01	39,01	17,35	0,865	0,983	0,802
	AAR-10	81,88	6,4	10,32	0,81	0,58	6,13	48,72	35,35	14,94	0,862	0,982	0,794
**Unit 2**	AAR-20	87	5,83	6,53	0,21	0,43	1,79	48,54	34,47	16,99	0,861	0,983	0,793
	AAR-30	82,97	8,54	7,61	0,25	0,62	1,86	45,13	30,13	22,25	0,867	0,984	0,796
	AAR-40	78,97	10,19	8,97	0,67	1,2	1,92	33,75	39,68	21,57	0,864	0,983	0,794
	AAR-50	85,92	7	4,9	0,29	1,89	2,15	41,44	38,72	16,40	0,86	0,981	0,801
**Unit 3**	AAR-60	82,34	9,32	7,27	0	1,07	1,32	47,24	35,66	16,69	0,863	0,984	0,791
	AAR-70	83,14	6,93	7,78	0,77	1,38	1,78	48,93	35,39	15,68	0,867	0,985	0,786
	AAR-80	76,31	9,4	11,52	1,22	1,55	1,49	49,05	33,56	17,39	0,869	0,985	0,8
	AAR-90	80,83	12,64	5,44	0,76	0,32	1,41	48,49	35,75	15,76	0,868	0,984	0,805
	AAR-100	83,28	8,79	7,14	0,35	0,43	1,03	50,10	35,88	14,02	0,866	0,984	0,786
	AAR-110	79,78	8,59	10,18	0,92	0,55	1,09	52,19	32,68	15,13	0,869	0,984	0,795
	AAR-120	81,92	10,35	5,42	0,78	1,53	0,83	54,09	32,29	13,62	0,864	0,984	0,786
	AAR-130	80,23	8,08	10,44	0,83	0,4	1,10	50,32	32,68	17,01	0,865	0,984	0,791
**Unit 4a**	AAR-140	81,57	7,72	7,99	0,83	1,89	1,42	56,34	28,44	15,22	0,867	0,985	0,793
	AAR-150	85,15	7,16	5,91	0,75	1,02	1,24	55,86	30,78	13,36	0,867	0,984	0,796
**Unit 4b**	AAR-160	92,49	5,46	0,97	0,8	0,29	0,31	89,63	4,77	5,59	0,87	0,985	0,799
**Unit 4c**	AAR-170	88,87	6,76	2,62	0,54	1,21	0,35	83,89	7,70	8,41	0,874	0,985	0,807
	AAR-180	94,17	2,08	2,54	0,15	1,07		76,86	9,55	13,58	0,869	0,985	0,795
	AAR-190	88,03	5,63	3,42	0,99	1,94	1,35	75,13	12,89	11,99	0,869	0,984	0,81
**Unit 5**	AAR-200	88,93	5,04	4,45	0,7	0,87	2,05	68,27	20,09	11,64	0,863	0,983	0,798

Table S2. Technological composition of I. Libano and J.A. Libano collections

I. Libano+ J.A. Libano collections	Sandstone	Quartzite	Ochre	Non-local flint	Flysch flint	Total	%
Core		1		3	121	**125**	**4.45**
Core preform					26	**26**	**0.92**
Cortical flake		1			104	**105**	**3.74**
Partially cortical flake					358	**358**	**12.74**
Cortical blade					17	**17**	**0.60**
Partially cortical blade					128	**128**	**4.55**
Outrepassing flake				2	204	**206**	**7.33**
Overshot flake					21	**21**	**0.75**
Hinged flake				1	8	**9**	**0.32**
Two-sided crested blade					35	**35**	**1.25**
Onesided crested blade					107	**107**	**3.81**
Flank blade				1	118	**119**	**4.23**
Overshot blade					154	**154**	**5.48**
Hinged blade				1	84	**85**	**3.02**
Blade core platform rejuvenation flake					57	**57**	**2.03**
Blade core rejuvenation flake					70	**70**	**2.49**
Blade core bottom rejuvenation flake					1	**1**	**0.04**
Flake				4	182	**186**	**6.62**
Blade				9	647	**656**	**23.34**
Kombewa flake					8	**8**	**0.28**
Resharpening flake					36	**36**	**1.28**
Burin spall					14	**14**	**0.50**
Bifacial resharpening flake					2	**2**	**0.07**
Splint					32	**32**	**1.14**
Chunk					232	**232**	**8.25**
Thermal flake					4	**4**	**0.14**
Used pebble/block	5	4	1	1	7	**18**	**0.64**
**Total**	**5**	**6**	**1**	**22**	**2777**	**2811**	**100.00**

Table S3. Core types in I. Libano and J.A. Libano collections

**Core type**	**Quartzite**	**Non-local flint**	**Flysch flint**	**Total**
Unidirectional blade core		1	70	**71**
Bidirectional blade core			12	**12**
Bladelet core on flake		1	16	**17**
Flake core	1		10	**11**
Flake core on flake		1	10	**11**
Exhausted core			3	**3**
Core roughout			26	**26**

Table S4. Retouched tools in the I. Libano and J.A. Libano collections

**Type**	**I. Libano + J.A. Libano collections**	**%**
Endscrapers	107	19
Borers and becs	56	9.9
Burins	31	5.5
Multiple tools	5	0.9
Châtelperronian points	18	3.2
Atypical Châtelperronian points	11	2
Backed blades	124	22
Truncated blades	31	5.5
Retouched blades	18	3.2
Notched blades	27	4.8
Denticulates	23	4.1
Splintered pieces	32	5.7
Sidescrapers	17	3
Retouched bladelets	9	1.6
Others	55	9.8
**Total**	**564**	
**% of total retouched**	**20%**	

Table S5. Technological composition of US4b assemblage. F: Flysch flint; SWF: Southwestern France flint; T: Translucent flint; Non-ID: Unidentified flint; S: Sandstone; Q: Quartzite; Qz: Quartz; SM: Siliceous Mudstone; O: Ochre; OP: Ophite; TR: Trachyte.

**US4b**	**Flint**				**S**	**Q**	**QZ**	**SM**	**M**	**O**	**OP**	**TR**	**Total**
	F	***SWF***	***T***	***Non-ID***									
Exhausted cores	7												**7**
Flake cores	4				2								**6**
Blade cores	7												**7**
Bladelet cores	5												**5**
Bladelet cores on flakes	5												**5**
Blade core roughouts	4												**4**
Flake core roughouts	2												**2**
Tested blocks	3												**3**
Cortical flakes	52			1		1							**54**
Partially cortical flakes	366	4		6	2	1							**379**
Cortical blades	14	1											**15**
Partially cortical blades	103	1											**104**
Outrepassing flakes	37				2								**39**
Overshot flakes	5				1								**6**
Hinged flakes	52	3		1									**56**
Bifacial crests	2												**2**
Unifacial crests	49			1									**50**
Flank blades	93	1		1									**95**
Overshot blades	21												**21**
Hinged blades	58	2											**60**
Platform rejuvenation flakes	28												**28**
Flaking surface rejuvenation flakes	33	1											**34**
Core bottom correction flakes	1												**1**
Flakes	323	5		6	9	2	1	1				4	**351**
Kombewa flakes	10												**10**
Blades	214			5									**219**
Bladelets	361	6		6									**373**
Resharpening flakes	108			3									**111**
Burin spalls	36			1									**37**

Splints	147	6		1								1	**155**
Chunks	284			3	5	1	1	1				1	**296**
Chips	2683	6	1	23	9	8	16				1	1	**2748**
Thermal flakes	52			1	2								**55**
Retouched natural fragments	2												**2**
Used pebbles/blocks	4				4				1				**9**
Natural blocks/fragments	203				83	5	3			30		13	**337**
**Total**	**5378**	**36**	**1**	**59**	**119**	**18**	**21**	**2**	**1**	**30**	**1**	**20**	5686
**%**	**94.58**	**0.63**	**0.02**	**1.04**	**2.09**	**0.32**	**0.37**	**0.04**	**0.02**	**0.53**	**0.02**	**0.35**	**100**

Table S6. Different blade product group composition

	%	Mean width (mm)	Stdev
Bladelets	20.3	5.0646	1.2356
Narrow blades	51.9	9.5013	2.6059
Wide blades	27.8	15.265	4.3081

Table S7: Typological composition of US4b assemblage.

US4b retouched tool list	Total
Endscraper on blade	**1**
Endscraper on flake	**2**
Borer	**2**
Atypical borer	**3**
Angle dihedral burin	**3**
Burin on retouched truncation	**2**
Burin on fracture	**1**
Châtelperronian point	**2**
Châtelperronian point (fragment)	**8**
Backed blade	**1**
Backed blade (fragment)	**4**
Partially backed blade (fragment)	**2**
Marginally backed point	**1**
Marginally backed point (distal fragment)	**2**
Marginally backed blade	**3**
Marginally backed blade (fragment)	**12**
Partial marginally backed blade	**1**
Partial marginally backed blade (fragment)	**2**
Blade with marginal back on ventral surface	**2**
Blade with marginal back on ventral surface (fragment)	**4**
Piece with straight retouched truncation	**2**
Piece with oblique retouched truncation	**3**
Blade with continuous retouch on one edge	**4**
Partially retouched blade	**4**
Notched piece	**11**
Denticulated piece	**4**
Splintered piece	**1**
Sidescraper	**3**
Marginally backed flake	**3**
Partially retouched flake	**10**
Truncated bladelet	**1**
Backed bladelet	**5**
Dufour bladelet	**1**
Marginally backed bladelet	**4**
Partial marginally backed bladelet	**1**
Partially retouched bladelet	**2**
**Total**	**117**

Table S8. AMS ^{14}C results obtained from charred vegetation materials at Aranbaltza II. ^{14}C ages have been calibrated with OxCal 4.4. [77] and the IntCal20 curve [89].

**Sample Number**	**Reference**	**Unit**	**Method**	**Material**	**Taxon**	**Uncalibrated age (^{14}C yr BP)**	**δ 13 C**	**Cal BP (95%)**
Beta-401309	AAM.14.20104	US2	(AAA)	Charcoal		4170±30	-25.7	4832-4581
Beta-401310	AAM.14.20118	US2	(AAA)	Charcoal		3690±30	-25.6	4146-3924
Poz-82701	AAM.2015.29009	US3b	(AAA)	Charcoal	*Quercus* sp.	4920±40		5730-5587
Beta-370964	AAR.13.1355	US3	(Acid)	Organic Material		8090±40	-27.4	9251-8779
Beta-365219	AAR.13.1195	US3	(Acid)	Charcoal	*Quercus* sp.	sample dissolved with acid treatment		
Beta-370966	AAR.13.2100	US3	(AAA)	Charcoal		7990 ±40	-24.6	9000-8650
Beta-370965	AAR.13.2003	US3	(AAA)	Charcoal		8050 ±40	-23	9085-8728
Beta-365220	AAR.13.2291	US3	(AAA)	Charcoal		sample dissolved with acid treatment		
OxA-34891	AAM.16.401031	US4b	(AAA)	Charcoal	*Quercus* sp.	7890 ±50	-26.44	8984-8555
OxA-34987	AAM.16.401387	US4b	(AAA)	Charcoal	*Quercus* sp.	7220 ± 50	-23.21	8170-7944
P-42099	AAM.16.401303	US4b	(AAA)	Charcoal	*Quercus* sp.	Failed due to a very low carbon content		
P-42102	AAM.16.400444	US4b	(AAA)	Charcoal	*Quercus* sp.	Failed due to no yield		
Beta-365221	AAR.13.4053a	US4b	(AAA)	Charcoal	Angiosperm	sample dissolved with acid treatment		

Table S9. Single-aliquot regenerative-dose (SAR) procedures used for dose-recovery measurements and D_e determination. Each of these SAR measurement cycles was repeated for the natural dose, 5 different sized regenerative doses and a 0 Gy regenerative-dose (to measure OSL signal recuperation). Both the smallest and largest non-zero regenerative-dose cycles were repeated at the end of the SAR procedure to assess the suitability of the test-dose sensitivity correction. The smallest regenerative-dose cycle was also repeated a second time with the inclusion of step 2 to check for the presence of feldspar contaminants using the OSL IR depletion ratio of Duller [96]. L_x = regenerative dose signal response; L_n = natural dose signal response; T_x = test dose signal response for a laboratory dose cycle T_n = test dose signal response for the natural dose cycle.

**Table S9a: Single-grain OSL SAR procedure 2013 samples**			**Table S9b: Single-grain OSL SAR procedure 2016 samples**	
**Step**	**Treatment**	**Symbol**	**Step**	**Treatment**
1	Dose (Natural or laboratory)	N or D	1	Dose (Natural or laboratory)
2 [a]	IRSL stimulation (50ºC for 60 s)		2 [a]	IRSL stimulation (50ºC for 60 s)
3	Preheat 1 (200ºC for 10 s)	PH_1	3	Preheat 1 (220ºC for 10 s)
4	Single-grain OSL stimulation (125ºC for 2 s)	L_n or L_x	4	Single-grain OSL stimulation (125ºC for 2 s)
5	Test dose (15 Gy)	T_d	5	Test dose (10 Gy)
6	Preheat 2 (160ºC for 10 s)	PH_2	6	Preheat 2 (200 for 10 s)
7	Single-grain OSL stimulation (125ºC for 2 s)	T_n or T_x	7	Single-grain OSL stimulation (125ºC for 2 s)
8	Repeat measurement cycle for different		8	OSL stimulation (260 ºC for 40 s)
	sized regenerative doses		9	Repeat measurement cycle for different
				sized regenerative doses

[a] Step 2 is only included in the single-grain SAR procedure when measuring the OSL IR depletion ratio [106].

Table S10. Single-grain OSL classification statistics for the dose recovery and natural D_e measurements. The proportion of grains that were rejected from final D_e estimation after applying the various SAR quality assurance criteria are shown in rows 5-13. These criteria were applied to each single-grain measurement in the order listed. T_n = natural test dose signal response; L_n/T_n = sensitivity-corrected natural signal response; L_x/T_x = sensitivity-corrected regenerative-dose signal response; I_{max} = saturation OSL intensity of the fitted dose response curve.

**Sample name**	**AZ13-6**	**AZ13-5**	**AZ13-3**	**AZ13-3**	**AAM16-10**	**AAM16-10**	**AAM16-11**	**AZ13-4**	**AZ13-2**	**AZ13-1**
**SAR measurement type**	D_e	D_e	D_e	Dose recovery	D_e	Dose recovery	D_e	D_e	D_e	D_e
**Total measured grains (*n*)**	3000	3000	3000	1200	3400	2400	3000	2800	3000	3000
**Grains rejected for failing SAR quality assurance criteria (%)**										
T_n <3σ background	84	83	85	81	77	76	70	87	88	86
Low-dose recycling ratio ≠ 1 at ±2σ	2	3	3	4	3	3	3	3	2	3
High-dose recycling ratio ≠ 1 at ±2σ	1	2	1	2	2	2	4	1	1	1
OSL-IR depletion ratio <1 at ±2σ	2	2	2	3	3	4	5	2	2	2
0 Gy L_x/T_x >5% L_n/T_n	<1	<1	<1	<1	<1	<1	0	<1	<1	<1
Non-intersecting grains (L_n/T_n > dose response curve saturation)	<1	<1	<1	0	<1	0	<1	<1	<1	<1
Saturated grains (L_n/T_n ≥ dose response curve I_{max} at ±2σ)	0	<1	<1	0	<1	0	<1	<1	<1	<1
Extrapolated grains (L_n/T_n values >2σ beyond largest L_x/T_x value)	0	0	0	0	0	0	0	0	0	0
Anomalous dose response / unable to perform Monte Carlo fit	7	7	6	6	12	11	15	5	3	5
**Sum of rejected grains (%)**	97	97	97	96	97	97	97	98	97	97
**Sum of accepted grains (%)**	3	3	3	4	3	3	3	2	3	3

Table S11. Dose rate data, single-grain equivalent doses and quartz OSL ages for the Aranbaltza II samples.

Sample name	Unit	Grain size (µm)	Water content [a]	Environmental dose rate (Gy/ka)				Equivalent dose (D_e) data				OSL age (ka) [f,l]
				Beta dose rate [b,c]	Gamma dose rate [c,d]	Cosmic dose rate [e]	Total dose rate [c,f,g]	No. of grains [h]	Over-dispersion (%) [i]	Age model [j,k]	D_e (Gy) [f]	
AZ13-6	3	212 – 250	12 ± 2	0.64 ± 0.04	0.58 ± 0.02	0.17 ± 0.02	1.42 ± 0.07	102 / 3000	36 ± 3	MAM-3	12.4 ± 0.4	8.7 ± 0.5
AZ13-5	3	212 – 250	12 ± 2	0.66 ± 0.04	0.58 ± 0.02	0.16 ± 0.02	1.43 ± 0.07	83 / 3000	59 ± 5	MAM-3	11.6 ± 1.0	8.1 ± 0.8
AZ13-3	4a	212 – 250	15 ± 3	0.63 ± 0.04	0.49 ± 0.02	0.15 ±0.02	1.31 ± 0.07	89 / 3000	70 ± 6	MAM-4	15.5 ± 0.5	11.9 ± 0.8
AAM16-10	4b	212 – 250	11 ± 2	0.25 ± 0.01	0.30 ± 0.01	0.17 ± 0.02	0.75 ± 0.03	90 / 3400	32 ± 4	CAM	32.6 ± 1.5	43.5 ± 2.9
AAM16-11	4b	212 – 250	10 ± 2	0.28 ± 0.02	0.24 ± 0.01	0.17 ± 0.02	0.72 ± 0.03	81 / 3000	54 ± 5	MAM-3	37.2 ± 2.2	51.9 ± 4.0
AZ13-4	4c	212 – 250	11 ± 2	0.24 ± 0.01	0.28 ± 0.01	0.16 ± 0.02	0.71 ± 0.04	57 / 2800	35 ± 5	CAM	41.3 ± 2.3	58.2 ± 4.6
AZ13-2	4c	212 – 250	20 ± 4	0.19 ± 0.01	0.29 ± 0.01	0.14 ± 0.01	0.64 ± 0.04	94 / 3000	31 ± 3	CAM	42.0 ± 1.7	65.4 ± 4.9
AZ13-1	4c	212 – 250	18 ± 4	0.21 ± 0.01	0.20 ± 0.01	0.14 ± 0.01	0.58 ± 0.04	81 / 3000	25 ± 3	CAM	45.1 ± 1.7	78.1 ± 5.8

[a] Long-term water content, expressed as % of dry mass of mineral fraction, with an assigned relative uncertainty of ±20%. Long-term water contents are calculated as being equivalent to the present-day water contents for all samples except AAM16-10. The latter yielded an uncharacteristically low present-day water content of <6%; hence the long-term water content of this sample is based on the average measured water content of stratigraphically related samples AAM16-11 and AZ13-4 (see Supplementary Information for further details).
[b] Beta dose rates were calculated on dried and powdered sediment samples using a Risø GM-25-5 low-level beta counter [108] after making allowance for beta dose attenuation due to grain-size effects and HF etching [110]
[c] Specific activities and radionuclide concentrations have been converted to dose rates using the conversion factors given in Guérin *et al.* [118], making allowance for beta-dose attenuation [110, 119].
[d] Gamma dose rates were calculated from *in situ* measurements made at each sample position with a NaI:Tl detector, using the 'energy windows' approach (e.g., [101]).
[e] Cosmic-ray dose rates were calculated using the approach of Prescott and Hutton [141] and assigned a relative uncertainty of ±10%.
[f] Mean ± total uncertainty (68% confidence interval), calculated as the quadratic sum of the random and systematic uncertainties.
[g] Includes an internal dose rate of 0.03 Gy/ka with an assigned relative uncertainty of ±30%, based on intrinsic ^{238}U and ^{232}Th contents published by Mejdahl [112], Bowler et al. [113] Jacobs et al. [114 and Pawley et al. [115], and an a-value of 0.04 ± 0.01 [116-117].
[h] Number of D_e measurements that passed the SAR rejection criteria and were used for D_e determination / total number of grains analysed.
[i] The relative spread in the D_e dataset beyond that associated with the measurement uncertainties of individual D_e values, calculated using the central age model (CAM) of Galbraith et al. [129].
[j] Age model used to calculate the sample-averaged D_e value for each sample. CAM = central age model; MAM-3 = 3-parameter minimum age model; MAM-4 = 4-parameter minimum age model [129]. MAM-3 and MAM-4 D_e estimates were calculated after adding, in quadrature, a relative error of 20% to each individual D_e measurement error to approximate the underlying dose overdispersion observed in 'ideal' (well-bleached and unmixed) sedimentary samples from this site (i.e., sample AZ13-4), the single-grain dose-recovery tests performed on the Aranbaltza II samples (AZ13-3 and AAM16-10) and from global overdispersion datasets [125].
[k] Age model selection: The choice of whether to use the CAM, MAM-3 or MAM-4 for each sample has been made on statistical grounds using the maximum log likelihood score (L_{max}) criterion outlined by Arnold et al. [128].
[l] Total uncertainty includes a systematic component of ±2% associated with laboratory beta-source calibration.

Table S12. Single-grain OSL D_e summary statistics and age model results for the Aranbaltza II samples.

Sample	Total dose rate (Gy / ka)	No of D_e values [a]	Over-dispersion (%) [b]	Weighted skewness value [c]	Critical skewness 95% C.I. [c]	Age Model [d,e,f]	L_{max} score [g]	D_e (Gy) [h]	OSL age (ka) [h,I,j]
AZ13-6	1.42 ± 0.07	102 / 3000	36 ± 3	+1.81	±0.49	CAM	-58.99	13.1 ± 0.5	9.2 ± 0.6
						MAM-3	-45.36	12.4 ± 0.4	**8.7 ± 0.5**
						MAM-4	-45.33	12.4 ± 0.4	8.8 ± 0.6
AZ13-5	1.43 ± 0.07	83 / 3000	59 ± 5	+0.81	±0.54	CAM	-81.11	17.3 ± 1.2	12.1 ± 1.1
						MAM-3	-73.82	11.6 ± 1.0	**8.1 ± 0.8**
						MAM-4	-73.82	11.7 ± 1.9	8.2 ± 1.4
AZ13-3	1.31 ± 0.07	89 / 3000	70 ± 6	+0.73	±0.52	CAM	-99.54	22.3 ± 1.7	17.0 ± 1.7
						MAM-3	-78.68	15.0 ± 0.7	11.4 ± 0.9
						MAM-4	-72.71	15.5 ± 0.5	**11.9 ± 0.8**
						FMM	-72.71	15.8 ± 0.6	12.1± 0.9
AAM16-10	0.75 ± 0.03	90 / 3400	32 ± 4	-1.19	±0.52	CAM	-60.76	32.6 ± 1.5	**43.5 ± 2.9**
						MAM-3	-62.64	25.4 ± 2.7	33.9 ± 4.0
						MAM-4	-	-	-
AAM16-11	0.72 ± 0.03	81 / 3000	54 ± 5	+0.80	±0.54	CAM	-76.34	50.5 ± 3.4	70.5 ± 5.9
						MAM-3	-66.61	37.2 ± 2.2	**51.9 ± 4.0**
						MAM-4	-66.31	37.7 ± 2.1	52.7 ± 3.9
						FMM	-68.83	43.9 ± 2.6	61.4 ± 4.8
AZ13-4	0.71 ± 0.04	57 / 2800	35 ± 5	+0.21	±0.65	CAM	-32.05	41.3 ± 2.3	**58.2 ± 4.6**
						MAM-3	-31.52	36.3 ± 4.2	51.2 ± 6.5
						MAM-4	-31.50	35.3 ± 2.8	49.7 ± 4.9
AZ13-2	0.64 ± 0.04	94 / 3000	31 ± 3	+0.28	±0.51	CAM	-46.51	42.0 ± 1.7	**65.4 ± 4.9**
						MAM-3	-45.10	37.5 ± 4.1	58.3 ± 7.3
						MAM-4	-45.23	32.9 ± 4.9	51.2 ± 8.3
AZ13-1	0.58 ± 0.04	81 / 3000	25 ± 3	+0.04	±0.54	CAM	-30.06	45.1 ± 1.7	**78.1 ± 5.8**
						MAM-3	-28.48	43.4 ± 1.5	75.3 ± 5.5
						MAM-4	-28.46	43.3 ± 6.7	75.1 ± 12.5

[a] Number of D_e measurements that passed the SAR rejection criteria / total number of grains analysed.
[b] The relative spread in the D_e dataset beyond that associated with the measurement uncertainties of individual D_e values, calculated using the central age model [129].
[c] Weighted skewness scores have been calculated on log-transformed D_e values using Eq. 7-8 of Arnold and Roberts [125]. Critical skewness scores have been calculated using Eq. 16 of Bailey and Arnold [126]. Critical skewness values are taken to be equivalent to twice the standard error of skewness score (95% C.I.) for single-grain D_e datasets, following the results of sensitivity analyses performed by Bailey and Arnold [126] and Arnold et al. [135].

[d] CAM = central age model; MAM-3 = 3-parameter minimum age model; MAM-4 = 4-parameter minimum age model [129]; FMM = finite mixture model [140]. Note the MAM-4 could not be successfully fitted to sample AAM16-10 due to parameter redundancy.
[e] MAM-3 and MAM-4 D_e estimates were calculated after adding, in quadrature, a relative error of 20% to each individual D_e measurement error to approximate the underlying dose overdispersion observed in 'ideal' (well-bleached and unmixed) sedimentary samples from this site (i.e., sample AZ13-4), the single-grain dose-recovery tests performed on the Aranbaltza II samples (AZ13-3 and AAM16-10) and from global overdispersion datasets [125].
[f] The FMM was fitted by varying the common overdispersion parameter (σk) between 20 and 40% and incrementally increasing the specified number of k_n components. The FMM parameter values shown here were obtained from the optimum FMM fit (i.e., the fit with the lowest BIC score; [125]). Using this approach, the D_e distributions of both AZ13-3 and AAM16-11 are shown to contain two discrete dose populations (K_1 and K_2). The FMM D_e values shown in this table have been calculated using the dominant FMM dose component (i.e., that containing the majority of individual grains), which equates to the K_1 component for both samples AZ13-3 and AAM16-11.
[g] Maximum log likelihood score of the CAM, MAM-3, MAM-4 and FMM fit. For a given sample, the L_{max} score of the MAM-3 is expected to be substantially higher (i.e. at least 1.92 greater) than that of the CAM when the addition of the extra model parameter improves the fit to the data. Likewise, the L_{max} score of the MAM-4 is expected to be significantly greater than that of the MAM-3 (by at least 1.92 when compared with the 95% C.I. of a X^2 distribution) when the addition of the extra model parameter improves the fit to the data. If the extra parameter of the MAM-3 (or MAM-4) is not supported by the data, then its L_{max} score will be similar to (i.e. within 1.92 of) the CAM (or MAM-3) L_{max} score, indicating that the simpler age model explains the data equally well [128].
[h] Total uncertainty includes a systematic component of ±2% associated with laboratory beta-source calibration.
[i] The preferred final age is shown in bold. For these samples, the final age has been derived using the statistical age model (CAM, MAM-3 or MAM-4) that yielded the optimum L_{max} score, following the criterion outlined in footnote [g] and Arnold et al. [128]. The FMM ages of samples AZ13-3 and AAM16-11 are shown for comparative purposes only.
[j] Mean ± total uncertainty (68% confidence interval), calculated as the quadratic sum of the random and systematic uncertainties. Total uncertainty includes a systematic component of ± 2% associated with laboratory beta-source calibration.

Table S13. Available reliable ages for the Châtelperronian in France and the Iberian Peninsula. This table includes ^{14}C ages obtained from non-human bones using ultrafiltration (UF) pretreatment, charcoal ^{14}C ages obtained for Cova Foradada using acid-base-oxidation stepped combustion (ABOx-SC) and standard acid-base-acid (ABA) pretreatment, as well as the available thermoluminescence (TL) and optically stimulated luminescence (OSL) ages for Châtelperronian sites in this region.

Site	Level	Lab reference	Method	Age (^{14}C yr B.P. or yr B.P. for OSL/TL)	±1σ Err	Reference
Grotte du Renne (Arcy-sur-Cure)	VIII	OxA-X-2279-14[a]	UF	35450	750	[13]
Grotte du Renne (Arcy-sur-Cure)	VIII	EVA-54	UF	35380	390	[14]
Grotte du Renne (Arcy-sur-Cure)	VIII	EVA-52	UF	35980	432	[14]
Grotte du Renne (Arcy-sur-Cure)	VIII	EVA-53	UF	36230	435	[14]
Grotte du Renne (Arcy-sur-Cure)	VIII	EVA-55	UF	36630	452	[14]
Grotte du Renne (Arcy-sur-Cure)	VIII	OxA-21573[a]	UF	36800	1000	[13]
Grotte du Renne (Arcy-sur-Cure)	VIII	EVA-56	UF	37710	533	[14]
Grotte du Renne (Arcy-sur-Cure)	VIII	OxA-21683[a]	UF	40000	1200	[13]
Grotte du Renne (Arcy-sur-Cure)	Xb2	EVA-29	UF	35500	216	[14]
Grotte du Renne (Arcy-sur-Cure)	Xb1	EVA-25	UF	36210	250	[14]
Grotte du Renne (Arcy-sur-Cure)	IXa	EVA-38	UF	36540	248	[14]
Grotte du Renne (Arcy-sur-Cure)	Xb2	EVA-32	UF	36820	257	[14]
Grotte du Renne (Arcy-sur-Cure)	Xb1	EVA-23	UF	36840	335	[14]
Grotte du Renne (Arcy-sur-Cure)	X	EVA-40	UF	37510	275	[14]
Grotte du Renne (Arcy-sur-Cure)	IXb	EVA-36	UF	37740	307	[14]
Grotte du Renne (Arcy-sur-Cure)	Xb2	EVA-30	UF	37980	284	[14]
Grotte du Renne (Arcy-sur-Cure)	Xa	EVA-42	UF	38070	311	[14]
Grotte du Renne (Arcy-sur-Cure)	Xb1	EVA-24	UF	38400	317	[14]
Grotte du Renne (Arcy-sur-Cure)	Xa	EVA-41	UF	38730	333	[14]
Grotte du Renne (Arcy-sur-Cure)	Xa	EVA-43	UF	39020	352	[14]
Grotte du Renne (Arcy-sur-Cure)	Xb2	EVA-48	UF	39070	332	[14]
Grotte du Renne (Arcy-sur-Cure)	IXb	EVA-35	UF	39240	341	[14]
Grotte du Renne (Arcy-sur-Cure)	IXa	EVA-44	UF	39280	351	[14]
Grotte du Renne (Arcy-sur-Cure)	Xb2	EVA-31	UF	39290	334	[14]
Grotte du Renne (Arcy-sur-Cure)	Xb1	EVA-26	UF	39390	334	[14]
Grotte du Renne (Arcy-sur-Cure)	IXb	EVA-37	UF	39450	340	[14]
Grotte du Renne (Arcy-sur-Cure)	IXa	EVA-47	UF	39750	360	[14]
Grotte du Renne (Arcy-sur-Cure)	IXa	EVA-46	UF	39930	361	[14]
Grotte du Renne (Arcy-sur-Cure)	Xb2	EVA-51	UF	39960	702	[14]
Grotte du Renne (Arcy-sur-Cure)	Xb1	EVA-27	UF	40230	395	[14]
Grotte du Renne (Arcy-sur-Cure)	IXb	EVA-34	UF	40520	389	[14]
Grotte du Renne (Arcy-sur-Cure)	Xb2	EVA-49	UF	40830	778	[14]
Grotte du Renne (Arcy-sur-Cure)	Xb1	EVA-28	UF	40930	393	[14]
Grotte du Renne (Arcy-sur-Cure)	IXb	EVA-33	UF	40970	424	[14]
Grotte du Renne (Arcy-sur-Cure)	IX	OxA-21574[a]	UF	38800	1300	[13]
Grotte du Renne (Arcy-sur-Cure)	IX	OxA-21575[a]	UF	32100	550	[13]

Grotte du Renne (Arcy-sur-Cure)	X	OxA-21565[a]	UF	37900	900	[13]
Grotte du Renne (Arcy-sur-Cure)	X	OxA-21557[a]	UF	38100	1300	[13]
Grotte du Renne (Arcy-sur-Cure)	X	OxA-21576[a]	UF	40800	1700	[13]
Grotte du Renne (Arcy-sur-Cure)	X	OxA-X-2222-21[a]	UF	23120	190	[13]
Grotte du Renne (Arcy-sur-Cure)	X	OxA-21577[a]	UF	34650	800	[13]
Grotte du Renne (Arcy-sur-Cure)	X	OxA-X-2226-7[a]	UF	38500	1300	[13]
Grotte du Renne (Arcy-sur-Cure)	X	OxA-21590[a]	UF	21150	160	[13]
Grotte du Renne (Arcy-sur-Cure)	X	OxA-21591[a]	UF	34750	750	[13]
Grotte du Renne (Arcy-sur-Cure)	X	OxA-21592[a]	UF	36200	1100	[13]
Grotte du Renne (Arcy-sur-Cure)	X	OxA-21593[a]	UF	35300	900	[13]
Grotte du Renne (Arcy-sur-Cure)	X	OxA-X-2226-12[a]	UF	41500	1900	[13]
Grotte du Renne (Arcy-sur-Cure)	X	OxA-X-2226-13[a]	UF	39000	1400	[13]
Grotte du Renne (Arcy-sur-Cure)	X	OxA-X-2279-18[a]	UF	40600	1300	[13]
Grotte du Renne (Arcy-sur-Cure)	X	OxA-X-2279-44[a]	UF	48700	3600	[13]
Grotte du Renne (Arcy-sur-Cure)	X	OxA-X-2279-45[a]	UF	40900	1300	[13]
Grotte du Renne (Arcy-sur-Cure)	X	OxA-X-2279-46[a]	UF	38700	1000	[13]
Saint-Césaire	Ejop Sup	OxA-21699	UF	36000	700	[137]
Saint-Césaire	Ejop Sup	OxA-21700	UF	36650	750	[137]
Saint-Césaire	Ejop Sup	OxA-21636	UF	37200	1000	[137]
Les Cottés	6	EVA-11	UF	36230	210	[142]
Les Cottés	6	EVA-12	UF	37360	610	[142]
Les Cottés	6	EVA-13	UF	38100	210	[142]
Les Cottés	6	MAMS-10803	UF	38540	270	[142]
Les Cottés	6	EVA-21	UF	41070	300	[142]
Les Cottés	6	EVA-5	UF	42360	370	[142]
Labeko Koba	IX Lower	OxA-22560	UF	37400	800	[50]
Labeko Koba	IX Lower	OxA-22563	UF	37800	900	[50]
Labeko Koba	IX Lower	OxA-22561	UF	38000	900	[50]
Labeko Koba	IX Lower	OxA-22562	UF	38100	900	[50]
Ekain	X	OxA-34930	UF	34350	550	[16]
Grotte des Fées at Châtelperron	B5	OxA-13622	UF	39150	600	[143]
Grotte des Fées at Châtelperron	B5	OxA-14320	UF	39240	380	[143]
Grotte des Fées at Châtelperron	B5	OxA-13621	UF	40650	600	[143]
La Quina Amont	B2	OxA-21707	UF	38100	900	[137]
La Quina Amont	B3	OxA-21706	UF	39400	1000	[137]
La Ferrassie	•6	MAMS 17585	UF	32 450	130	[144]
La Ferrassie	•6	MAMS 21208	UF	36 300	300	[144]
La Ferrassie	6	MAMS 25522	UF	36 590	390	[144]
La Ferrassie	6	MAMS 16373	UF	37 380	390	[144]
La Ferrassie	•6	MAMS 21207	UF	38 910	390	[144]
La Ferrassie	6	MAMS 25523	UF	39 000	510	[144]
La Ferrassie	6	MAMS 25524	UF	40 770	650	[144]
La Ferrassie	6	MAMS 21206	UF	40 890	500	[144]
Foradada	IV	Beta-414539[b]	ABA	31900	200	[36]
Foradada	IV	OxA-X-2650-9	ABOx-SC	34300	1000	[36]

Foradada	IV	OxA-X-2649-9	ABOx-SC	34490	320	[36]
Foradada	IV	Beta-435465	ABA	34750	240	[36]
La Ferrassie	6	FER 2	OSL	42200	2900	[145]
Vieux Coutets	Châtelperronian	VC OSL 1	OSL	44600	4800	[146]
Aranbaltza	US4b	AAM16-10	OSL	43500	2900	This paper
Vieux Coutets	Châtelperronian	Bdx 11972	TL	41000	2000	[146]
Les Cottés	6	LC10-13	OSL	38400	1800	[147]
Les Cottés	6	LC10-15	OSL	43300	2300	[147]
Les Cottés	6	LC11-2	OSL	43100	2100	[147]

[a] The Grotte du Renne ^{14}C ages of Higham et al. [13] have not been included in our Bayesian modelling analysis owing to their poor internal consistency and a lack of consensus on the stratigraphic interpretations of this dataset (see discussions in [12, 14, 136-137]).

[b] This sample was not included in the Bayesian model as it yielded a significantly younger age in comparison to two associated samples from layer IV that were subjected to more rigorous (ABOx-SC) pre-treatment procedures (OxA-X-2650-9 and OxA-X-2649-9). The systematic ^{14}C age offset for Beta-414539 is therefore potentially indicative of incomplete removal of organic contaminants by the standard ABA pretreatment procedure (see related discussions in [36]). The other ABA-pretreated sample from layer IV (Beta-435465) yielded an age that is consistent with the associated ABOx-SC samples. This sample does not appear to have suffered from the same potential contamination issues as Beta-414539, and has therefore been retained for Bayesian modelling purposes.

Table S14. Directly dated Neandertal remains from sites in France with associated Châtelperronian layers*.

**Individual/Site**	**Anatomical region**	**Lab reference**	**Method**	**Age (^{14}C yr B.P.)**	**±1σ Err**	**Reference**
Saint Césaire[a]	Tibia	OxA-18099	UF	36200	750	[14]
Grotte du Renne (Arcy-sur-Cure) (AR-14)	Cranial fragment[b]	MAMS-25149	UF	36840	660	[12]
La Ferrassie 8[a,c]	Indeterminate[b]	ETH-99102	UF	36170	220	[15]

[*] Other recent directly dated remains from Goyet (Belgium; [148]) are not included here as there is currently no evidence for the Châtelperronian culture in this area.

[a] These samples have not been included in the Bayesian model owing to uncertainties in the stratigraphic relationship of the dated fossil remains and the Châtelperronian layers at Le Ferrassie and Saint Césaire (see discussions in [11, 15] and references therein).

[b] Identified as hominins using ZooMS, and further recognized as Neandertals by aDNA.

[c] Bone found in close association with the LF8 hominin remains.

Table S15. Bayesian model summary.

	Unmodelled (BP)					Modelled (BP)						Indices<br>Amodel 56.8<br>Aoverall 57.7			
	from	to	%	sigma	median	from	to	%	sigma	median	Acomb	A	L	P	C
Boundary End Châtelperronian						41450	38340	95.44997	790	39720					98.3
Duration Châtelperronian						43760	39220	95.44997	1180	41550					99.8
N Aranbaltza II US4b OSL-AAM16-10	49240	37630	95.44997	2900	43430	43860	39500	95.44997	1100	41800		115.2		95.4	99.9
Châtelperronian Ekain OxA-34930						41860	39010	95.44997	700	40250					99.7
Duration Châtelperronian Vieux Coutets						43630	39360	95.44997	1100	41560					99.8
Duration Châtelperronian La Ferrasie						43650	40680	95.44997	750	42090					99.9
Duration Châtelperronian Foradada						42270	39220	95.44997	800	40210					99.4
Duration Châtelperronian La Quina Amont						43700	40190	95.44997	830	42340					99.7
Duration Châtelperronian Grotte des Fées						43750	40910	95.44997	680	42690					99.7
Duration Châtelperronian Labeko Koba						42920	41240	95.44997	400	42160					99.8
Duration Châtelperronian Les Cottés						43830	39970	95.44997	950	41910					99.9
Duration Châtelperronian Saint-Césaire						42760	40160	95.44997	600	41500					99.8
Duration Grotte du Renne Layer VIII						41740	40640	95.44997	280	41270					100
Duration Grotte du Renne Layer IX+X						43400	41390	95.44997	540	42270					99.9
Phase Châtelperronian timing															
Boundary Start Châtelperronian						44900	41960	95.44997	730	43230					98.3
Sequence Châtelperronian timing															
Boundary End Châtelperronian Ekain						41590	-38030	95.44997	23950	28270					95.8
R_Date Châtelperronian Ekain OxA-34930	40710	37730	95.44997	740	39490	41860	39010	95.44997	700	40250		77.8		88.4	99.7
Boundary Start Châtelperronian Ekain						126140	39190	95.44998	26350	52570					95.7
Sequence Châtelperronian Ekain															
Boundary End Châtelperronian Vieux Coutets						43640	25610	95.44997	7570	39410					94.6
Duration Châtelperronian Vieux Coutets						43630	39360	95.44997	1100	41560					99.8
N TL-Bdx-11972	44940	36930	95.44997	2000	40930	44460	38030	95.44997	1550	41350		112.2		95.5	100
N VC-OSL1	54140	34930	95.44997	4800	44530	48860	36900	95.44997	2670	41890		112.7		95.2	99.9
Phase Châtelperronian Vieux Coutets															
Boundary Start Châtelperronian Vieux Coutets						58760	39300	95.44997	8150	43800					94.3
Sequence Châtelperronian Vieux Coutets															
Boundary End Châtelperronian La Ferrasie						41860	39830	95.44997	570	41100					97.8
Duration Châtelperronian La Ferrasie						43650	40680	95.44997	750	42090					99.9
R_Date MAMS-21208	41890	40850	95.44997	260	41330	42000	40940	95.44997	310	41480		94.4		95.2	99.9
R_Date MAMS-25522	42080	40990	95.44997	280	41540	42130	41070	95.44997	300	41630		104		96.2	99.9
R_Date MAMS-16373	42370	41470	95.44997	220	42010	42390	41450	95.44997	250	42010		102.8		96.5	99.9
R_Date MAMS-21207	42970	42290	95.44997	180	42610	43020	42260	95.44997	210	42610		101.5		96.4	99.9
R_Date MAMS-25523	43280	42200	95.44997	280	42670	43290	42190	95.44997	290	42660		102.7		96.4	99.9
R_Date MAMS-25524	44750	42870	95.44997	510	43800	44410	42720	95.44997	440	43390		97.8		94.8	99.9
R_Date MAMS-21206	44630	43040	95.44997	430	43870	44460	42830	95.44997	440	43450		90.4		93.6	99.9
N OSL-FER2	47940	36330	95.44997	2900	42140	44390	40640	95.44997	940	42440		134.5		95.6	100
Phase Châtelperronian La Ferrasie															
Boundary Start Châtelperronian La Ferrasie						45330	42990	95.44997	630	43910					98.8
Sequence Châtelperronian La Ferrasie															
Boundary End Châtelperronian Foradada						40430	36650	95.44997	1600	39390					93.6
Duration Châtelperronian Foradada						42270	39220	95.44997	800	40210					99.4
R_Date OxA-X-2650-9	41420	36920	95.44997	1170	39300	41230	38420	95.44997	630	39870		119.8		96.1	99.6
R_Date OxA-X-2649-9	40480	39110	95.44997	350	39670	40470	39220	95.44997	330	39790		101		96.8	99.8
R_Date Beta-435465	40500	39400	95.44997	290	39910	40490	39420	95.44997	290	39900		106.4		97.1	99.8
Phase Châtelperronian Foradada															
Boundary Start Châtelperronian Foradada						43930	39460	95.44997	1820	40600					90.6
Sequence Châtelperronian Foradada															
Boundary End Châtelperronian La Quina Amont						43300	35540	95.44997	4100	41620					94.2
Duration Châtelperronian La Quina Amont						43700	40190	95.44997	830	42340					99.7
R_Date OxA-21707	43830	41220	95.44997	550	42330	43390	41360	95.44997	470	42420		109.3		96.4	99.9
R_Date OxA-21706	44580	42120	95.44997	680	43110	44140	42000	95.44997	550	42740		111.2		95.9	99.9
Phase Châtelperronian La Quina Amont															
Boundary Start Châtelperronian La Quina Amont						49280	41970	95.44997	4100	43400					94.7
Sequence Châtelperronian La Quina Amont															
Boundary End Châtelperronian Grotte des Fées						43160	39320	95.44997	1570	42280					92.2
Duration Châtelperronian Grotte des Fées						43750	40910	95.44997	680	42690					99.7
R_Date OxA-13622	43880	42230	95.44997	400	42760	43530	42240	95.44997	310	42790		110.9		97.1	99.9
R_Date OxA-14320	43160	42380	95.44997	210	42740	43180	42410	95.44997	210	42770		102.8		97.1	99.9
R_Date OxA-13621	44600	42870	95.44997	470	43730	44250	42570	95.44997	430	43180		80.7		93.4	99.8
Phase Châtelperronian Grotte des Fées															
Boundary Start Châtelperronian Grotte des Fées						45980	42600	95.44997	1470	43530					93.1
Sequence Châtelperronian Grotte des Fées															
Boundary End Châtelperronian Labeko Koba						42510	40640	95.44997	580	41880					93.7
Duration Châtelperronian Labeko Koba						42920	41240	95.44997	400	42160					99.8
R_Date OxA-22560	42780	40980	95.44997	450	41940	42640	41460	95.44997	280	42140		119.3		97.3	99.8
R_Date OxA-22563	43250	41010	95.44997	540	42160	42730	41510	95.44997	290	42180		131.8		97.5	99.8
R_Date OxA-22561	43790	41130	95.44997	550	42280	42760	41550	95.44997	280	42200		130.7		97.4	99.9
R_Date OxA-22562	43830	41220	95.44997	550	42330	42780	41570	95.44997	280	42210		128.7		97.4	99.8
Phase Châtelperronian Labeko Koba															
Boundary Start Châtelperronian Labeko Koba						43610	41800	95.44997	530	42450					95.3
Sequence Châtelperronian Labeko Koba															

Boundary End Châtelperronian Les Cottés						41730	38030	95.44997	1040	40560					97.7
Duration Châtelperronian Les Cottés						43830	39970	95.44997	950	41910					99.9
N OSL-LC10-13	41950	34740	95.44997	1800	38340	43640	38790	95.44997	1120	41300		45.2		92.9	100
N OSL-LC11-2	47240	38830	95.44997	2100	43040	45460	40320	95.44997	1330	42910		119.6		95.7	100
N OSL-LC10-15	47850	38640	95.44997	2300	43240	45550	40310	95.44997	1370	42990		121.4		95.6	100
R_Date EVA-11	41740	40910	95.44997	200	41260	41850	40880	95.44997	330	41300		97.7		95.1	99.8
R_Date EVA-12	42520	41200	95.44997	340	41950	42570	41180	95.44997	380	41960		102		96	99.9
R_Date EVA-13	42490	42110	95.44997	90	42300	42520	42070	95.44997	170	42300		101.1		96.1	99.9
R_Date MAMS-10803	42710	42230	95.44997	120	42460	42770	42200	95.44997	210	42460		100.9		95.8	99.9
R_Date EVA-21	44570	43300	95.44997	350	44100	44610	43210	95.44997	440	44070		98.1		95	99.8
R_Date EVA-5	45650	44460	95.44997	300	44990	45690	42980	95.44997	660	44790		93.2		87.6	99.7
Phase Châtelperronian Les Cottés															
Boundary Start Châtelperronian Les Cottés						47050	43420	95.44997	820	45210					98.2
Sequence Châtelperronian Les Cottés															
Boundary End Châtelperronian Saint-Césaire						42050	38770	95.44997	1260	40960					94.4
Duration Châtelperronian Saint-Césaire						42760	40160	95.44997	600	41500					99.8
R_Date OxA-21699	42090	39850	95.44997	580	41050	42160	40440	95.44997	440	41380		107.1		96.6	99.8
R_Date OxA-21700	42400	40440	95.44997	500	41520	42240	40690	95.44997	390	41510		113.4		97	99.9
R_Date OxA-21636	43020	40400	95.44997	630	41800	42450	40680	95.44997	440	41580		110.3		96.7	99.9
Phase Châtelperronian Saint-Césaire															
Boundary Start Châtelperronian Saint-Césaire						44150	40850	95.44997	1250	42010					94.6
Sequence Châtelperronian Saint-Césaire															
Boundary End Châtelperronian Grotte du Renne						41640	40120	95.44997	400	41000					99.5
Duration Grotte du Renne Layer VIII						41740	40640	95.44997	280	41270					100
R_Date EVA-54	41230	39720	95.44997	390	40530	41680	40520	95.44997	290	41140		53.2		90.1	99.9
R_Date EVA-52	41890	40290	95.44997	390	41070	41690	40730	95.44997	240	41230		115.3		97.6	100
R_Date EVA-53	41980	40580	95.44997	360	41280	41700	40820	95.44997	220	41280		124.2		97.8	100
R_Date EVA-55	42150	40940	95.44997	310	41560	41720	40930	95.44997	200	41330		101.2		97.3	100
R_Date EVA-56	42620	41490	95.44997	260	42140	41770	40940	95.44997	210	41400		10.9		80.1	100
Phase Grotte du Renne Layer VIII															
Boundary Grotte du Renne Layer IX+X/VIII						41850	41130	95.44997	180	41530					100
Duration Grotte du Renne Layer IX+X						43400	41390	95.44997	540	42270					99.9
R_Date EVA-29	41110	40110	95.44997	250	40680	43340	41120	95.44997	580	41990		5.8		6.6	99.8
R_Date EVA-25	41780	40830	95.44997	230	41250	42560	41140	95.44997	340	41680		41.3		85.4	99.9
R_Date EVA-38	41940	41120	95.44997	210	41520	42070	41290	95.44997	240	41720		90.1		94.5	99.9
R_Date EVA-32	42060	41290	95.44997	200	41690	42120	41400	95.44997	210	41790		106.9		96.5	100
R_Date EVA-23	42130	41220	95.44997	240	41690	42180	41390	95.44997	220	41810		107.8		96.5	99.9
R_Date MAMS-25149	42370	40770	95.44997	410	41640	42420	41350	95.44997	280	41880		110.5		96.2	99.9
R_Date EVA-40	42350	41800	95.44997	140	42090	42360	41780	95.44997	160	42090		102.8		97.1	100
R_Date EVA-36	42430	41900	95.44997	130	42180	42450	41870	95.44997	160	42180		102.1		97	100
R_Date EVA-30	42490	42020	95.44997	110	42260	42520	41990	95.44997	140	42260		101.9		97	100
R_Date EVA-42	42550	42040	95.44997	120	42300	42580	42010	95.44997	160	42300		101.8		96.8	100
R_Date EVA-24	42700	42160	95.44997	130	42410	42730	42140	95.44997	160	42410		101.8		96.9	100
R_Date EVA-41	42840	42270	95.44997	140	42530	42870	42250	95.44997	170	42530		101.8		96.9	100
R_Date EVA-43	42980	42350	95.44997	160	42650	43010	42330	95.44997	180	42650		101.9		96.8	100
R_Date EVA-48	42980	42370	95.44997	160	42660	43010	42350	95.44997	180	42660		101.9		96.9	99.9
R_Date EVA-35	43090	42410	95.44997	180	42730	43120	42400	95.44997	190	42730		102.4		96.9	100
R_Date EVA-44	43130	42420	95.44997	190	42750	43150	42410	95.44997	190	42750		102.7		96.9	100
R_Date EVA-31	43110	42430	95.44997	180	42750	43140	42420	95.44997	190	42750		102.5		96.9	100
R_Date EVA-26	43190	42460	95.44997	200	42800	43200	42450	95.44997	190	42800		103.1		96.9	100
R_Date EVA-37	43270	42460	95.44997	220	42830	43240	42470	95.44997	200	42820		103.9		96.9	100
R_Date EVA-47	43900	42590	95.44997	330	43010	43520	42540	95.44997	240	42970		113.1		96.8	100
R_Date EVA-46	44020	42690	95.44997	360	43150	43660	42640	95.44997	250	43060		118.7		96.7	100
R_Date EVA-51	44400	42510	95.44997	520	43350	43730	42450	95.44997	320	43060		114.1		96.3	100
R_Date EVA-27	44210	42830	95.44997	380	43440	43780	42750	95.44997	260	43180		112.2		96.1	100
R_Date EVA-34	44330	42970	95.44997	370	43630	43840	42840	95.44997	260	43270		97.9		95.1	99.9
R_Date EVA-49	45060	42790	95.44997	600	43860	43850	42640	95.44997	300	43230		92.6		94.7	99.9
R_Date EVA-28	44550	43150	95.44997	390	43920	44010	42830	95.44997	290	43340		70.4		91.9	99.9
R_Date EVA-33	44600	43140	95.44997	400	43950	44010	42820	95.44997	290	43330		68.8		91.9	99.9
Phase Grotte du Renne Layer IX+X															
Boundary Start Châtelperronian Grotte du Renne						44060	43160	95.44997	230	43540					99.7
Sequence Châtelperronian Grotte du Renne															
U(0,4)	3.99E-17	4	95.44997	1.1431	2	2.212	3.896	95.44997	0.482633	3.004		100			99.7
T(5)	-2.65	2.65	95.44997	1.29081	2.05E-12				1.21455	0.285					99.9
Outlier_Model General						-1960	2700	95.44997	1170	230					99.7
Curve IntCal20															

Supplementary References

86. Jordá Cerdá, F., 1963. El Paleolítico superior cantábrico y sus industrias. Saitabi 13, 3–22.
87. McCollough, M.C.R., 1971. Perigordian facies in the Upper Palaeolithic of Cantabria . Thesis Ph.D. University of Pennsylvania, Michigan.
88. Regalado Bueno, E., 2012. Prospección y estudio de patrimonio arqueológico en la comarca de Uribe Kosta. Isturitz. Cuadernos de Sección. Prehistoria-Arqueologia 12, 115–134.
89. Rios-Garaizar, J., Cubas, M., Garate Maidagan, D., Libano Silvente, I., Ugarte Cuétara, A., Vega López, S., Regalado Bueno, E., San Emeterio Gómez, A., Tapia, J., García Moreno, A., Trebolazabala Hurtado, A., Aketxe Agirre, A., 2018a. Late prehistoric coastal settlement patterns in the Cantabrian region, northern Spain. Antiquity 92. https://doi.org/10.15184/aqy.2018.152.
90. Miall, A., 1996. The Geology of Fluvial Deposits. Sedimentary Facies, Basin Analysis, and Petroleum Geology. xvi + 582 pp. Berlin, Heidelberg, New York, London, Paris, Tokyo, Hong Kong: Springer-Verlag.
91. Cruz-Sanjulián, J.J., García Mondéjar, J., Granda, J.M., Pujalte, V. (1983). Características y evolución de unos depósitos de "Cliff-Top" localizados sobre la rasa costera vizcaína. Thalassass, 2, 31-34.
92. Silverman, B. W., 1986. Density estimation for statistics and data analysis, Chapman and Hall, London. http://dx.doi.org/10.1007/978-1-4899-3324-9.
93. Sánchez-Romero, L., Benito-Calvo, A., Pérez-González, A., Santonja, M., 2016. Assessment of Accumulation Processes at the Middle Pleistocene Site of Ambrona (Soria, Spain). Density and Orientation Patterns in Spatial Datasets Derived from Excavations Conducted from the 1960s to the Present. PLOS ONE 11, e0167595.
94. Sánchez-Romero, L., Benito-Calvo, A., Marín-Arroyo, A.B., Agudo-Pérez, L., Karampaglidis, T., Rios-Garaizar, J., 2020. New insights for understanding spatial patterning and formation processes of the Neanderthal occupation in theAmalda I cave (Gipuzkoa, Spain). Scientific Reports 10, 8733. https://doi.org/10.1038/s41598-020-65364-8.
95. Tarriño-Vinagre, A., 2006. El sílex en la Cuenca Vasco-Cantábrica y Pirineo Navarro: caracterización y su aprovechamiento en la Prehistoria, Monografías del Mueso Nacional y Centro de Investigación de Altamira. Ministerio de Cultura, Madrid.
96. Tarriño Vinagre, A., Muñoz-Fernández, E., Elorrieta Baigorri, I., Normand, C., Rasines del Río, P., García-Rojas, M., Pérez-Bartolomé, M., 2016. El sílex en la cuenca vasco-cantábrica y el Pirineo occidental: materia prima lítica en la Prehistoria. Cuadernos de Prehistoria y Arqueología de la Universidad de Granada 26, 191–228.
97. Brock, F., Higham, T., Ditchfield, P., Ramsey, C.B., 2010. Current Pretreatment Methods for AMS Radiocarbon Dating at the Oxford Radiocarbon Accelerator Unit (Orau). Radiocarbon 52, 103–112. https://doi.org/10.1017/S0033822200045069
98. Reimer, P., Austin, W. E. N., Bard, E., Bayliss, A., Blackwell, P. G., Bronk Ramsey, C., Butzin, M., Cheng, H., Lawrence Edwards, R., Friedrich, M., Grootes, P. M., Guilderson, T. P., Hajdas, I., Heaton, T. J., Hogg, A. G., Hughen, K. A., Kromer, B., Manning, S. W., Muscheler, R., ... Talamo, S. 2020. The IntCal20 Northern

Hemisphere radiocarbon age calibration curve (0-55 cal kBP). Radiocarbon, 62(4), 725. https://doi.org/10.1017/RDC.2020.41.
99. Higham, T., 2011. European Middle and Upper Palaeolithic radiocarbon dates are often older than they look: problems with previous dates and some remedies. Antiquity, 85(327), 235-249.
100. Becerra-Valdivia, L., Higham, T., 2020. The timing and effect of the earliest human arrivals in North America. Nature 584, 93–97. https://doi.org/10.1038/s41586-020-2491-6.
101. Arnold, L.J., Demuro, M., Navazo Ruiz, M., 2012. Empirical insights into multi-grain averaging effects from 'pseudo' single-grain OSL measurements. Radiation Measurements 47, 652-658.
102. Murray, A.S., Wintle, A., 2000. Luminescence dating of quartz using an improved single-aliquot regenerative-dose protocol. Radiation Measurements 32, 57–73.
103. Duller, G.A.T., 2003. Distinguishing quartz and feldspar in single grain luminescence measurements. Radiation Measurements 37, 161–165.
104. Galbraith, R.F., 2002. A note on the variance of a background-corrected OSL count. Ancient TL 20, 49–51.
105. Jacobs, Z., Duller, G.A.T., Wintle, A.G., 2006. Interpretation of single-grain De distributions and calculation of De. Radiation Measurements 41, 264–277.
106. Duller, G.A.T., 2007. Assessing the error on equivalent dose estimates derived from single aliquot regenerative dose measurements. Ancient TL 25, 15–24.
107. Arnold, L.J., Duval, M., Falguères, C., Bahain, J.-J., Demuro, M. 2012. Portable gamma spectrometry with cerium-doped lanthanum bromide scintillators: Suitability assessments for luminescence and electron spin resonance dating applications. Radiation Measurements 47, 6-18.
108. Bøtter-Jensen, L., Mejdahl, M., 1988. Assessment of beta dose-rate using a GM multicounter system. Nuclear Tracks and Radiation Measurements 14, 187-191.
109. Potts, P.J., Thompson, M., Chenery, S.R.N., Webb, P.C., Kasper, H.U., 2003. Geopt13 - An International Proficiency Test for Analytical Geochemistry Laboratories - Report on Round 13 / July 2003 (Köln Loess). International Association of Geoanalysts.
110. Brennan, B.J., 2003. Beta doses to spherical grains. Radiation Measurements 37, 299-303.
111. Prescott, J.R., Hutton, J.T., 1994. Cosmic ray contributions to dose rates for luminescence and ESR dating: large depths and long-term time variations. Radiation Measurements 23, 497–500.
112. Mejdahl, V., 1987. Internal radioactivity in quartz and feldspar grains. Ancient TL 5, 10–17.
113. Bowler, J.M., Johnston, H., Olley, J.M., Prescott, J.R., Roberts, R.G., Shawcross, W., Spooner, N.A., 2003. New ages for human occupation and climate change at Lake Mungo, Australia. Nature 421, 837–840.
114. Jacobs, Z., Duller, G.A.T., Wintle, A.G., Henshilwood, C.S., 2006. Extending the chronology of deposits at Blombos Cave, South Africa, back to 140 ka using optical dating of single and multiple grains of quartz. Journal of Human Evolution 51, 255-273.

115. Pawley, S.M., Bailey, R.M., Rose, J., Moorlock, B.S.P., Hamblin, R.J.O., Booth, S.J., Lee, J.R., 2008. Age limits on Middle Pleistocene glacial sediments from OSL dating, north Norfolk, UK. Quaternary Science Reviews 27, 1363-1377.
116. Rees-Jones, J., 1995. Optical dating of young sediments using fine-grain quartz. Ancient TL 13, 9-14.
117. Rees-Jones, J., Tite, M.S., 1997. Optical dating results for British archaeological sediments. Archaeometry 39, 177-187.
118. Guérin, G., Mercier, M., Adamiec, G., 2011. Dose-rate conversion factors: update. Ancient TL 29, 5-8.
119. Mejdahl, V., 1979. Thermoluminescence dating: beta-dose attenuation in quartz grains. Archaeometry 21, 61-72.
120. Aitken, M.J., 1985. Thermoluminescence Dating. Academic Press, London, 359 p.
121. Readhead, M.L., 1987. Thermoluminescence dose rate data and dating equations for the case of disequilibrium in the decay series. Nuclear Tracks and Radiation Measurements 13, 197-207.
122. Berger, G.W., Pérez-González, A., Carbonell, E., Arsuaga, J.L., Bermúdez de Castro, J.- M., Ku, T.-L., 2008. Luminescence chronology of cave sediments at the Atapuerca paleoanthropological site, Spain. Journal of Human Evolution 55, 300-311.
123. Demuro, M., Arnold, L.J., Parés, J.M., Sala, R., 2015. Extended-range luminescence chronologies suggest potentially complex bone accumulation histories at the Early-to-Middle Pleistocene palaeontological site of Huéscar-1 (Guadix-Baza basin, Spain). Quaternary International 389, 191-212.
124. Méndez-Quintas, E., Santonja, M., Pérez-González, A., Duval, M., Demuro, M., Arnold, L.J., 2018. First evidence of an extensive Acheulean large cutting tool accumulation in Europe from Porto Maior (Galicia, Spain). Scientific Reports 8, 3082.
125. Arnold L.J., Roberts, R.G., 2009. Stochastic modelling of multi-grain equivalent dose (De) distributions: Implications for OSL dating of sediment mixtures. Quaternary Geochronology 4, 204–230.
126. Bailey, R.M., and Arnold, L.J. 2006. Statistical modelling of single grain quartz De distributions and an assessment of procedures for estimating burial dose. Quaternary Science Reviews 25, 2475-2502.
127. Arnold, L.J., Roberts, R.G., 2011. Paper I – Optically stimulated luminescence (OSL) dating of perennially frozen deposits in north-central Siberia: OSL characteristics of quartz grains and methodological considerations regarding their suitability for dating. Boreas 40, 389–416. https://doi.org/https://doi.org/10.1111/j.1502-3885.2011.00209.x
128. Arnold, L.J., Roberts, R.G., Galbraith, R.F., DeLong, S.B., 2009. A revised burial dose estimation procedure for optical dating of young and modern-age sediments. Quaternary Geochronology 4, 306-325.
129. Galbraith, R.F., Roberts, R.G., Laslett, G.M., Yoshida, H., Olley, J.M., 1999. Optical dating of single and multiple grains of quartz from Jinmium rock shelter, northern Australia: Part I, Experimental design and statistical models. Archaeometry 41, 339-364.

130. Arnold, L. J., Roberts, R. G., MacPhee, R. D. E., Willerslev, E., Tikhonov, A. N., Brock, F., 2008. Optical dating of perennially frozen deposits associated with preserved ancient plant and animal DNA in north-central Siberia. Quatern. Geochronol. 3, 114–136.
131. Arnold, L. J., Roberts, R. G., MacPhee, R. D. E., Haile, J. S., Brock, F., Möller, P., Froese, D. G., Tikhonov, A. N., Chivas, A. R., Gilbert, M. T. P., Willerslev, E., 2011. Paper II – Dirt, dates and DNA: OSL and radiocarbon chronologies of perennially frozen sediments in Siberia and their implications for sedimentary ancient DNA studies. Boreas 40, 417–445.
132. Nathan, R.P., Thomas, P.J., Jain, M., Murray, A.S., Rhodes, E.J., 2003. Environmental dose rate heterogeneity of beta radiation and its implications for luminescence dating: Monte Carlo modelling and experimental validation. Radiation Measurements 37, 305–313.
133. Arnold, L.J., Demuro, M., Parés, J.M., Arsuaga, J.L., Aranburu, A., Bermúdez de Castro, J.M., Carbonell, E. 2014. Luminescence dating and palaeomagnetic age constraint on hominins from Sima de los Huesos, Atapuerca, Spain. Journal of Human Evolution 67, 85-107.
134. Olley, J.M., Caitcheon, G.G., Roberts, R.G., 1999. The origin of dose distributions in fluvial sediments, and the prospect of dating single grains of quartz from fluvial deposits using optically stimulated luminescence. Radiation Measurements 30, 207-217.
135. Arnold, L.J., Bailey, R.M., Tucker, G.E., 2007. Statistical treatment of fluvial dose distributions from southern Colorado arroyo deposits. Quaternary Geochronology 2, 162–167.
136. Caron F, d'Errico F, Del Moral P, Santos F, Zilhão J (2011) The reality of Neandertal symbolic behavior at the Grotte du Renne, Arcy-sur-Cure, France. PLoS One 6(6):e21545.
137. Higham, T., Douka, K., Wood, R., Ramsey, C.B., Brock, F., Basell, L., Camps, M., Arrizabalaga, A., Baena, J., Barroso-Ruiz, C., Bergman, C., Boitard, C., Boscato, P., Caparros, M., Conard, N.J., Draily, C., Froment, A., Galvan, B., Gambassini, P., Garcia-Moreno, A., Grimaldi, S., Haesaerts, P., Holt, B., Iriarte-Chiapusso, M.-J., Jelinek, A., Jorda Pardo, J.F., Maillo-Fernandez, J.-M., Marom, A., Maroto, J., Menendez, M., Metz, L., Morin, E., Moroni, A., Negrino, F., Panagopoulou, E., Peresani, M., Pirson, S., de la Rasilla, M., Riel-Salvatore, J., Ronchitelli, A., Santamaria, D., Semal, P., Slimak, L., Soler, J., Soler, N., Villaluenga, A., Pinhasi, R., Jacobi, R., 2014. The timing and spatiotemporal patterning of Neanderthal disappearance. Nature 512, 306–309.
138. Hammer, Ø., Harper, D.A.T., Ryan, P.D., 2001. PAST: Paleontological statistics software package for education and data analysis. Paleontologia Electronica 4.
139. Hansen, V., Murray, A., Buylaert, J.-P., Yeo, E.-Y., Thomsen, K., 2015. A new irradiated quartz for beta source calibration. Radiation measurements 81, 123-127.
140. Galbraith, R.F., Green, P.F., 1990. Estimating the component ages in a finite mixture. Nuclear Tracks and Radiation Measurements 17, 197-206.
141. Prescott, J.R., Hutton, J.T., 1994. Cosmic ray contributions to dose rates for luminescence and ESR dating: Large depths and long-term time variations. Radiation

Measurements 23, 497–500. https://doi.org/https://doi.org/10.1016/1350-4487(94)90086-8
142. Talamo, S., Soressi, M., Roussel, M., Richards, M., Hublin, J.-J., 2012. A radiocarbon chronology for the complete Middle to Upper Palaeolithic transitional sequence of Les Cottés (France). Journal of Archaeological Science 39, 175–183. https://doi.org/http://dx.doi.org/10.1016/j.jas.2011.09.019.
143. Gravina, B., Mellars, P., Ramsey, C.B., 2005. Radiocarbon dating of interstratified Neanderthal and early modern human occupations at the Chatelperronian type-site. Nature 438, 51–56.
144. Talamo, S., Aldeias, V., Goldberg, P., Chiotti, L., Dibble, H. L., Guérin, G., Hublin, J. J., Madelaine, S., Maria, R., Sandgathe, D., Steele, T. E., Turq, A. & McPherron, S. J. P. 2020: The new 14C chronology for the Palaeolithic site of La Ferrassie, France: the disappearance of Neanderthals and the arrival of Homo sapiens in France. Journal of Quaternary Science 35, 961-973.
145. Guérin, G., Frouin, M., Talamo, S., Aldeias, V., Bruxelles, L., Chiotti, L., Dibble, H.L., Goldberg, P., Hublin, J.-J., Jain, M., Lahaye, C., Madelaine, S., Maureille, B., McPherron, S.J.P., Mercier, N., Murray, A.S., Sandgathe, D., Steele, T.E., Thomsen, K.J., Turq, A., 2015. A multi-method luminescence dating of the Palaeolithic sequence of La Ferrassie based on new excavations adjacent to the La Ferrassie 1 and 2 skeletons. Journal of Archaeological Science 58, 147–166. https://doi.org/10.1016/J.JAS.2015.01.019.
146. Lahaye, C., Guibert, P., Viellevigne, E., Guérin, G., 2018. Compte-rendu des mesures effectuées en vue de la datation du site de Vieux Coutet (Creysse, Dordogne), in: Ortega, I. (Ed.), Le Vieux Coutet I. INRAP - Institut National de Recherches Archéologiques Préventives, Bègles, pp. 77–79.
147. Jacobs, Z., Li, B., Jankowski, N., Soressi, M., 2015. Testing of a single grain OSL chronology across the Middle to Upper Palaeolithic transition at Les Cottés (France). Journal of Archaeological Science 54, 110–122. https://doi.org/https://doi.org/10.1016/j.jas.2014.11.020.
148. Rougier, H., Crevecoeur, I., Beauval, C., Posth, C., Flas, D., Wißing, C., Furtwängler, A., Germonpré, M., Gómez-Olivencia, A., Semal, P., van der Plicht, J., Bocherens, H. & Krause, J. 2016: Neandertal cannibalism and Neandertal bones used as tools in Northern Europe. Scientific Reports 6, 29005.
